# Diboramacrocycles: reversible borole dimerisation–dissociation systems†

**DOI:** 10.1039/d1sc06908j

**Published:** 2022-02-04

**Authors:** Sonja Fuchs, Arumugam Jayaraman, Ivo Krummenacher, Laura Haley, Marta Baštovanović, Maximilian Fest, Krzysztof Radacki, Holger Helten, Holger Braunschweig

**Affiliations:** Institute for Inorganic Chemistry, Julius-Maximilians-Universität Würzburg Am Hubland 97074 Würzburg Germany h.braunschweig@uni-wuerzburg.de; Institute for Sustainable Chemistry & Catalysis with Boron, Julius-Maximilians-Universität Würzburg Am Hubland 97074 Würzburg Germany

## Abstract

We report that the outcome of the tin–boron exchange reaction of a mixed thiophene-benzo-fused stannole with aryldibromoboranes is associated with the steric bulk of the aryl substituent of the borane reagent, leading to either boroles or large diboracycles as products. NMR spectroscopic studies indicate that the two products can reversibly interconvert in solution, and mechanistic density functional theory (DFT) calculations reveal boroles to be intermediates in the formation of the diboracyclic products. The addition of Lewis bases to the diboracycles leads to the corresponding borole adducts, demonstrating that they react as “masked” boroles. Additionally, the reaction of the title compounds with a series of organic azides affords complex heteropropellanes, formally 2 : 1 borole-azide adducts, that deviate from the usual BN aromatic compounds formed *via* nitrogen atom insertion into the boroles.

## Abstract 2

Diboramacrocycles are a new form of borole dimers, participating in various addition reactions as “masked” boroles. The reaction of a less crowded diboramacrocycle with organic azides affords unprecedented complex heteropropellanes.

## Graphical abstract

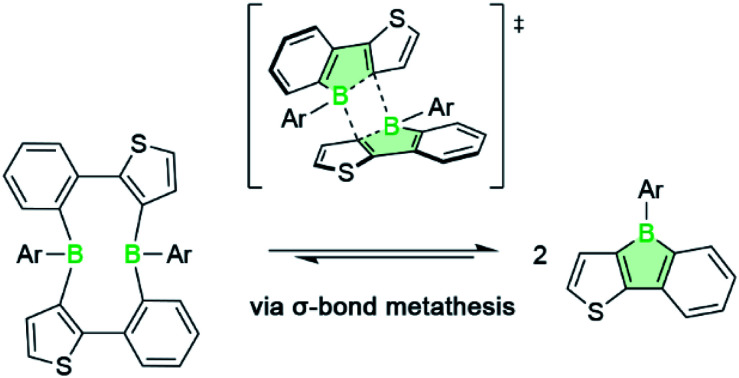

## Introduction

The class of five-membered boroles continues to be of great interest beyond their antiaromatic π-electron conjugation.^1^ Due to a range of additional attributes, such as their high Lewis acidity, electron-accepting ability, and chromophoric properties, boroles are a promising platform for diverse applications ranging from reagents in chemical synthesis to electronic materials.^2,3^

**Scheme 1:**
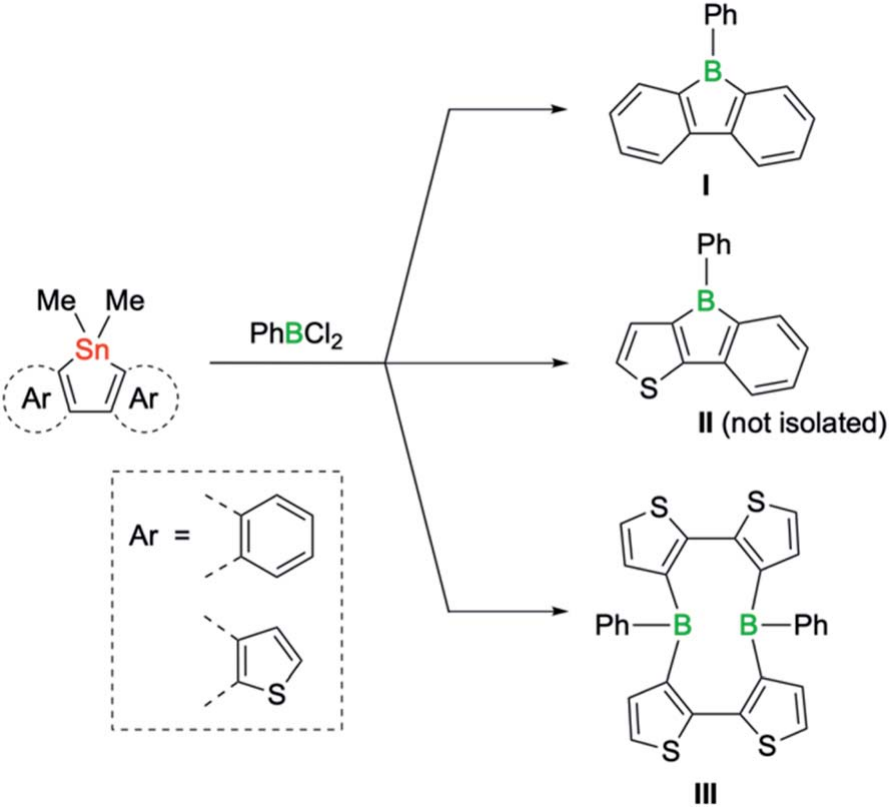
Products of the tin–boron exchange reaction of fused stannoles with PhBCl_2_, according to He *et al.*^6^

Intense research during the past decade has demonstrated that the properties of boroles are highly sensitive to the nature of the substituents surrounding the five-membered ring. Typically highly reactive in nature, air and moisture stable derivatives could be synthesised by incorporating the bulky 2,4,6-tris(trifluoromethyl)phenyl group on the boron atom, while the steric bulk of the remaining substituents suppresses dimerisation *via* Diels–Alder cycloadditions.^4^ Likewise, the extension of the borole π system by fusion of aromatic groups can be used to modulate key properties.^3^ For example, dibenzannulated boroles, also known as 9-borafluorenes,^3*b*^ display reduced antiaromaticity, whereas dithiophene-fused boroles possess increased antiaromaticity that even surpasses the antiaromaticity of their non-fused derivatives.^5^ The greater antiaromaticity and consequently lower stability of the doubly thiophene-fused derivatives is also reflected in their preparation.^5^ A systematic study by the group of He recently revealed that the success of the classic tin–boron exchange reaction is correlated with the degree of antiaromaticity of the boroles (Scheme 1).^6^ While the reaction of doubly benzo-fused and mixed thiophene-benzo-fused stannole precursors with dichloro(phenyl)borane led to the desired boroles (I and II), the tin–boron exchange with the doubly thiophene-fused precursor led instead to a diboracyclic structure (III), where the unfavourable cyclic four-electron delocalisation is avoided.^6^ Such macrocyclic ring structures, in which the boron atoms are doubly biaryl-bridged, have been observed by Piers^7^ and also postulated by Wagner as intermediates in dimerisation pathways of 9-*H*-9-borafluorene.^8^ Although boroles have not been previously thought to be playing a role in tin–boron exchange reactions where large diboracycles are formed,^6^ they might thus represent viable intermediates.

In this contribution, we present experimental insights into how steric factors influence borole and diboramacrocycle formation, respectively, in the tin–boron exchange reaction of a mixed thiophene-benzo-fused stannole with a series of aryldibromoboranes. Computational studies identifying the mechanistic steps of the transformation clarify the relationship between the two products and establish boroles as intermediates in the formation of the large diboracyclic ring systems. In view of the poorly studied influence of fused thiophene rings on the reactivity of boroles, we further canvassed the reactivity of a derivative towards a series of organic azides. In contrast to its dibenzo analog, where the reaction yields BN-phenanthrenes by ring expansion of the central borole unit,^9^ the reaction proceeds by a distinct mechanism to structurally complex heteropropellanes.

## Results and discussion

**Scheme 2:**
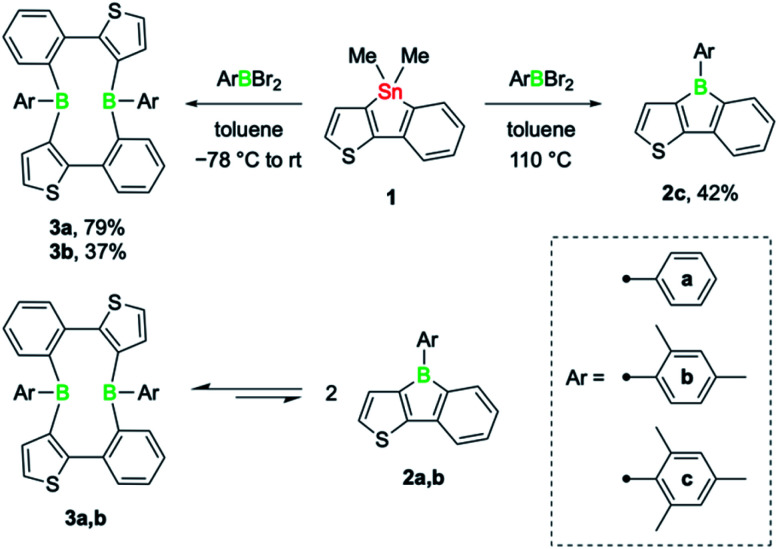
Isolated products of the tin–boron exchange reaction between stannole 1 and various aryldibromoboranes (top), and dynamic equilibrium between the cyclic dimers 3a, b and the corresponding annulated boroles 2a, b in solution (bottom).

**Fig. 1:**
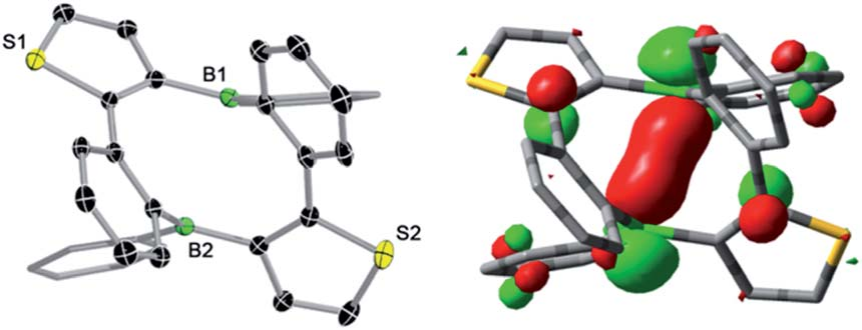
Molecular structure of 3a with thermal ellipsoids at 50% probability (left) and LUMO of 3a (isovalue = 0.05, right).

The tin–boron exchange reaction of the mixed thiophene-benzo-fused stannole 1^6^ with different aryldibromoboranes^10^ produced either borole (2c) or diboracyclic products (3a, b; Scheme 2, top). Selective formation of the borole 2c was observed only with the sterically most hindered dibromo(mesityl)borane, whereas employing the less-hindered phenyl- and 2,4-xylyldibromoboranes led to the diboracycles 3a and 3b in 79% and 37% isolated yield, respectively. The products were obtained as red (2c) or yellow solids (3a, b) and were characterised by single-crystal X-ray diffraction analysis (see ESI† for details). The structural parameters of the borole 2c, and the diboracycle 3a (shown in Fig. 1) and 3b, consisting of a ten-membered ring structure, are comparable to experimental data for related systems.^5–7,11^ The large heterocycles 3a and 3b, with the composition of formal borole dimers, adopt a boat-like conformation with transannular boron–boron interactions of 2.779(4)/2.790(4) Å (3a) and 3.291(4) Å (3b).^6,7^ In particular, the short boron–boron distance in 3a suggests a significant overlap of the vacant p_*z*_ orbitals of the neighbouring boron centres.^12^ This is also seen in the LUMO of 3a, as shown in Fig. 1.

Solution ^1^H and ^11^B NMR spectroscopy at room temperature revealed that 3a and 3b dissociate readily to the monomeric boroles 2a and 2b, respectively (Scheme 2, bottom). While 3b exhibits only very broad resonances in the ^1^H and ^11^B NMR spectra, distinct ^11^B NMR signals for the monomer (2a: *δ*(^11^B) = 60.0 ppm) and dimer (3a: *δ*(^11^B) = 64.0 ppm) can be observed upon dissolution of 3a in toluene-*d*_8_. On heating, the two peaks become better resolved, clearly indicating the presence of a monomer–dimer equilibrium in solution. Although readily observable by NMR spectroscopy, borole 2a was not isolable.^6^ The higher yield of phenyl-substituted dimer 3a relative to that of the xylyl derivative 3b suggests that a sterically less hindered boron environment favours dimer formation. Indeed, computations performed at the B3LYP-D3BJ/6-311g++(d,p)/SCRF(SMD = benzene)//B3LYP-D3BJ/6-31g(d,p) level of theory showed that thermodynamically, dimer formation is favoured in all cases, and that the relative stability decreases with increasing steric hindrance of the B-aryl substituent (phenyl, Δ*G* = −7.1 kcal mol^−1^; xylyl, Δ*G* = −5.7 kcal mol^−1^; mesityl, Δ*G* = −1.3 kcal mol^−1^). Although thermodynamically slightly favoured, dimer formation in the case of the mesityl derivative might be more challenging due to steric hindrance and we have not observed any dimer formation experimentally.

Borole 2c is characterised by a lowest-energy absorption at 457 nm in hexane, which is slightly hypsochromically shifted compared to its π-extended benzothiophene-substituted analog (*λ*_max_ = 474 nm).^5*b*^ Like many other fused borole derivatives, 2c was found to be fluorescent.^5*a*,13^ It exhibits a broad emission with a peak maximum around 590 nm and a fluorescence lifetime of *τ* = 11.5 ns (see Fig. S4 in the ESI† for details).

**Scheme 3:**
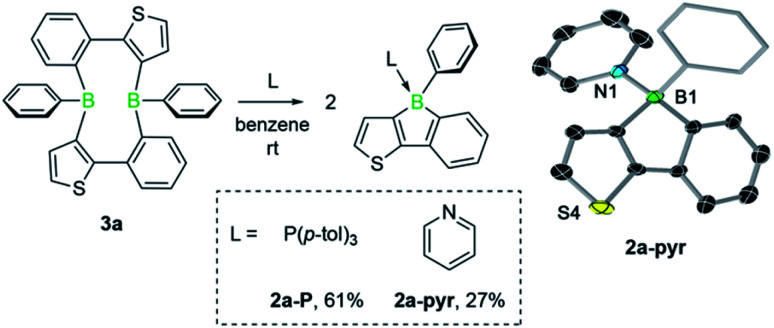
Lewis base adducts of 3a and molecular structure of 2a-pyr. Displacement ellipsoids are shown at the 50% probability level. The ellipsoids of the phenyl substituent, hydrogen atoms and solvent molecules are omitted for clarity. Selected bond lengths (Å) and angles (°): B1–N1 1.623(5), B1–C1 1.616(4); C1–B1–N1 109.46.

We then investigated the reactivity of diboracycle 3a toward adduct formation with Lewis bases. Addition of two equivalents of pyridine and tri(*p*-tolyl)phosphine to 3a led to complete consumption of 3a and formation of the borole adducts 2a-pyr and 2a-P, respectively (Scheme 3). The ^11^B NMR spectroscopic signals of both adducts appear as relatively sharp singlets at 1.1 ppm (2a-pyr) and −10.0 ppm (2a-P), respectively. Their structures were unambiguously confirmed by single-crystal X-ray crystallography (see Scheme 3 for the structure of 2a-pyr). The structural parameters, including the boron–heteroatom bonds and the geometry around the boron atoms, are consistent with other structurally characterised pyridine- and phosphine-borole adducts.^14,15^ The reactivity of 3a towards the Lewis base pyridine differs from that of the corresponding doubly thiophene-fused derivative, for which the bis(pyridine) adduct was observed under comparable conditions.^6^ The selective formation of the borole-pyridine adduct 2a-pyr from 3a implies that diboracycle 3a rapidly dissociates to borole 2a before reacting with pyridine. It is thus able to serve as a source for the borole monomer, in analogy to the Diels–Alder dimers of boroles.^16^

**Fig. 2:**
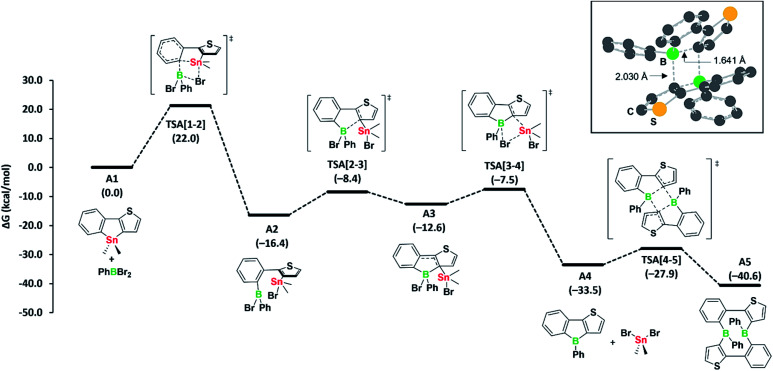
Computed mechanism for diboracycle formation *via* tin–boron exchange. Free energies in parentheses are in kcal mol^−1^. DFT-optimised structure with key distances of the transition state responsible for the borole-to-diboracycle interconversion (TSA[4-5]) is shown in the inset in the top right corner.

Our observation that the boroles 2a, b exist in equilibrium with their cyclic dimers 3a, b led us to study the mechanism of the tin–boron exchange in more detail. We assumed that the tin–boron exchange would first yield the borole structures, later transforming into the ten-membered diboracyclic rings. To address this possibility, we carried out mechanistic computations on such a reaction pathway for the phenyl derivative (Fig. 2).

The formation of the borole A4 (corresponding to 2a) and the by-product dibromo(dimethyl)stannane from the reaction of the stannole with dibromo(phenyl)borane is exergonic by −33.5 kcal mol^−1^. Kinetically, the first transmetalation step (A1 to A2) is rate-limiting, with a barrier of 22.0 kcal mol^−1^. This barrier is consistent with the reaction proceeding at room temperature. The remaining steps leading to borole A4 have only small barriers. Remarkably, the transformation of borole A4 to its dimer A5 (corresponding to 3a) *via* transition state TSA[4-5], involving a σ-bond metathesis between the B–C bonds of two boroles, shows that the barrier is small (Δ*G*^‡^ = 5.6 kcal mol^−1^). As the dimer A5 is only slightly more stable than borole A4 (Δ*G* = −7.1 kcal mol^−1^), its reversion to borole A4 is relatively facile due to the associated low barrier of only 12.7 kcal mol^−1^. Accordingly, the two species are expected to be in equilibrium at room temperature, consistent with the experimental observations. An alternative formation of dimer A5*via* intermolecular boron–tin exchange between two molecules of A2, which would circumvent a borole intermediate,^6^ might incur a large barrier, mainly due to the high entropic penalty associated with the intermolecular reaction. Thus, we have not further considered this possibility. Overall, we propose that the formation of the ten-membered diboracycles 3a and 3b from the tin–boron exchange reaction of stannole 1 proceeds *via* a borole intermediate.

The facile conversion of A4 to A5 can be mainly ascribed to the unfavourable antiaromatic character and low steric shielding in A4, both of which contribute to the ease of dimerisation. The nucleus-independent chemical shifts (NICS) computed for a small set of boroles show that the antiaromatic character within diarene-fused boroles increases with the number of thiophenes fused to the borole ring (see Table S1 in the ESI†), a result that is consistent with previous findings of Yamaguchi^5^ and He.^6^ These observed trends are also harmonious with the formation of diboracycles – and not boroles – from the tin–boron exchange of related doubly thiophene-fused stannoles, as the corresponding boroles would exhibit a strong antiaromatic character.^6^

**Fig. 3:**
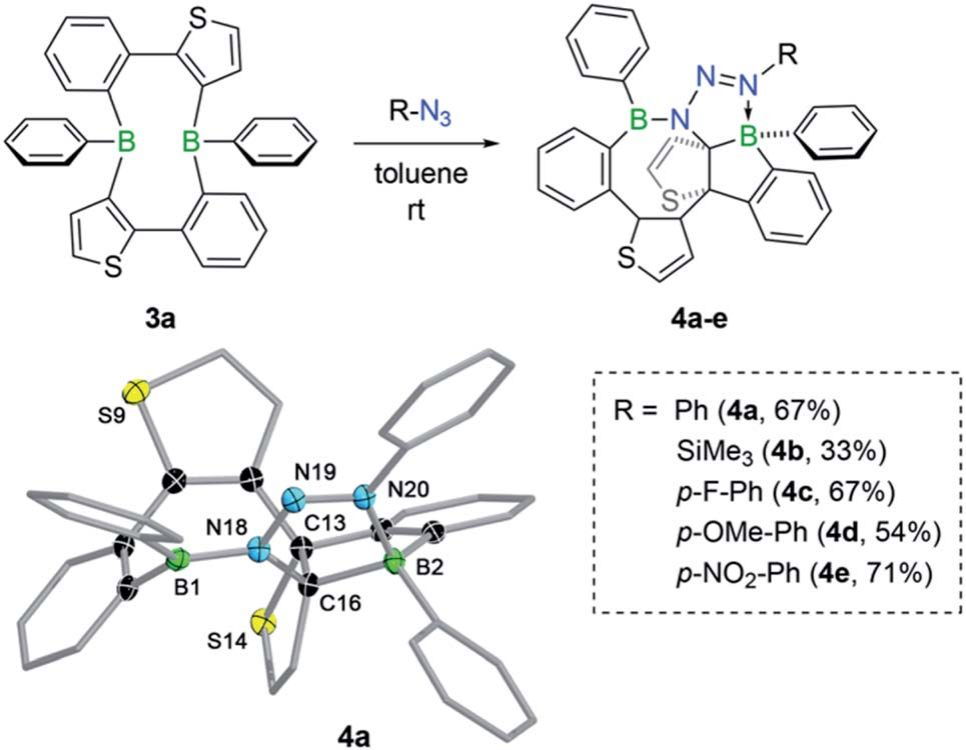
Reactivity of 3a towards a series of organic azides and molecular structure of 4a. Displacement ellipsoids are shown at the 50% probability level. Some ellipsoids and hydrogen atoms have been removed for clarity. Selected bond lengths (Å) and angles (°): B1–N18 1.456(2), B21–N20 1.617(2), N18–N19 1.339(2), N19–N20 1.277(2), N18–C16 1.491(2), C16–B21 1.661(2), C13–C16 1.592(2); N18–N19–N20 111.6(1).

Like non-fused boroles,^2*b*,17^ annulated boroles have been shown to undergo ring expansion with organic azides to generate BN heteroaromatic compounds.^3,18^ Deviations from the predominant denitrogenative pathway have been observed by Martin from the reaction between phenyl azide and 9-phenyl-9-borafluorene, the dibenzo analog of borole 2a.^18*b*^ In this case, the reaction instead proceeded without extrusion of molecular nitrogen and afforded a diazene-functionalised BN-phenanthrene. We were thus eager to study the effect of the fused thiophene group on the reactivity of the “masked” borole 3a towards azides. Treatment of 3a with 1 equiv. of phenyl azide yielded a new product with one broad (*δ* = 52.2 ppm) and one sharp singlet (*δ* = 7.3 ppm) in the ^11^B NMR spectrum, indicative of the presence of both three- and four-coordinate boron centers, respectively. LIFDI-MS results were consistent with a 2 : 1 borole-azide adduct, specifying that the reaction proceeded without loss of dinitrogen. After work-up, product 4a was obtained as an orange solid in 67% yield. Due to the complex ^1^H NMR spectrum, the structure of 4a was finally revealed by a single-crystal X-ray diffraction study (Fig. 3). The solid-state structure confirmed the presence of an intact azide group, which was found to bridge the two boron atoms through the nitrogen atoms at either end. The polycyclic structure of 4a can be described as a heteropropellane as the C13–C16 bond is part of three different heterocyclic ring systems. As signaled by NMR spectroscopy, the molecular structure contains a boron atom in a trigonal-planar (sum of bond angles of 359.9(1)°) as well as in a tetrahedral geometry (sum of bond angles of 336.9(1)°). The latter is pyramidalised due to the coordination of the substituent-bearing nitrogen atom of the azide; the corresponding B–N bond length of 1.617(2) Å is typical of a dative boron–nitrogen interaction.^19^

**Fig. 4:**
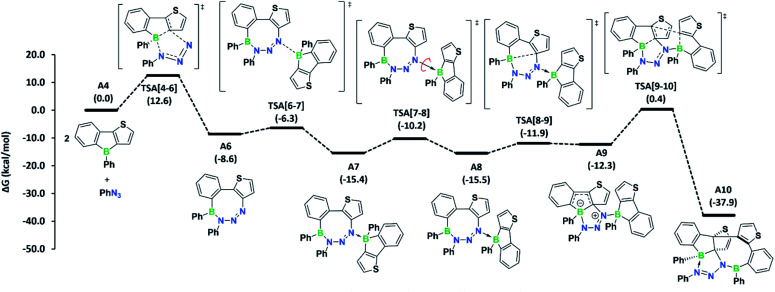
Mechanism computed for azide adduct formation at the B3LYP-D3BJ/6-311g++**/SMD(C_6_H_6_)//B3LYP-D3BJ/6-31g** level of theory. Free energies in parentheses are in kcal mol^−1^.

To determine if the reaction is more general in scope, we explored the reaction of 3a with a series of other organic azides, including aryl azides with different substitution patterns and trimethylsilyl azide (Fig. 3). In all cases, the reaction proceeded within minutes at room temperature and invariably yielded the appropriate heteropropellane compounds in moderate to good yields. The reaction outcome thus appears largely unaffected by the electronic and steric effects of the organic azides. Using an excess rather than one equivalent of the aryl azides led to the same outcome. The identities of products 4b–e were corroborated by single-crystal X-ray crystallography, heteronuclear NMR spectroscopy, and LIFDI mass spectrometry. The unusual propellane products 4a–e are distinct from the common ring expansion products derived from formal nitrene insertion into the antiaromatic borole and from other divergent outcomes of the reaction of boroles with organic azides.^2*b*,3,17,18^ However, the fact that only one borole unit underwent ring expansion with the organic azide could suggest that a reaction pathway involving an initial borole adduct of an eight-membered BN_3_C_4_ heterocycle, as previously observed by Martin,^17*b*^ might be followed. To rationalise the mechanistic steps involved in forming the propellanes 4a–e, we performed DFT calculations on the reaction of 2a with phenyl azide. We considered borole 2a rather than the cyclic dimer 3a for the computations, as adduct formation with phenyl azide would preferentially occur with borole 2a, which is in equilibrium with 3a in the solution (*vide supra*). The computed mechanism, together with the free energy profile, is shown in Fig. 4. The first step entails a [2+3] addition between the endocyclic B–C bond of the borole and the outer nitrogen atoms of the azide, leading to the ring-expanded eight-membered BN_3_C_4_ heterocycle A6. Lewis acid complexation of A6 by a second borole unit through the more basic sp^2^ nitrogen atom yields adduct A7. Subsequent rotation of the borole unit around the newly formed B–N bond affords rotamer A8, which in turn triggers intramolecular B–C bond formation to give the bicyclic intermediate A9. This step corresponds to the re-formation of the previously broken B–C bond in the initial borole ring expansion with the azide. In the last step, the complexed borole in A9 expands its ring *via* C–C coupling between two thiophene rings to form the product A10 (corresponding to 4a). Thermodynamically, the overall reaction is highly favoured (Δ*G* = −37.9 kcal mol^−1^). Kinetically, the first and last steps of the reaction sequence are equally rate-determining with barriers of 12.6 kcal mol^−1^ and 12.7 kcal mol^−1^, respectively. These barriers can be easily attained under the reaction conditions and are in line with the rapidly occurring reaction at room temperature.

Conceptually, much of the reaction path resembles that derived from the reaction of boroles with azides, resulting in the formation of 1,2-azaborinines.^17*b,e*^ In fact, an adduct similar to A8 has been isolated by the group of Martin as a kinetic product in the reaction of pentaphenylborole with trimethylsilyl azide, which yields an 1,2-azaborinine as the thermodynamic product.^17*b*^

Thermodynamically, in our case, the formation of the corresponding 1,2-azaborinine derivative from borole 2a and phenyl azide is remarkably exergonic (Δ*G* = −95.7 kcal mol^−1^), and thus its formation is highly favoured over heteropropellane A10. Yet, the lack of 1,2-azaborinine formation, even after heating the reaction solution to 110 °C for 12 h, signifies that the formation of A9, and eventually A10, is highly favoured due to the low energy barriers associated with their formation. Moreover, the reactivity of 3a towards organic azides further establishes the potential of biaryl-bridged bis(boranes) to act as sources of monomeric boroles.

## Conclusions

This study on the tin–boron exchange of a mixed thiophene-benzo-fused stannole with a series of sterically varied aryldibromoboranes has shown that mainly sterics of the B-aryl substituent dictate the product formation between boroles and diboracycles, their macrocyclic dimers. With decreasing steric demand of the boron substituent, the reaction diverts away from the anticipated tin–boron exchange product – the boroles – and produces diboracycles instead. NMR spectroscopic data and computational insights show that the two products are in equilibrium at room temperature, demonstrating that borole dimerisation and dissociation can occur reversibly. Reactivity studies towards Lewis bases and organic azides uncovered that these cyclic dimers behave as “masked” boroles. With a series of organic azides, an unprecedented mode of ring expansion afforded heteropropellanes containing all three nitrogen atoms of the azide. Mechanistic investigations by quantum chemical calculations revealed that the reaction proceeds through a borole-stabilised eight-membered BN_3_C_4_ heterocycle, followed by B–C bond formation and C–C coupling of the two thiophene units. Remarkably, the formation of heteropropellanes from the reaction of thiophene-benzo fused boroles with organic azides seems quite general for a variety of azide substituents.

## Data availability

Full experimental and computational details are provided as part of the ESI.†

## Author contributions

H. B. supervised the project. S. F., L. H., and M. B. carried out the synthetic work. S. F. and K. R. carried out the X-ray crystallographic analyses. M. F. and H. H. contributed to the photophysical studies. I. K. carried out the CV measurements. A. J. carried out the computational studies. I. K. and A. J. prepared the manuscript. S. F., A. J., M. F. and I. K. prepared the ESI. All authors discussed the results and commented on the manuscript.

## Conflicts of interest

There are no conflicts to declare.

## Supplementary Material

SC-013-D1SC06908J-s001

SC-013-D1SC06908J-s002
