# Supplementary material for: Diboramacrocycles: reversible borole dimerisation–dissociation systems
Source: Chem Sci. 2022 Feb 4;13(10):2932–8. doi: 10.1039/d1sc06908j (PMC8905844; doi:10.1039/d1sc06908j)
Supplement: SC-013-D1SC06908J-s001 [file SC-013-D1SC06908J-s001.pdf]

Electronic Supplementary Material (ESI) for Chemical Science.
This journal is © The Royal Society of Chemistry 2022

Supporting Information

Diboramacrocycles: reversible borole dimerisation-dissociation systems

Sonja Fuchs, Arumugam Jayaraman, Ivo Krummenacher, Laura Haley, Marta Baštovanovič, Maximilian Fest, Krzysztof Radacki, Holger Helten and Holger Braunschweig*

Institute for Inorganic Chemistry and Institute for Sustainable Chemistry & Catalysis with Boron, Julius-Maximilians-Universität Würzburg, Am Hubland, 97074 Würzburg, Germany

Table of Contents

1. General Considerations 2
  1.1 Photophysical Measurements 2
  1.2 Electrochemical measurements 3
2. Synthetic Details and Characterisation Data 4
  2.1 Compound **2c** 4
  2.2 Compound **3a** 7
  2.3 Compound **3b** 11
  2.4 Reactivity studies of **3a** with Lewis bases 12
  2.5 Compound **4a** 18
  2.6 Compound **4b** 21
  2.6 Compound **4c** 24
  2.6 Compound **4d** 27
  2.7 Compound **4e** 29
3. Crystallographic details 32
4. Computational details 38
  4.1 Optimised structures, Cartesian coordinates, and energies 39
    4.1.1 Thermodynamic stability of boroles and their diboracyclic dimers 39
    4.1.2 Mechanism for the tin-boron exchange reaction 47
    4.1.3 Mechanism for the azide addition 65
    4.1.4 Different boroles probed for antiaromaticity 93
5. References 101

1. GENERAL CONSIDERATIONS

All manipulations were performed either under an atmosphere of dry argon or in vacuo using standard Schlenk line or glovebox techniques. Deuterated solvents were dried over molecular sieves and degassed by three freeze-pump-thaw cycles prior to use. All other solvents were distilled and degassed from appropriate drying agents. Solvents (both deuterated and non-deuterated) were stored under argon over activated 4 Å molecular sieves. All glassware was oven dried prior to use. NMR spectra were acquired on a Bruker Avance 500 NMR spectrometer (^{1}H: 500.1 MHz, ^{11}B: 160.5 Hz, ^{13}C{^{1}H}: 125.8 MHz) and a Bruker Avance 400 NMR spectrometer (^{1}H: 400.1 MHz, ^{11}B: 128.5 Hz, ^{13}C: 101 MHz, ^{31}P: 202.5 MHz). Chemical shifts (δ) are given in ppm and are internally referenced to the carbon nuclei (^{13}C{^{1}H}) or residual protons (^{1}H) of the solvent. All spectra were acquired at 298 K unless stated otherwise. Resonances are given as singlet (s), doublet (d), triplet (t) or multiplet (m). Microanalyses (C, H, N) were performed on an Elementar vario MICRO cube elemental analyser. High resolution mass spectrometry was performed on a Thermo Scientific Exactive Plus spectrometer using a LIFDI 700 source from Linden CMS. The stannoles,[1-2] dibromophenyl-,[3] dibromoxylyl-,[3] and dibromomesitylborane[3] were synthesised according to, or analogous to, known literature procedures.

1.1 Photophysical Measurements

UV-vis measurements were carried out using a METTLER TOLEDO UV5 spectrometer. The emission spectra were recorded using an Edinburgh Instruments FLSP920 spectrometer equipped with a double monochromator for both excitation and emission, operating in right-angle geometry mode, and all spectra were fully corrected for the spectral response of the instrument. All solutions used for photophysical measurements had a concentration lower than 2×10^{-5} M to minimise inner filter effects during fluorescence measurements. For solution-state measurements, the longest-wavelength absorption maximum of the compound in the respective solvent was chosen as the excitation wavelength.

Fluorescence lifetimes were recorded using the time-correlated single-photon counting (TCSPC) method using the same FLSP920 spectrometer described above. Solutions were excited with a picosecond pulsed diode laser at an emission maximum of 472.6 nm. The full width at half maximum (FWHM) of the laser pulses were ca. 91 ps, while the instrument response function (IRF) had a FWHM of ca. 1.0 ns, measured from the scatter of a Ludox solution at the excitation wavelength. Decays were recorded to 10000 counts in the peak channel with a record length of at least 4800 channels. The band pass of the monochromator was adjusted to give a signal count rate of ca. 15 kHz. Iterative reconvolution of the IRF with one decay function and non-linear least-squares analysis were used to analyse the data. The quality of the fit was judged by the calculated value of the reduced χ^2 and visual inspection of the weighted residuals.

1.2 Electrochemical measurements

All cyclic voltammetry experiments were conducted in an argon-filled glovebox using a Gamry Instruments Reference 600 potentiostat. A standard three-electrode cell configuration was employed using a platinum disk working electrode, a platinum wire counter electrode, and a silver wire reference electrode separated by a Vycor frit, serving as the reference electrode. The redox potentials are referenced to the ferrocene/ferrocenium ([Fc/Fc$^+$]) redox couple by using decamethylferrocene ([Cp$^*{}_2$Fe]; $E_{1/2}$ = –0.532 V in CH_2Cl_2) as an internal standard. Tetra-*n*-butylammonium hexafluorophosphate ([*n*Bu_4N][PF_6]) was employed as the supporting electrolyte. Compensation for resistive losses (*iR* drop) was employed for all measurements.

2. Synthetic Details and Characterisation Data

2.1 Compound 2c

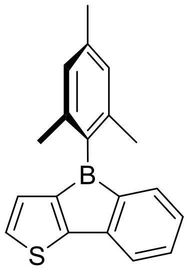

In a Schlenk tube, stannole **1** (500 mg, 1.63 mmol, 1.0 eq.) and dibromo(mesityl)borane (270 mg, 977 μmol, 1.0 eq.) were dissolved in toluene (10 mL) and the mixture was stirred at 110 °C for 72 h. All volatiles of the red solution were removed under reduced pressure. The remaining solid was recrystallised from hexane at –78 °C to afford **2c** as a red solid in 42% yield (197 mg, 684 mmol).

^{1}H NMR (400.1 MHz, C_6D_6): δ(ppm) = 7.05-7.03 (m, 1H, thio-C*H*), 6.80 (m, 2H, Mes-C*H*), 6.78-6.76 (m, 2H, Ar*H*), 6.70-6.63 (m, 2H, Ar*H*), 6.55 (d, 1H, $^3J_{HH}$ = 4.7 Hz, Ar*H*), 2.22 (s, 6H, Mes-CH_3), 2.20 (s, 3H, Mes-CH_3). **^{11}B NMR** (128.5 MHz, C_6D_6): δ(ppm) = 68 (br, s). **^{13}C{^{1}H} NMR** (100.7 MHz, C_6D_6): δ(ppm) = 169.7 (Ar-C_q), 147.45 (Ar-C_q), 138.9 (Mes-C_q), 138.5 (Mes-C_q), 134.3 (thio-*C*H), 134.0 (Ar-*C*H), 130.3 (Ar-*C*H), 128.6 (Ar-*C*H), 127.9 (Ar-*C*H), 127.6 (thio-*C*H), 119.1 (Ar-*C*H), 22.6 (Mes-CH_3), 21.3 (Mes-CH_3). The quaternary carbon atoms connected to the boron atom were not detected.

HRMS (LIFDI, m/z): $[C_{19}H_{17}BS]^+$ calculated: 288.1133; found: 288.1133.

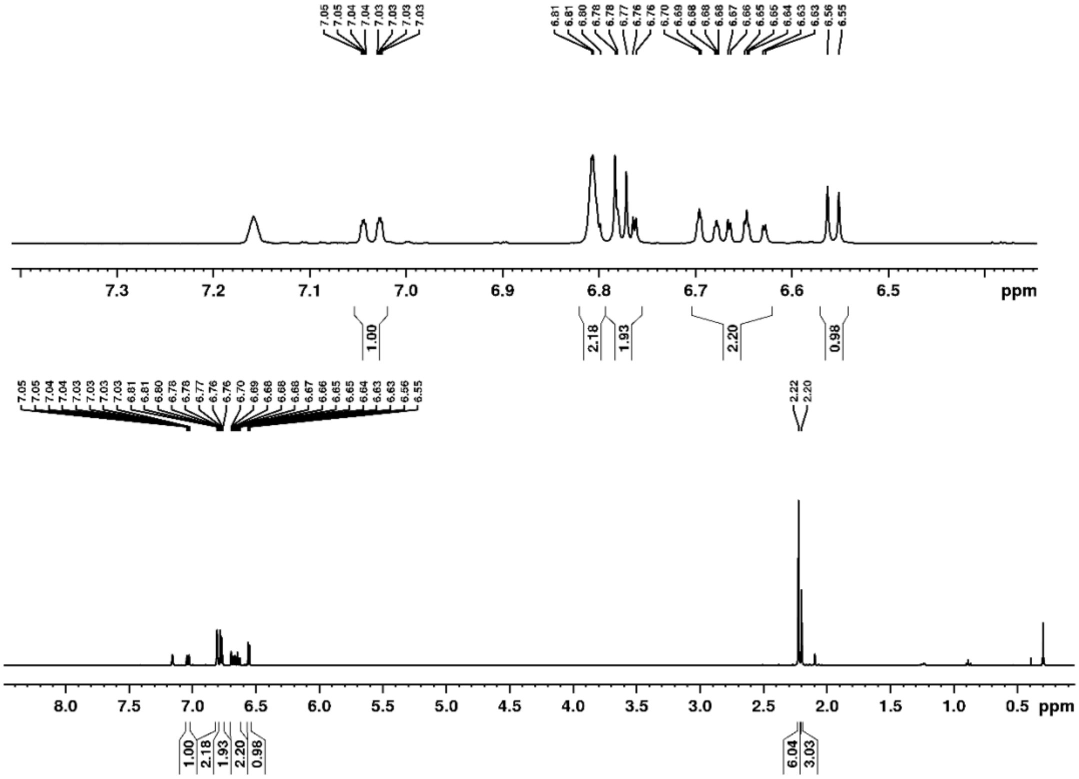

Figure S1. ^{1}H NMR spectrum (500.1 MHz) of **2c** in C_6D_6.

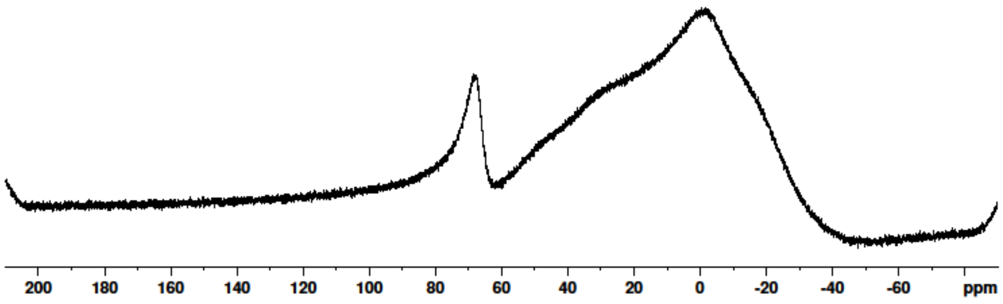

Figure S2. ^{11}B NMR spectrum (128.5 MHz) of **2c** in C_6D_6.

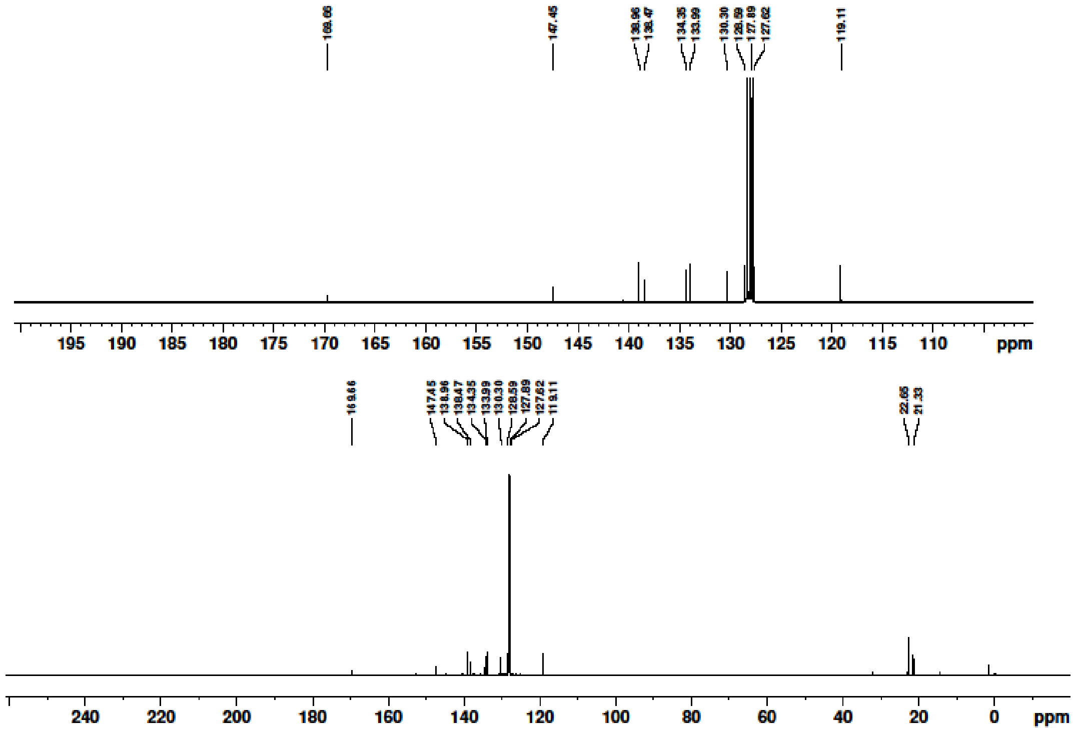

Figure S3. ^{13}C NMR spectra (100.7 MHz) of **2c** in C_6D_6.

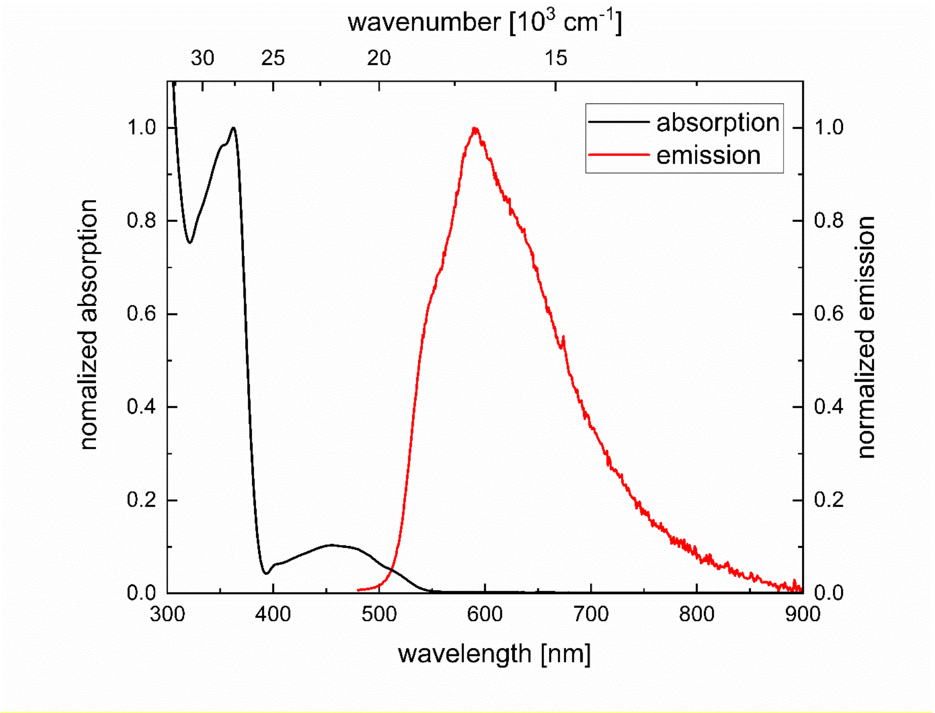

Figure S4. Absorbance and emission spectra of **2c** in hexane. Photophysical data: lowest-energy absorption at 457 nm, emission at 551 nm (shoulder) and 590 nm, fluorescence lifetime τ = 11.5 ns, quantum yield could not be determined due to slow decomposition.

2.2 Compound 3a

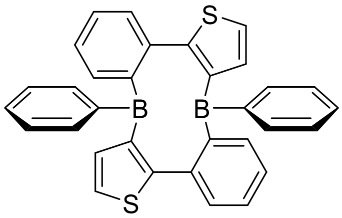

In a Schlenk tube, stannole **1** (1.50 g, 4.89 mmol, 1.0 eq.) was dissolved in toluene (50 mL). At –78 °C, a solution of dibromo(phenyl)borane (1.21 g, 4.89 mmol, 1.0 eq.) in toluene (100 mL) was added over a period of 30 min. The mixture was stirred and allowed to warm to room temperature. All volatiles were removed under reduced pressure and the remaining solid washed three times with hexane. Compound **3a** (950 mg, 1.93 mmol) was isolated as a yellow solid in 79% yield.

^{1}H NMR (500.1 MHz, toluene-d_8, −40 °C): δ (ppm) = 7.49 (d, 4H, $^{3}J_{HH}$ = 7.6 Hz), 7.06 (m, 4H, toluene residue below signal), 6.95 (dt, 1H, $^{3}J_{HH}$ = 7.5 Hz, $^{4}J_{HH}$ = 1.5 Hz), 6.84 (m, 2H), 6.79 (m, 1H), 6.50 (d, 1H, $^{3}J_{HH}$ = 5.1 Hz). **^{11}B NMR** (160.5 MHz, toluene-d_8, 0 °C): δ (ppm) = 64 (s). **^{13}C{^{1}H} NMR** (125.8 MHz, toluene-d_8, −40 °C): δ (ppm) = 163.5 (Ar-C_q), 150.7 (Ar-C_q), 148.9 (Ar-C_q), 141.4 (Ar-C_q), 139.5(Ar-C_q), 137.5, 137.1, 137.0, 137.0, 131.8, 130.5, 127.2, 126.7, 123.9

Elemental analysis: Calculated: C: 78.08%, H: 4.50%, S: 13.03%; found: C: 77.25%, H: 4.40%, S: 12.86%.

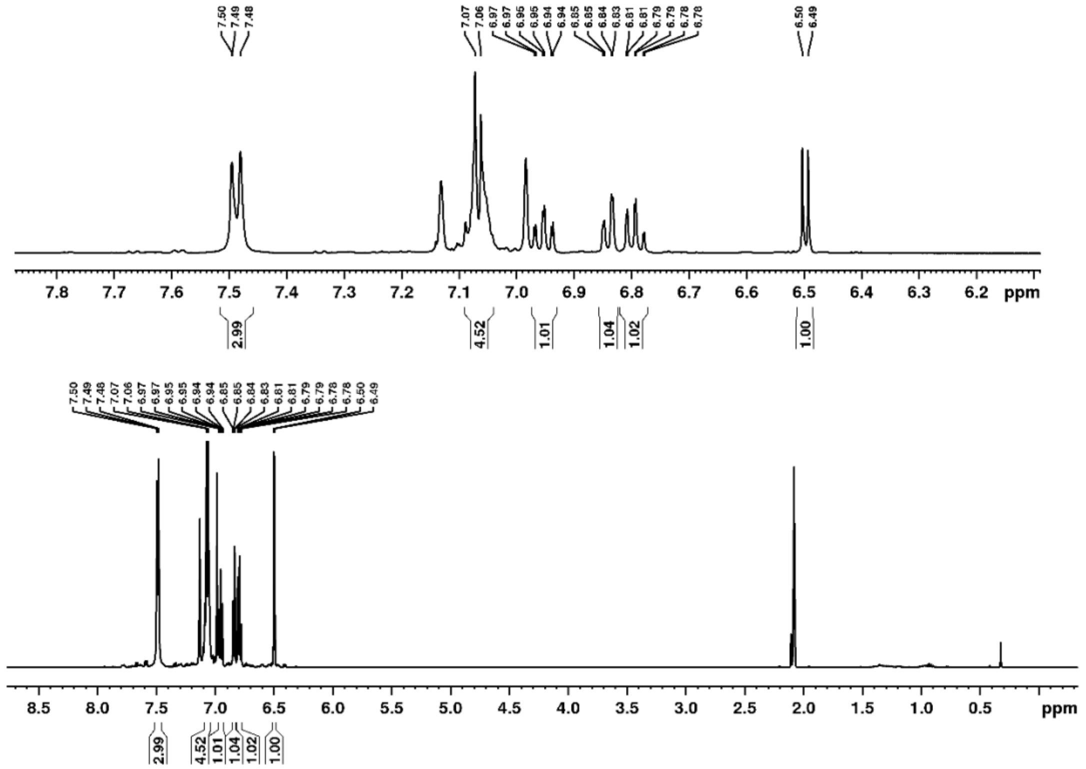

Figure S5. ^{1}H NMR spectrum (500.1 MHz) of **3a** in toluene-d_8 at −40 ºC.

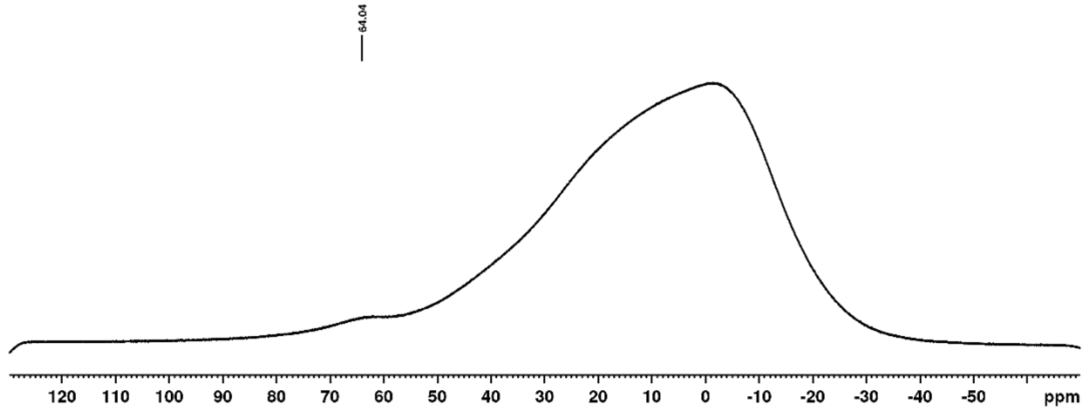

Figure S6. ^{11}B NMR spectrum (160.5 MHz) of **3a** in toluene-d_8 at 0 ºC.

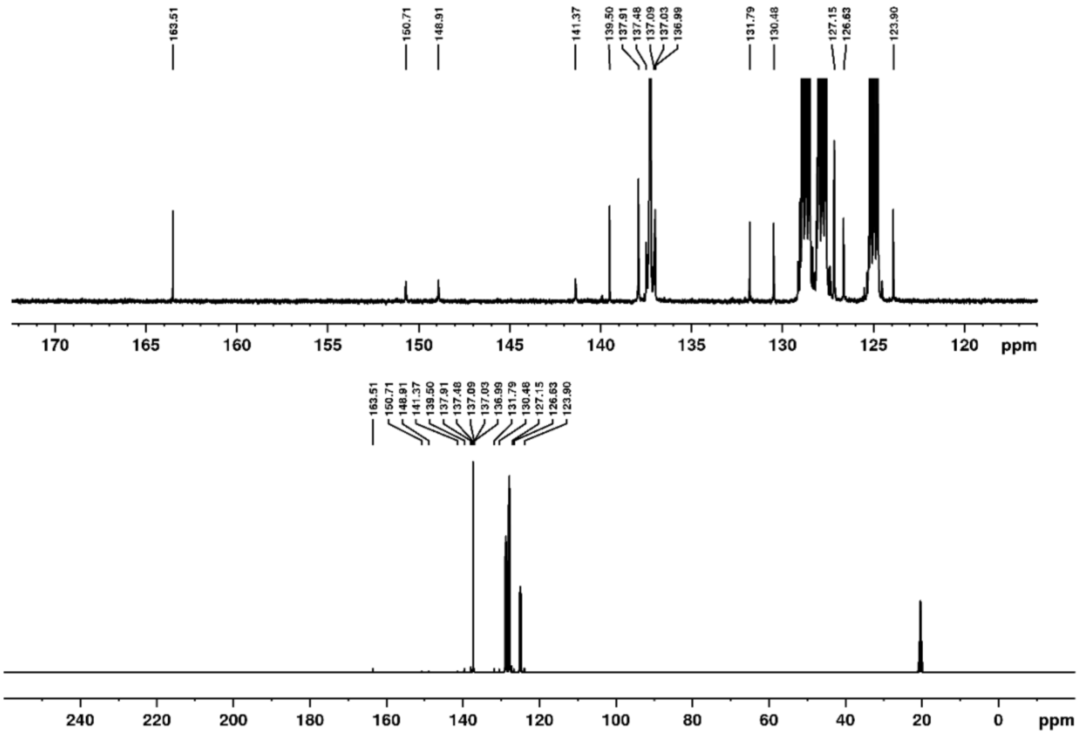

Figure S7. $^{13}C\{^{1}H\}$ NMR spectrum (125.8 MHz) of **3a** in toluene-d_8 at −40 ºC.

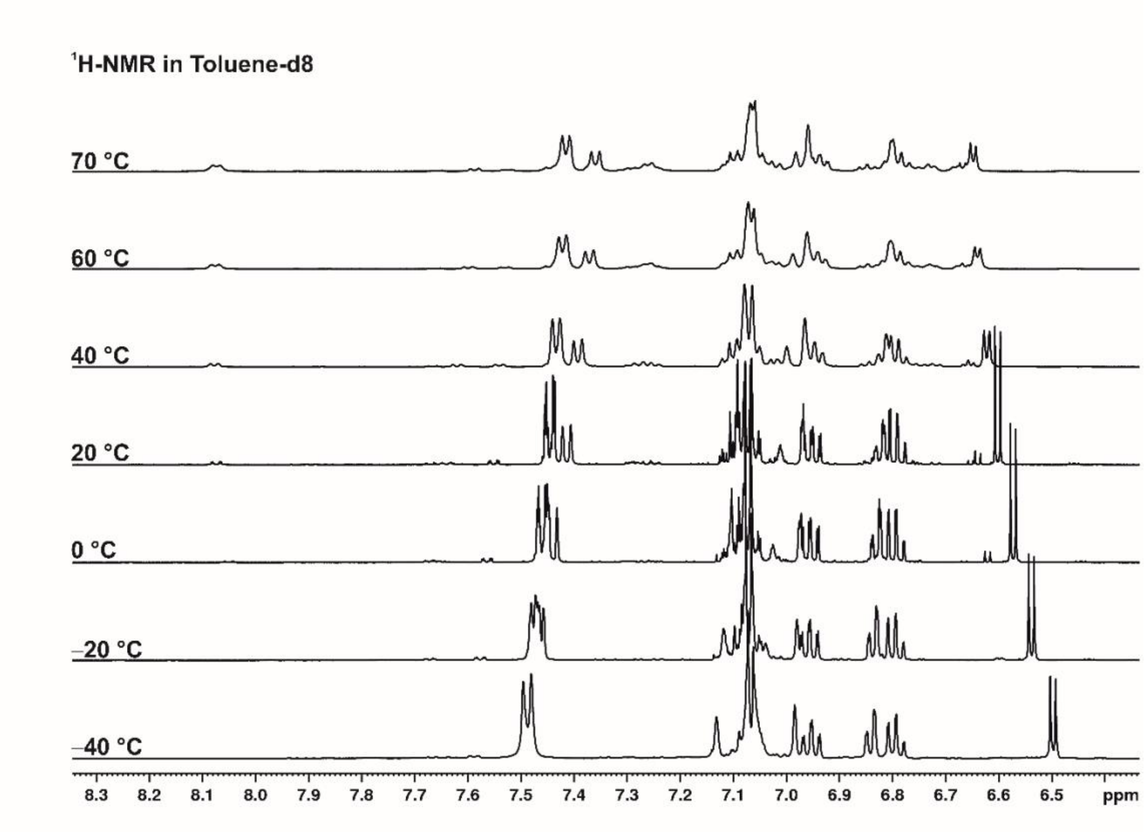

Figure S8. Variable temperature ^{1}H NMR spectra of **3a** in toluene-d_8.

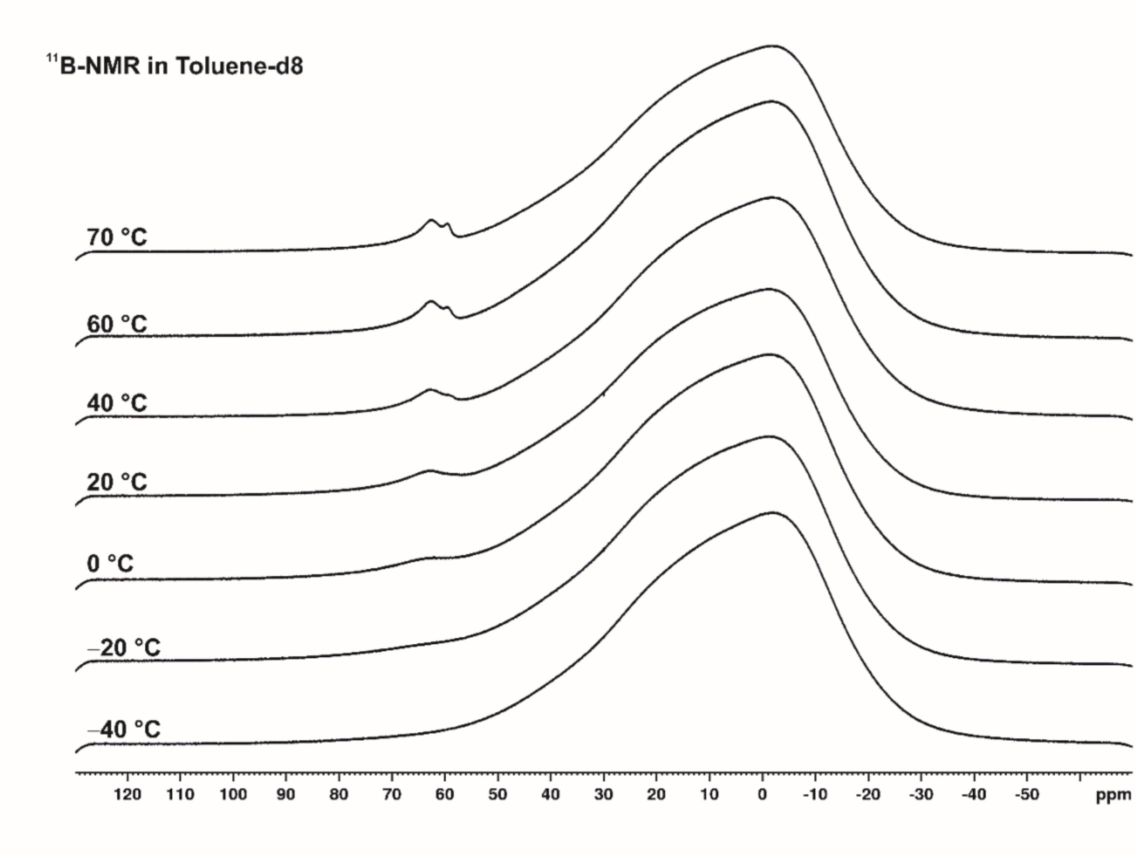

Figure S9. Variable-temperature ^{11}B NMR spectra of **3a** in toluene-d_8.

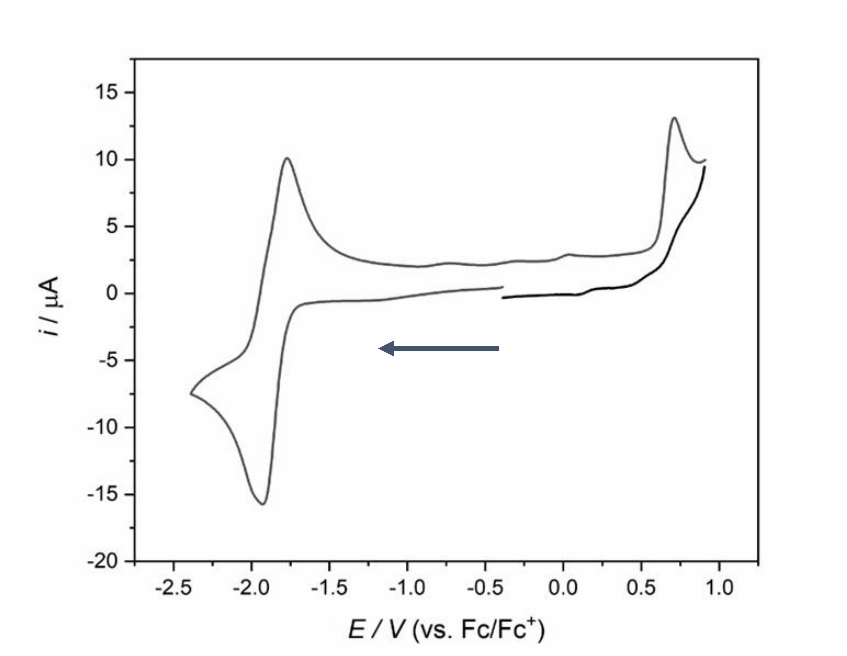

Figure S10. Cyclic voltammogram of **3a** in a 0.1 M [*n*Bu_4N][PF_6]/1,2-difluorobenzene solution with a scan rate of 250 mV/s.

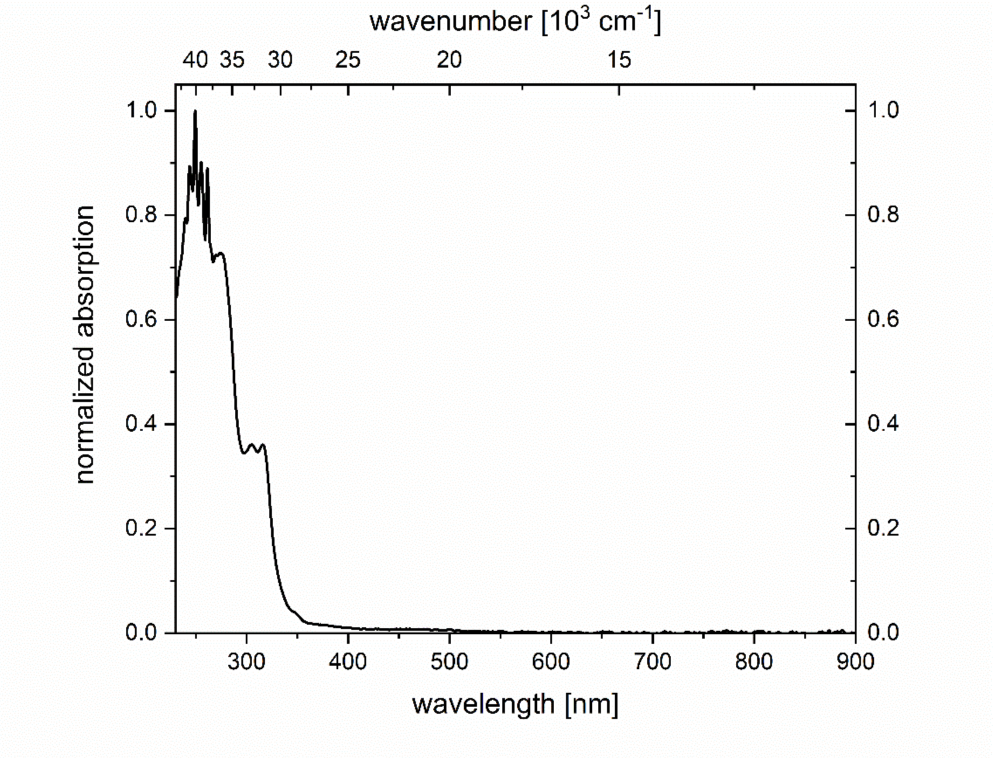

Figure S11. UV-vis spectrum of **3a** in dichloromethane.

2.3 Compound 3b

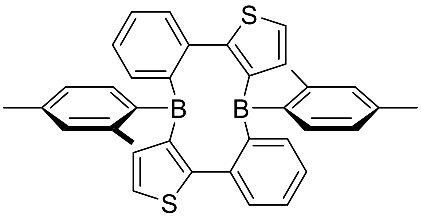

In a Schlenk tube, stannole **1** (300 mg, 977 μmol, 1.0 eq.) was dissolved in toluene (5 mL). At –78 °C, a solution of dibromo(xylyl)borane (270 mg, 977 μmol, 1.0 eq.) in toluene (5 mL) was added over a period of 30 min. Stirring was continued and the reaction mixture allowed to warm to room temperature. All volatiles were removed under reduced pressure and the remaining solid was washed three times with hexane. Compound **3b** (100 mg, 182 mmol) was isolated as an off-white solid in 37% yield.

Elemental analysis: Calculated: C: 78.85%, H: 5.51%, S: 11.69%; found: C: 77.69%, H: 5.44%, S: 11.56%.

HRMS (LIFDI, m/z): $[C_{36}H_{30}B_2S_2]^+$ calculated: 548.2970, found: 548.1966; $[C_{18}H_{15}BS]^+$ calculated: 274.0982, found: 274.0979.

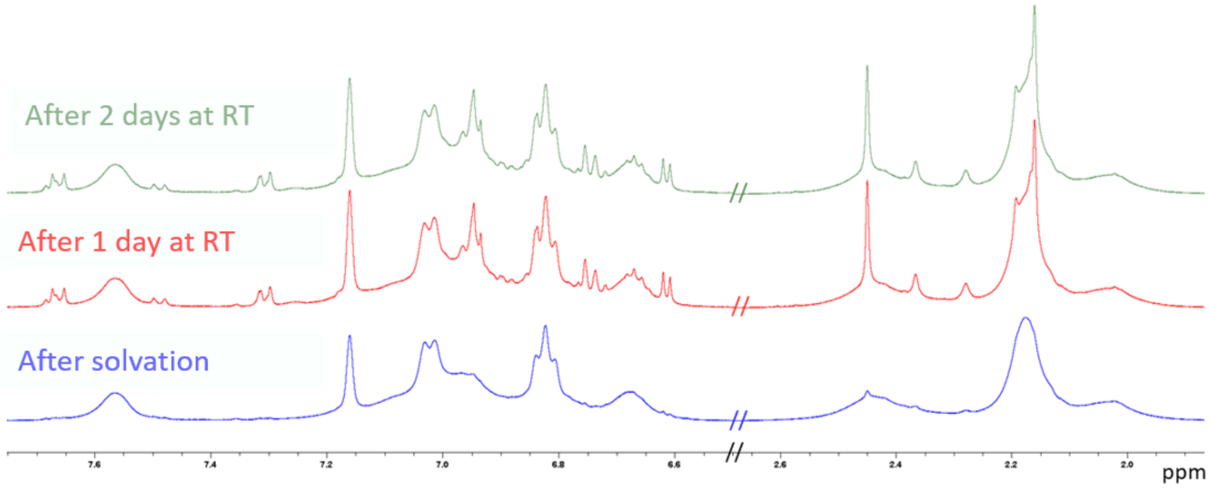

Figure S12. Section of the ^{1}H NMR spectrum of **3b** immediately after dissolution in benzene solution (blue), after one (red) and two days at room temperature (green).

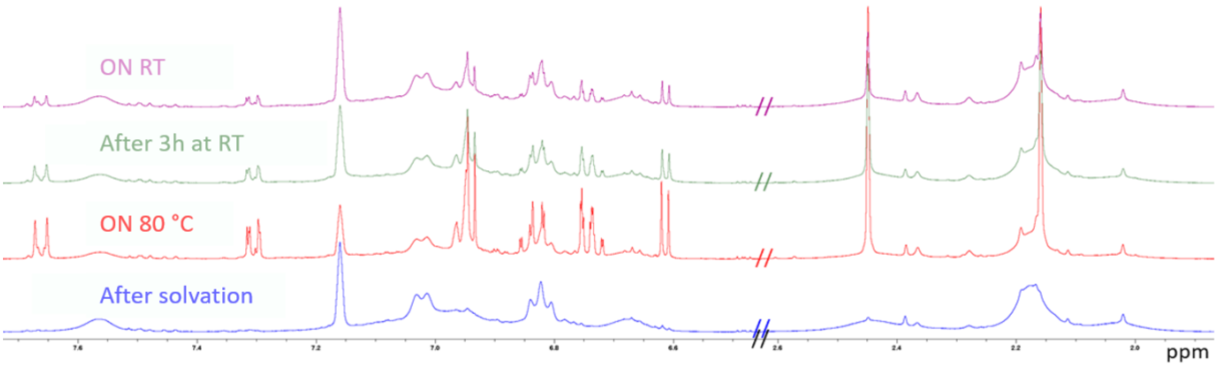

Figure S13. Section of the ^{1}H NMR spectrum of **3b** in benzene immediately after dissolution in benzene (blue), after heating overnight (ON) at 80 °C (red), after 3 h at room temperature (green), and after standing overnight (ON) at room temperature (purple).

2.4 Reactivity studies of 3a with Lewis bases

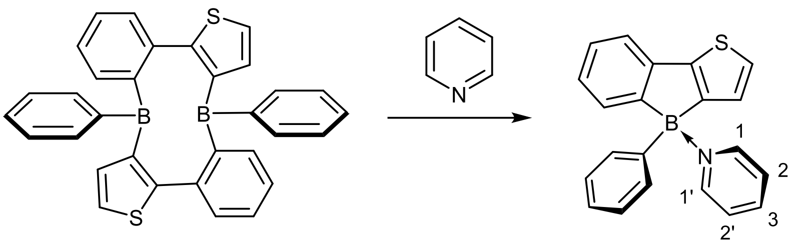

Scheme S1. Lewis acid-base adduct (**2a-pyr**) from addition of pyridine to **3a**.

Synthesis of 2a-pyr. To a J-Young NMR tube containing **3a** (30.0 mg, 60.9 µmol, 1.0 eq.) in benzene was added an excess of pyridine. After evaporation of all volatiles under reduced pressure, the crude product was washed with hexane and dried. Compound **2a-pyr** (70 mg, 215 µmol) was isolated in 27% yield.

^{1}H NMR (400 MHz, 298 K, CD_2Cl_2): δ(ppm) = 8.68 (d, $^{3}J_{HH}$ = 5.5 Hz, 2H, H-1/1‘), 7.96 (t, $^{3}J_{HH}$ =7.6 Hz, 1H, H-3), 7.52–7.37 (m, 3H, H-2/2‘, Ar-C*H*), 7.42 (d, $^{3}J_{HH}$ = 7.4 Hz, 2H, Ar-C*H*), 7.33 (d, $^{3}J_{HH}$ = 7.4 Hz, 1H, Ar-C*H*), 7.27 (d, $^{3}J_{HH}$ = 4.6 Hz, 1H, Ar-C*H*), 7.23–7.16 (m, 5H, Ar-C*H*), 7.07 (t, $^{3}J_{HH}$ = 7.3 Hz, 1H, Ar-C*H*). **^{11}B NMR** (128.5 MHz, 298 K, CD_2Cl_2): δ(ppm) = 1.1. **^{13}C{^{1}H} NMR** (100.7 MHz, 298 K, CD_2Cl_2): δ(ppm) = 151.9 (Ar-C_q), 146.2 (Ar-*C*H), 145.7 (Ar-C_q), 141.2 (Ar-*C*H), 133.5 (Ar-*C*H), 130.6 (Ar-*C*H), 128.7 (Ar-*C*H), 128.7 (Ar-C_q), 128.0 (Ar-*C*H), 127.3 (Ar-*C*H), 126.3 (Ar-*C*H), 126.2 (Ar-*CH*), 126.0 (Ar-*C*H), 125.2 (Ar-*C*H), 118.9 (Ar-*C*H). The quaternary carbon atoms connected to the boron center were not detected.

HRMS (LIFDI, m/z): $[C_{21}H_{16}BSN]^+$ calculated: 325.1091; found: 325.1088.

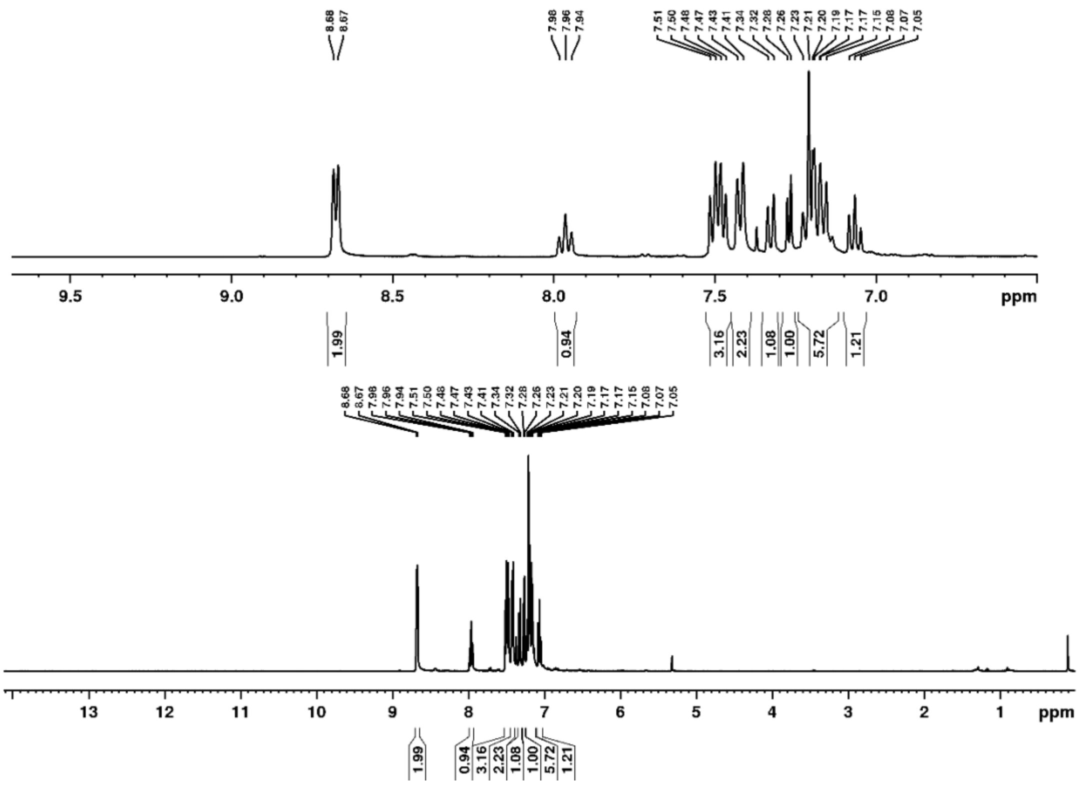

Figure S14. ^{1}H NMR spectrum (400 MHz) of **2a-pyr** in CD_2Cl_2.

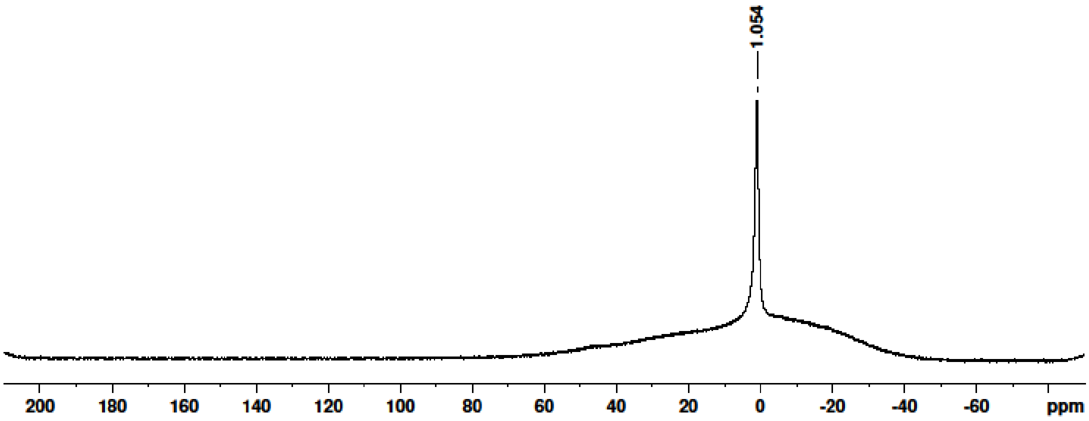

Figure S15. ^{11}B NMR spectrum (128.5 MHz) of **2a-pyr** in CD_2Cl_2.

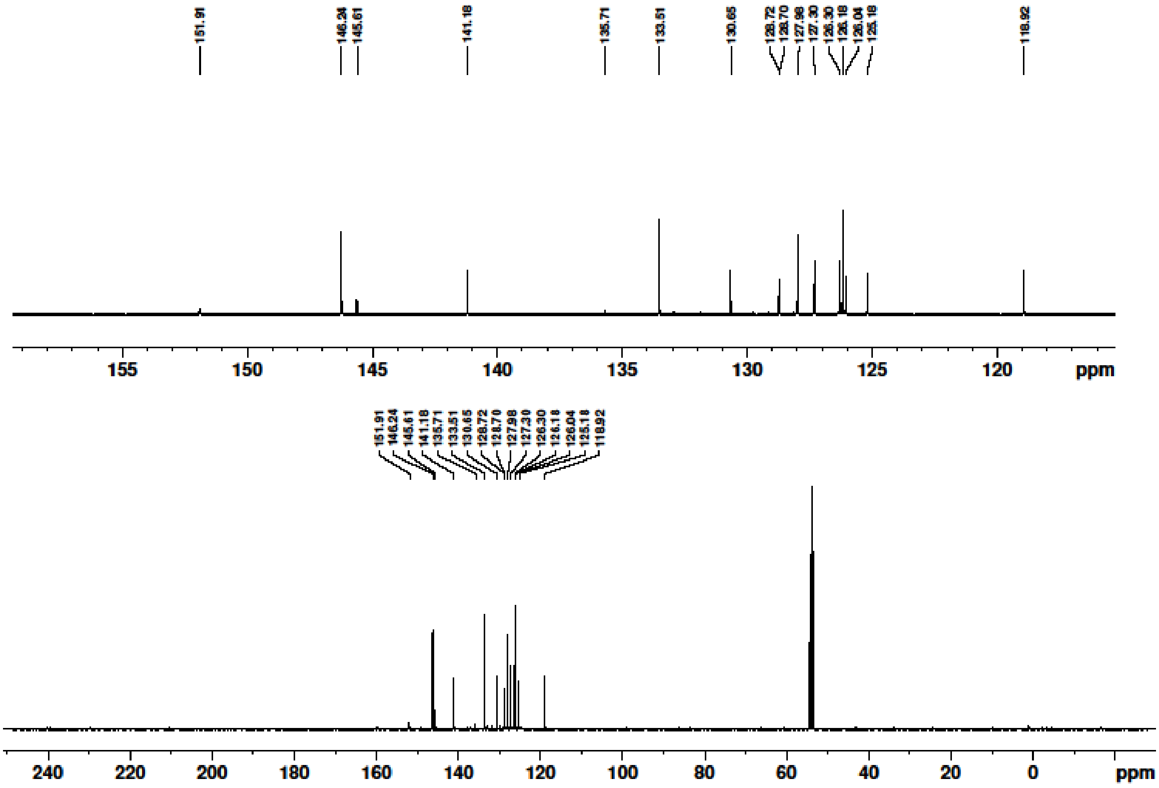

Figure S16. $^{13}C\{^{1}H\}$ NMR spectrum (100.7 MHz) of **2a-pyr** in CD_2Cl_2.

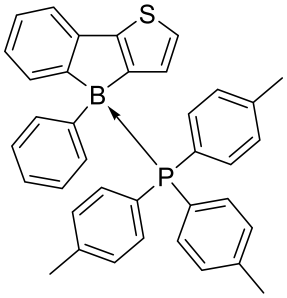

Synthesis of 2a-P. In a J-Young NMR tube, **3a** (30.0 mg, 61 µmol, 1.0 eq.) and tris(*p*-tolyl)phosphine (37.1 mg, 122 mmol, 2.0 eq.) were dissolved in benzene. After evaporation of all volatiles, the remaining solid was washed with hexane and dried under vacuum. Compound **2a-P** was isolated in 61% yield (20.6 mg, 37.4 mmol)

^{1}H NMR (500.1 MHz, C_6D_6): δ (ppm) = 7.85 (d, 2H, $^3J_{HH}$ = 7.4 Hz, Ar-*H*), 7.77(d, 1H, $^3J_{HH}$ = 7.1 Hz, Ar-*H*), 7.56 (d, 1H, $^3J_{HH}$ = 7.3 Hz, Ar-*H*) 7.27-7.18 (m, 11, 7.11 (m, 2H, Ar-*H*), 7.76 (dd, 6H, $^3J_{HH}$ = 8.0 Hz, $^4J_{HH}$ = 2.1 Hz, Ar-*H*), 1.88 (s, 9H, C*H*$_3$). **^{11}B NMR** (160.5 MHz, C_6D_6): δ (ppm) = -10.0 (s). **^{13}C{^{1}H} NMR** (125.8 MHz, C_6D_6): δ(ppm) = 152.8 (s, Ar-*C*$_q$), 142.8 (s, Ar-*C*H), 141.8 (d, J_{CP} = 2.5 Hz, tol-*C*$_q$), 135.6 (d, J_{CP} = 7.0 Hz, Ar-*C*H), 134.4 (d, J_{CP} = 8.7 Hz, tol-*C*H), 133.0 (s, Ar-*C*H), 130.6 (s, Ar-*C*H), 129.5 (d, J_{CP} = 9.9 Hz, tol-*C*H), 127.7 (s, Ar-*C*H), 127.1 (s, Ar-*C*H), 126.1 (s, Ar-*C*H), 125.3(s, Ar-*C*H), 124.6 (s, Ar-*C*H), 124.6 (d, J_{CP} = 53.8 Hz, tol-*C*q), 119.4(s, Ar-*C*H), 112.6 (m, Ar-*C*$_q$), 21.2 (d, J_{CP} = 1.1 Hz, tol-*C*H$_3$). **^{31}P NMR** (202.5 MHz, C_6D_6): δ (ppm) = 6.5(s).

HRMS (LIFDI, m/z): $[C_{36}H_{32}BS_2P]^+$ calculated: 570.1771; found: 570.1769.

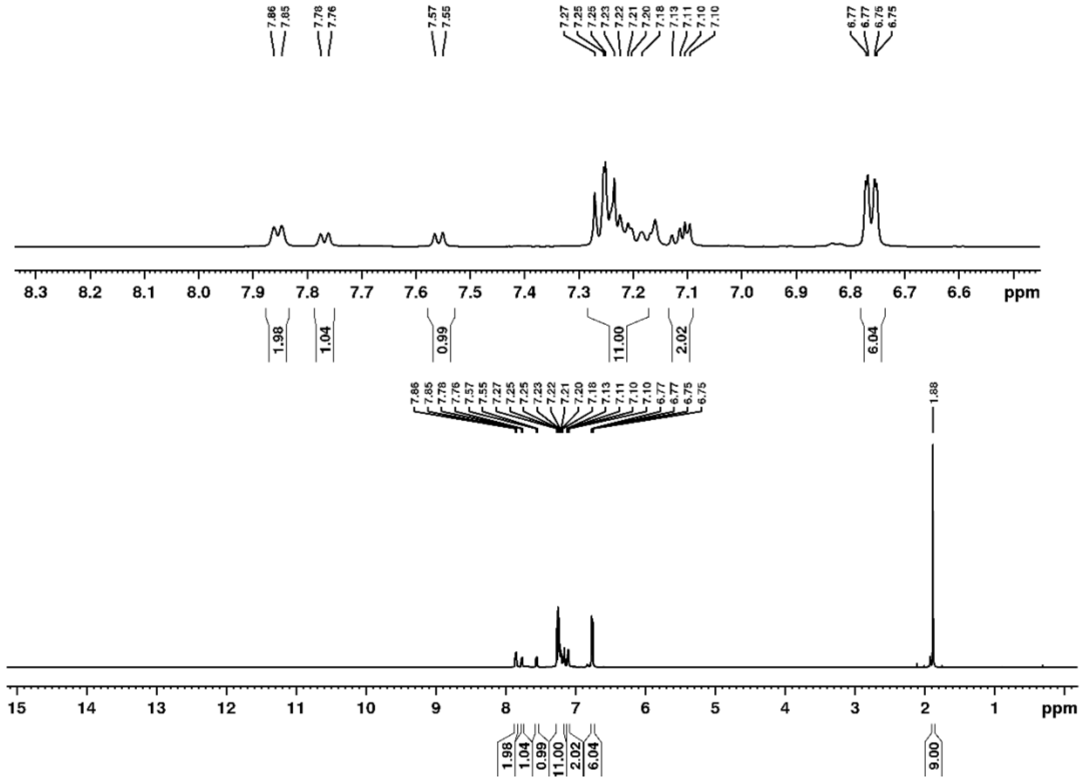

Figure S17. ^{1}H NMR spectrum (500.1 MHz) of **2a-P** in C_6D_6.

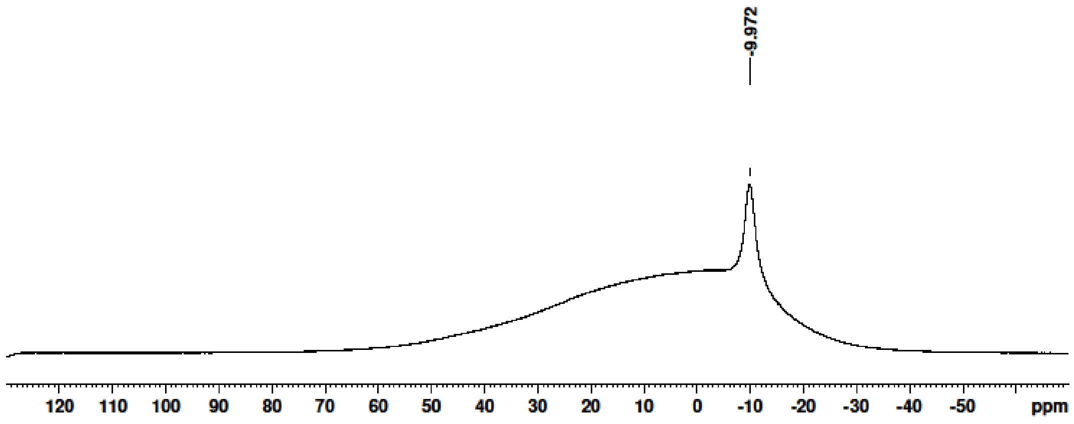

Figure S18. ^{11}B NMR spectrum (160.5 MHz) of **2a-P** in C_6D_6.

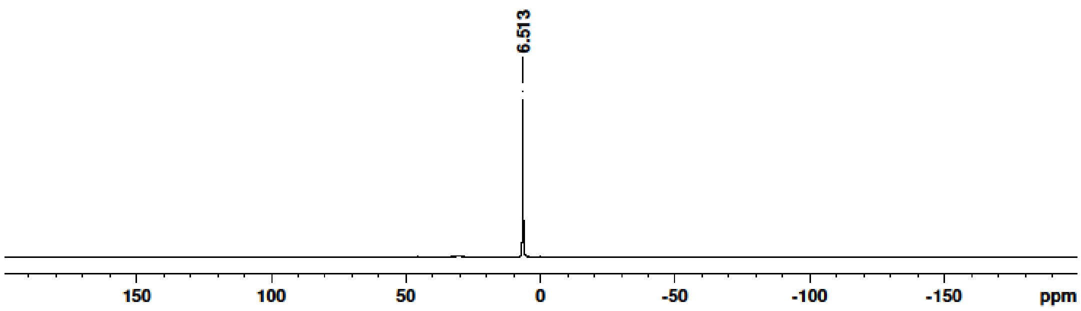

Figure S19. ^{31}P NMR spectrum (202.5 MHz) of **2a-P** in C_6D_6.

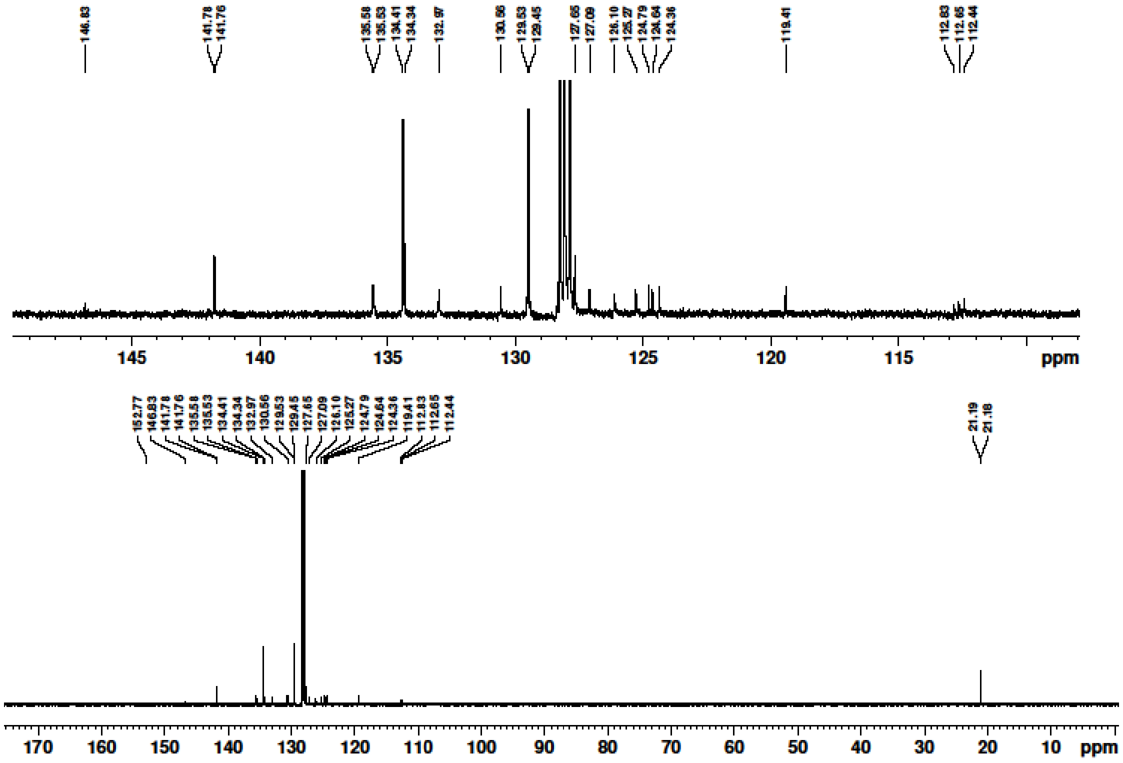

Figure S20. ^{13}C NMR spectrum (125.8 MHz) of **2a-P** in C_6D_6.

2.5 Compound 4a

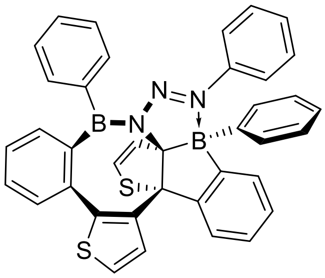

To a toluene solution of **3a** (60 mg, 122 μmol, 1.0 eq.) was added phenyl azide (14.5 mg, 122 μmol, 1.0 eq.). The reaction mixture was stirred for 10 min before all volatiles were removed under reduced pressure. The remaining solid was washed three times with 10 mL of hexane. Compound **4a** (50 mg, 81.8 μmol, 67% yield) was isolated as an orange solid.

^{1}H NMR (500.1 MHz, CD_2Cl_2): δ (ppm) = 7.70-7.68 (m, 1H, Ar*H*), 7.54-7.51 (m, 5H, Ar*H*), 7.51-7.49 (m, 2H, Ar*H*), 7.48-7.46 (m, 2H, Ar*H*), 7.41-7.38(m, 2H, Ar*H*), 7.37-7.38 (m, 8H, Ar*H*), 7.26-7.22(m, 3H, Ar*H*), 7.17-7.15(m, 1H, Ar*H*), 6.97 (d, 1H, $^{3}J_{HH}$ = 5.3 Hz, thiophene-*H*), 6.06 (d, 1H, $^{3}J_{HH}$ = 5.3 Hz, thiophene-*H*), 5.57 (d, 1H, $^{3}J_{HH}$ = 6.1 Hz, thiophene-*H*), 4.52 (d, 1H, $^{3}J_{HH}$ = 6.1 Hz, thiophene-*H*). **^{11}B NMR** (160.5 MHz, CD_2Cl_2): δ (ppm) = 52.2 (br, s), 7.25 (s). **^{13}C{^{1}H} NMR** (125.8 MHz, CD_2Cl_2): δ (ppm) = 158.1 (Ar-C_q), 143.1 (Ar-C_q), 141.1 (Ar-C_q), 139.7 (Ar-C_q), 138.8 (Ar-C_q), 138.7 (Ar-*C*H), 134.9 (Ar-*C*H), 134.4 (Ar-*C*H), 132.7 (Ar-*C*H), 132.6 (Ar-*C*H), 130.3 (Ar-*C*H), 130.2 (Ar-*C*H), 129.0 (Ar-*C*H), 129.6 (Ar-*C*H), 129.2 (Ar-*C*H), 128.7 (Ar-*C*H), 128.2 (Ar-*C*H), 127.9 (Ar-*C*H), 127.5 (Ar-*C*H), 127.1 (Ar-*C*H), 126.7 (Ar-*C*H), 126.5 (Ar-*C*H), 122.7 (Ar-*C*H), 122.5 (Ar-*C*H), 121.8 (Ar-*C*H), 78.1 (Ar-C_q). The quaternary carbon atoms connected to the boron center were not detected.

HRMS (LIFDI, m/z): $[C_{38}H_{27}B_2N_3S_3]^+$ calculated: 611.1827; found: 611.1825.

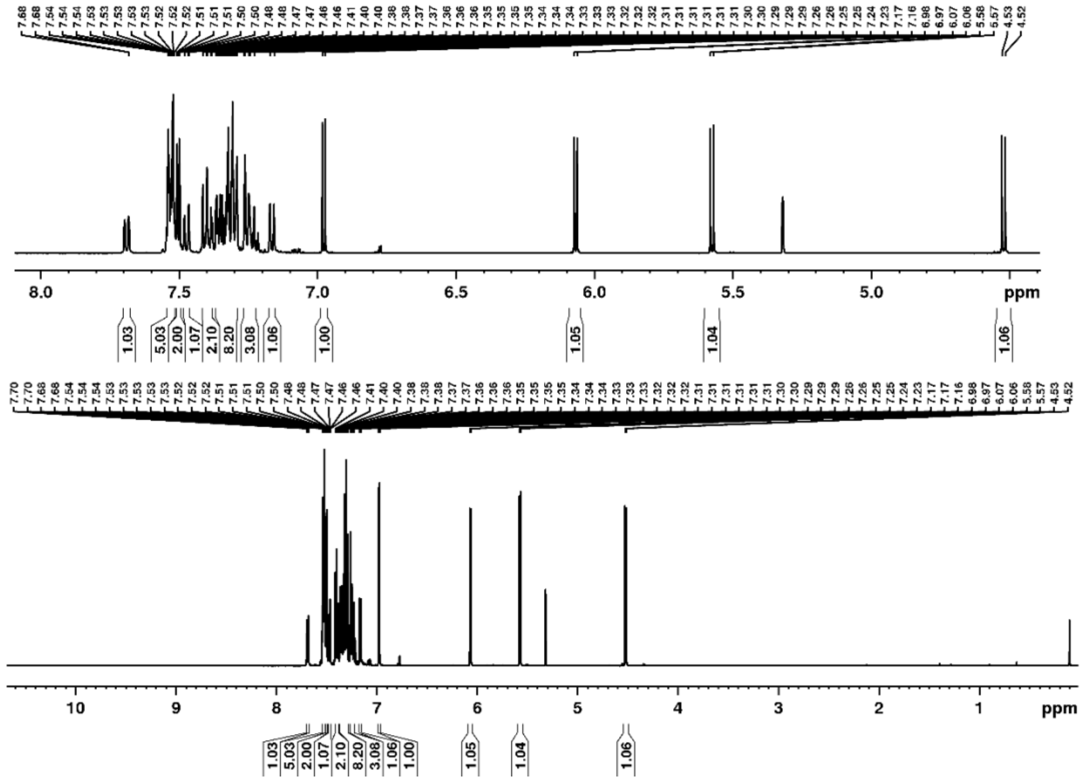

Figure S21. ^{1}H NMR spectra (500.1 MHz) of **4a** in CD_2Cl_2.

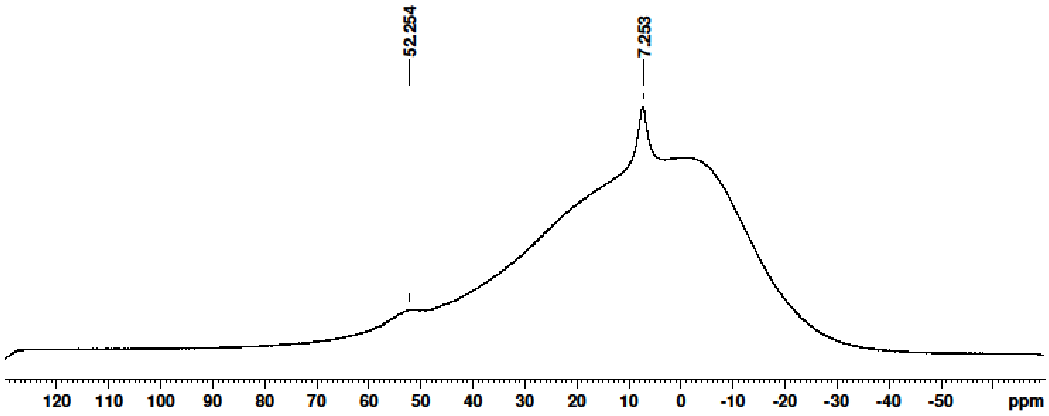

Figure S22. ^{11}B NMR spectrum (160.5 MHz) of **4a** in CD_2Cl_2.

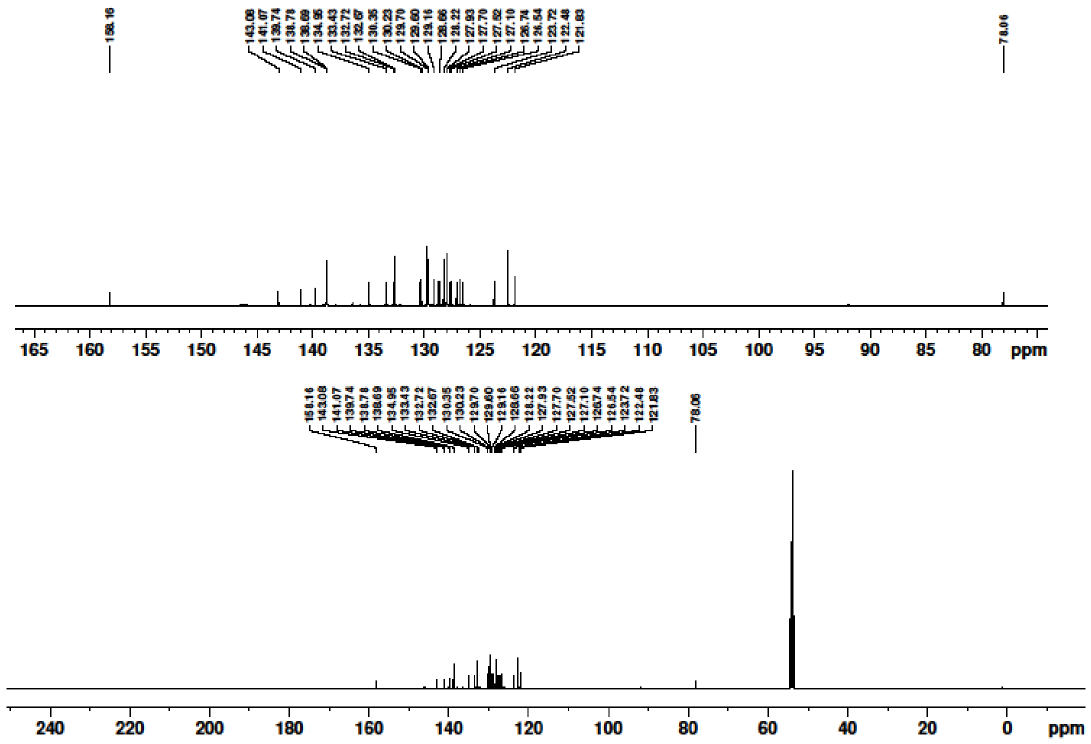

Figure S23. $^{13}C\{^{1}H\}$ NMR spectrum (125.8 MHz) of **4a** in CD_2Cl_2.

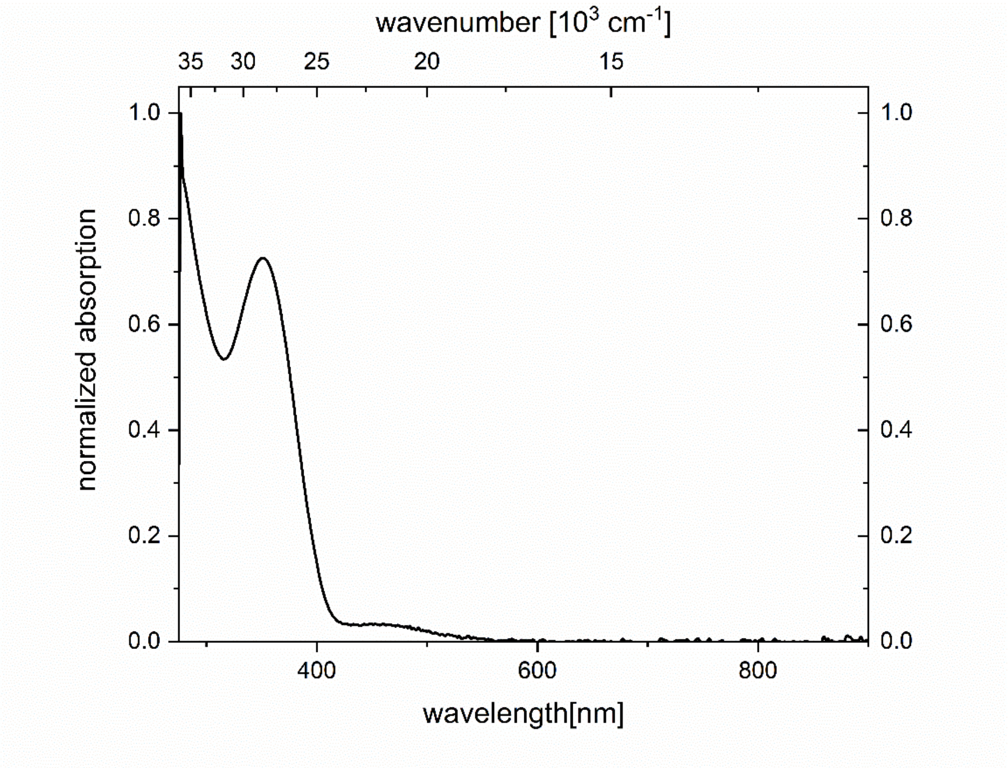

Figure S24. UV-vis spectrum of **4a** in benzene. Absorption maxima at 349 nm and 467 nm.

2.6 Compound 4b

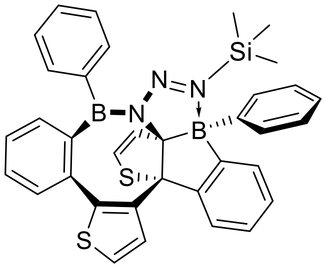

To a toluene solution of **3a** (100 mg, 203 µmol, 1.0 eq.) was added trimethylsilyl azide (23.4 mg, 203 µmol, 1.0 eq.) and the solution was stirred for 12 h. All volatiles were removed under reduced pressure and the remaining solid washed three times with 10 mL of hexane. Compound **4a** (40 mg, 65.9 µmol) was isolated as an orange solid in 33% yield.

^{1}H NMR (500.1 MHz, C_6D_6): δ(ppm) = 7.75-7.73 (m, 1H, Ar-*H*), 7.67-7.63 (m, 3H, Ar-*H*), 7.57 (d, 1H , $^{3}J_{HH}$ = 7.7 Hz, Ar-*H*), 7.41-7.39 (m, 2H, Ar-*H*),m 7.33 (t, 2H, $^{3}J_{HH}$ = 7.4 Hz, Ar-*H*), 7.29-7.27 (m, 2H, Ar-*H*), 7.23-7.18 (m, 3H, Ar-*H*), 7.16-7.12 (m, 3H, Ar-*H*), 7.05 (m, 2H, Ar-*H*), 6.57 (d, 1H, $^{3}J_{HH}$ = 5.3, thiophene-*H*), 6.26 (d, 1H, $^{3}J_{HH}$ = 5.3, thiophene-*H*), 5.32 (d, 1H, $^{3}J_{HH}$ = 6.1, thiophene-*H*), 4.62 (d, 1H, $^{3}J_{HH}$ = 6.1, thiophene-*H*), -0.04 (s, 9H, Si(C*H*$_3$)$_3$). **^{11}B NMR** (160.5 MHz, C_6D_6): δ(ppm) = 52.8 (br, s), 8.71 (s). **^{13}C{^{1}H} NMR** (125.8 MHz, C_6D_6): δ(ppm) = 158.4 (Ar-*C*$_q$), 143.2 (Ar-*C*$_q$), 140.6 (Ar-*C*$_q$), 140.0 (Ar-*C*$_q$), 138.7 (Ar-*C*H), 134.8 (Ar-*C*H), 133.0 (Ar-*C*H), 132.8 (Ar-*C*H), 132.2 (Ar-*C*H), 130.4 (Ar-*C*H), 129.6 (Ar-*C*H), 129.5 (Ar-*C*H), 128.4 (Ar-*C*H), 128.2 (Ar-*C*H), 127.6 (Ar-*C*H), 127.5 (Ar-*C*H), 127.3 (Ar-*C*H), 127.2 (Ar-*C*H), 126.6 (Ar-*C*H), 126.5 (Ar-*C*H), 123.2 (Ar-*C*H), 122.1 (Ar-*C*H), 78.1 (*C*$_q$), -0.8(Si(*C*H$_3$)$_3$. Due to broadening, the quaternary carbon atoms connected to the boron center were not detected.

HRMS (LIFDI, m/z): $[C_{35}H_{31}B_2N_3S_3Si]^+$ calculated: 607.1909; found: 607.1906

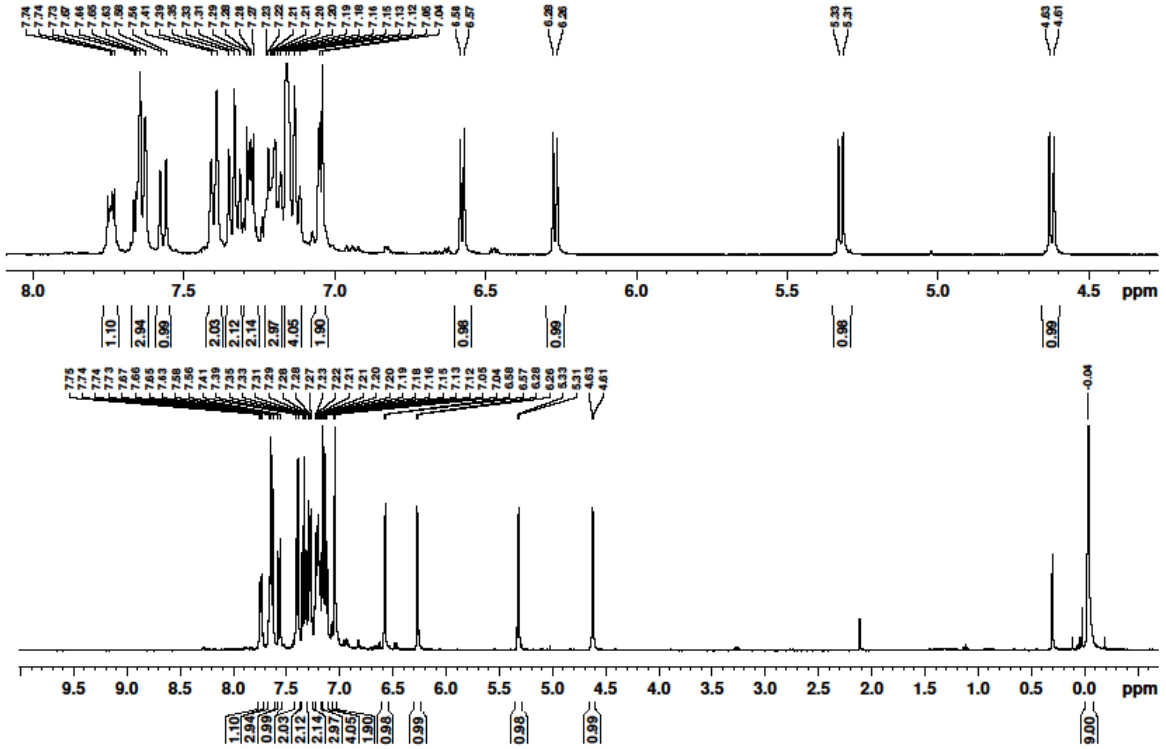

Figure S25. ^{1}H NMR spectrum (500.1 MHz) of **4b** in C_6D_6.

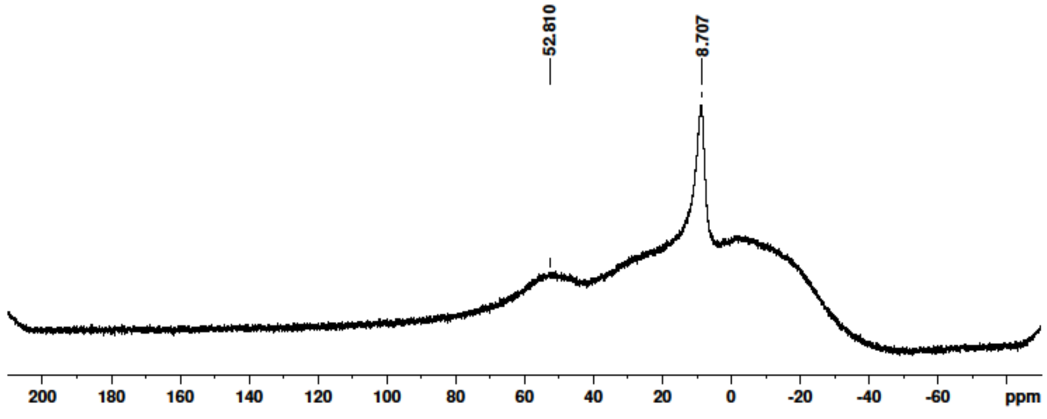

Figure S26. ^{11}B NMR spectrum (160.5 MHz) of **4b** in C_6D_6.

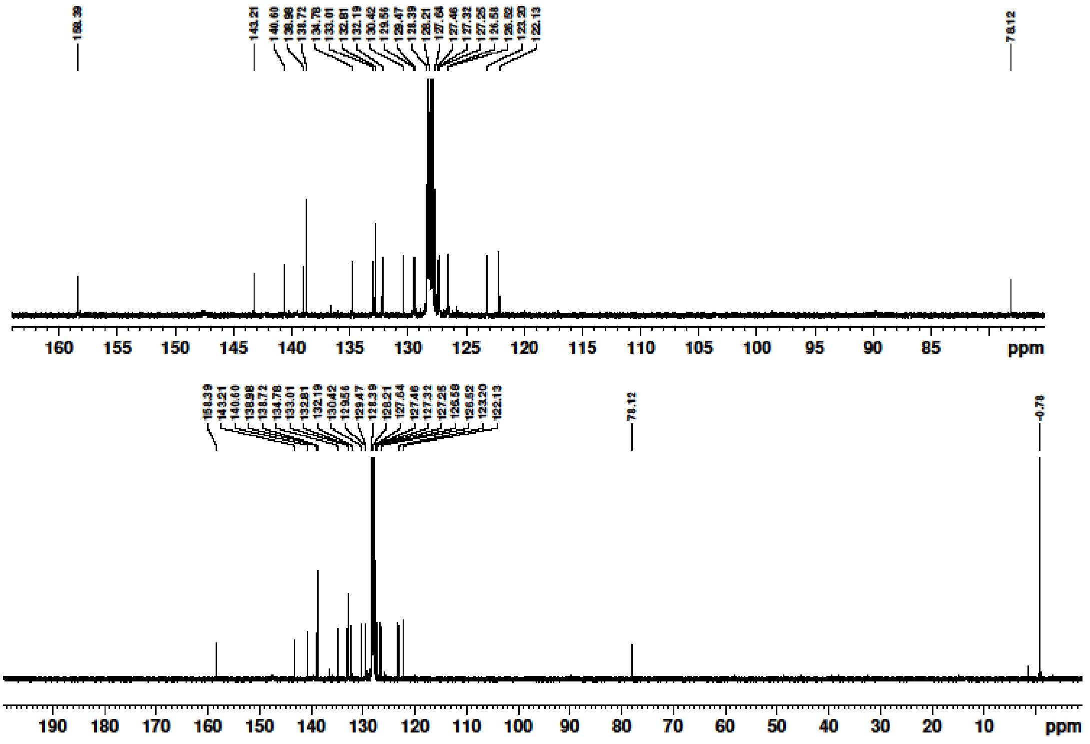

Figure S27. $^{13}C\{^1H\}$ NMR spectrum (125.8 MHz) of **4b** in C_6D_6.

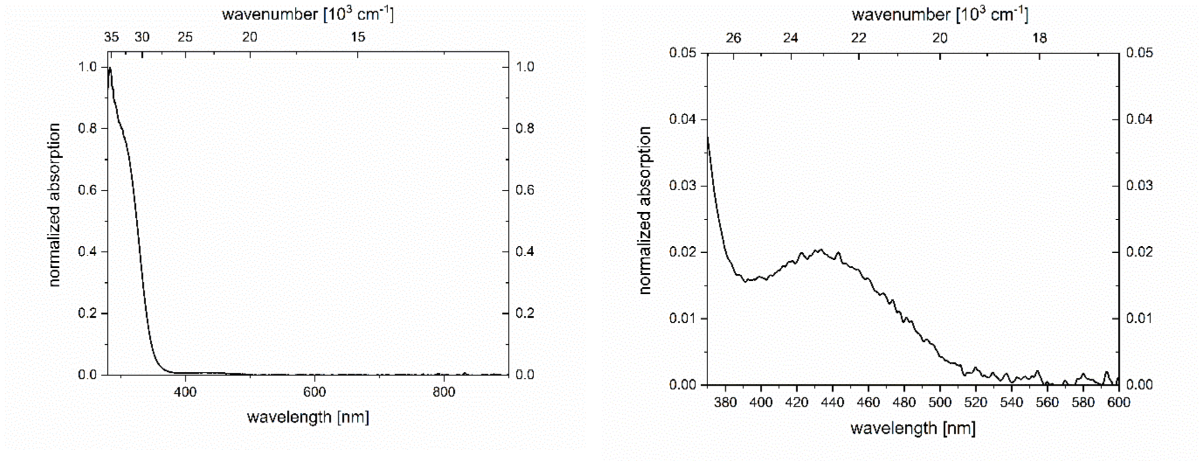

Figure S28. UV-vis spectra of **4b** in benzene. Lowest-energy absorption at 430 nm.

2.6 Compound 4c

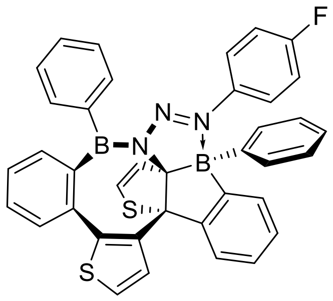

In a Young NMR tube, *p*-fluorophenyl azide (8.36 mg, 60.9 µmol, 1.0 eq.) was added to a toluene solution of **3a** (30 mg, 60.9 µmol, 1.0 eq.) and the solution shaken. All volatiles were removed under reduced pressure and the remaining solid washed three times with 10 mL of hexane. Compound **4c** (23.4 mg, 37.2 µmol) was isolated as an orange solid in 67% yield.

^{1}H NMR (500.1 MHz, C_6D_6): δ (ppm) = 7.76-7.74 (m, 1H, Ar-*H*), 7.67-7.63 (m, 2H, Ar-*H*), 7.63-7.62 (m, 1H, Ar-*H*), 7.57-7.55 (m, 1H, Ar-*H*), 7.50-7.48 (m, 2H, Ar-*H*), 7.35-7.31 (m, 4H, Ar-*H*), 7.29-7.17 (m, 5H, Ar-*H*), 7.14-7.03 (m, 4H, Ar-*H*), 6.51 (d, 1H, $^3J_{HH}$ = 5.3 Hz, thiophene-*H*), 6.29 (d, 1H, $^3J_{HH}$ = 8.1 Hz, Ar-C*H*), 6.27 (d, 1H, $^3J_{HH}$ = 8.1 Hz, Ar-C*H*), 6.16 (d, 1H, $^3J_{HH}$ = 5.3 Hz, thiophene-*H*), 5.37 (d, 1H, $^3J_{HH}$ = 6.1 Hz, thiophene-*H*), 4.6 (d, 1H, $^3J_{HH}$ = 6.1 Hz, thiophene-*H*). **^{11}B NMR** (160.5 MHz, C_6D_6): δ (ppm) = 53.9 (br, s), 7.53 (s). **^{13}C{^{1}H} NMR** (125.8 MHz, C_6D_6): δ (ppm) = 164.3 (Ar-*C*q), 162.3 (Ar-*C*q), 158.4 (Ar-*C*q), 146.6 (Ar-*Cq*-B), 146.1 (Ar-*Cq*-B), 143.2 (Ar-*C*q), 140.2 (Ar-*C*q), 140.1 (Ar-*Cq*-B), 139.5 (Ar-*Cq*-B), 139.1 (Ar-*C*q), 138.7 (Ar-*C*H), 137.1 (Ar-*C*q), 137.0 (Ar-*C*H), 135.2 (Ar-*C*H), 133.1 (Ar-*C*H), 132.8 (Ar-*C*H), 132.6 (Ar-*C*H), 130.4 (Ar-*C*H), 129.8 (Ar-*C*H), 129.4 (Ar-*C*H), 128.8 (Ar-*C*H), 128.5 (Ar-*C*H), 128.3 (Ar-*C*H), 127.9 (Ar-*C*H), 127.4 (Ar-*C*H), 126.9 (Ar-*C*H), 126.8 (Ar-*C*H), 124.4 (Ar-*C*H), 123.6 (Ar-*C*H), 121.3 (Ar-*C*H), 166.5 (Ar-*C*H), 166.3 (Ar-*C*H), 92.4(*Cq*-B), 78,2 (*Cq*). **^{19}F NMR** (470.6 MHz, C_6D_6): δ (ppm) = -110.0.

HRMS (LIFDI): $[C_{38}H_{26}B_2N_3S_3F]^+$ calculated: 629.1733 m/z; found: 629.1729 m/z.

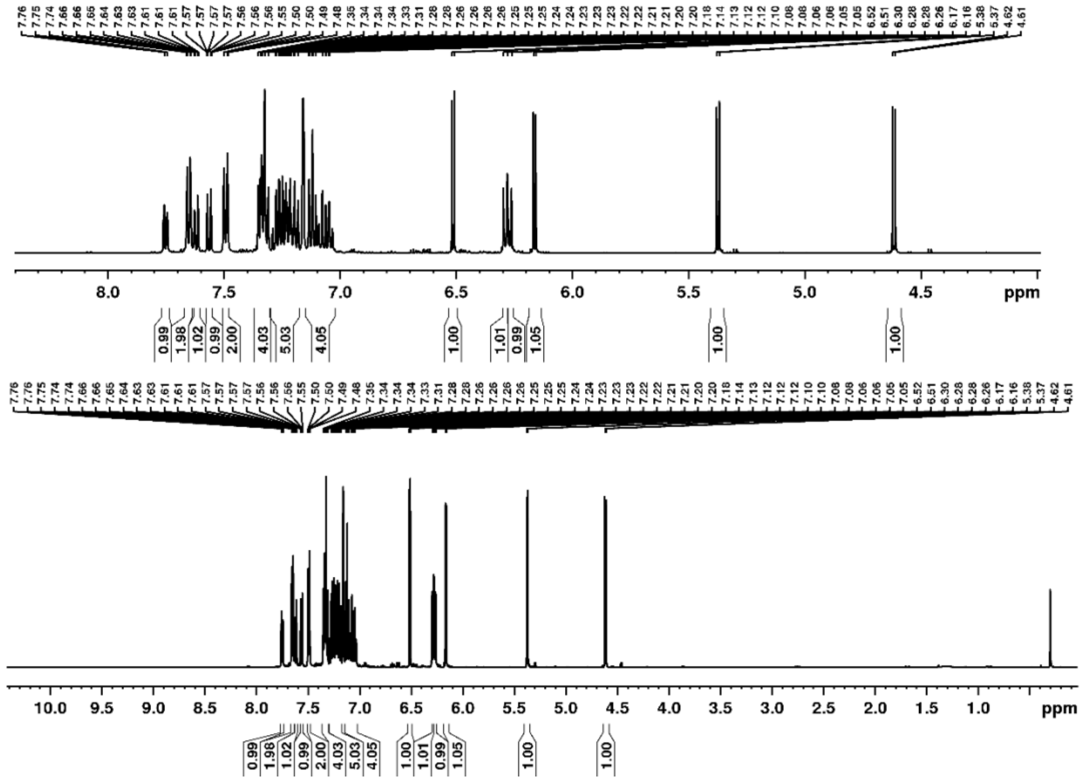

Figure S29. ^{1}H NMR spectrum (500.1 MHz) of **4c** in C_6D_6.

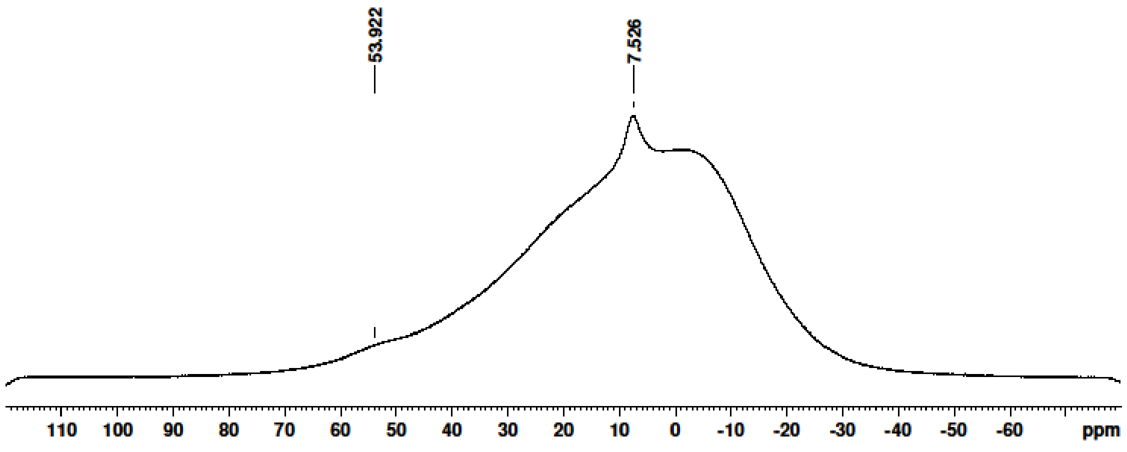

Figure S30. ^{11}B NMR spectrum (160.5 MHz) of **4c** in C_6D_6.

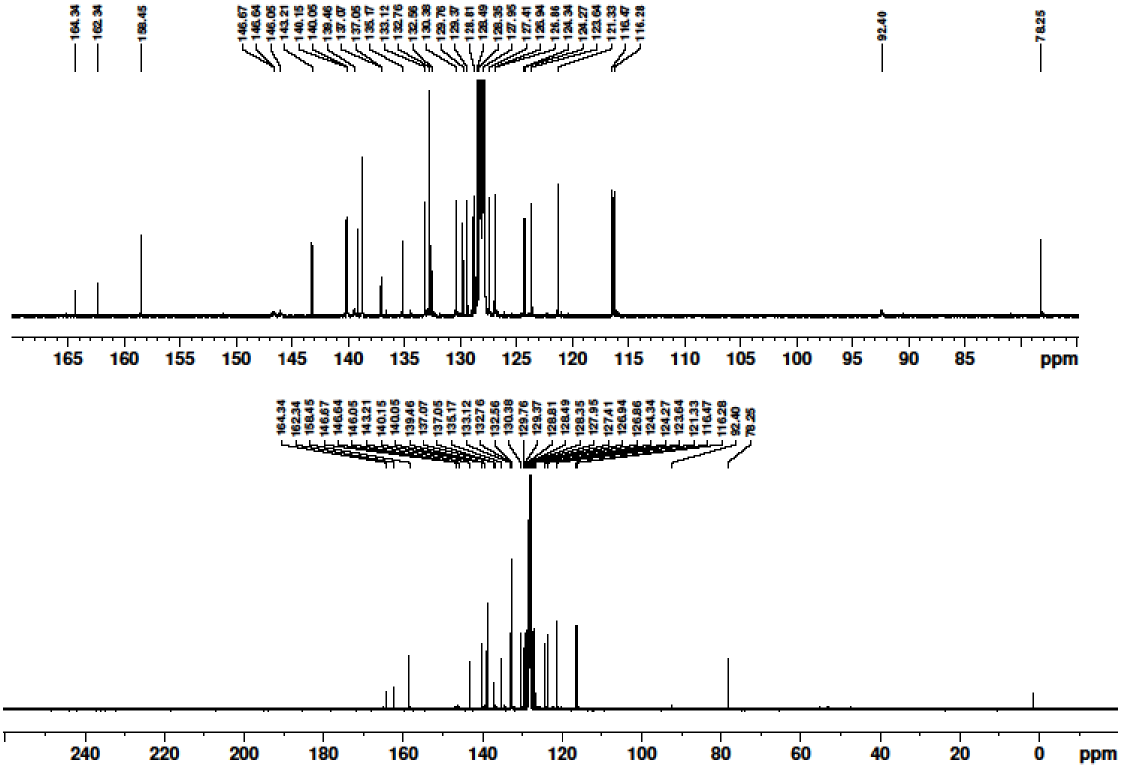

Figure S31. $^{13}C\{^{1}H\}$ NMR spectrum (125.8 MHz) of **4c** in C_6D_6.

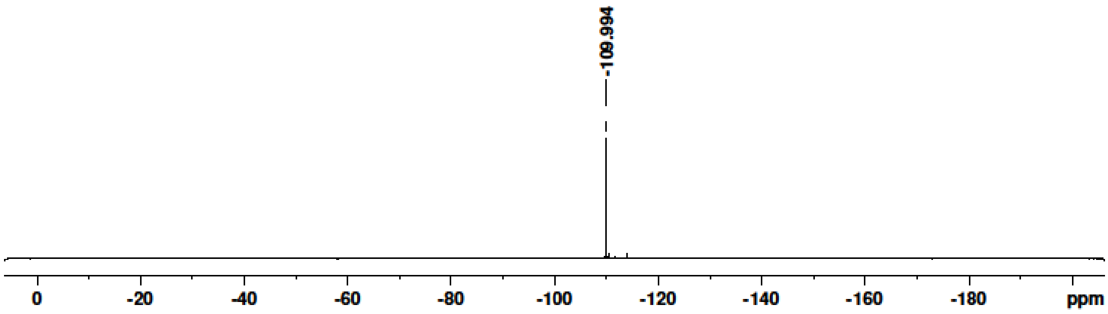

Figure S32. ^{19}F NMR spectrum (470.6 MHz) of **4c** in C_6D_6.

2.6 Compound 4d

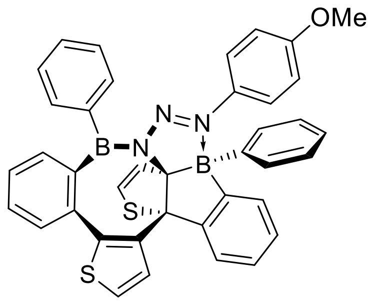

In a Young NMR tube, *p*-methoxyphenyl azide (9.54 mg, 60.9 µmol, 1.0 eq.) was added to a toluene solution of **3a** (30 mg, 60.9 µmol, 1.0 Äq.) and the solution shaken. All volatiles were removed under reduced pressure and the remaining solid was washed three times with 10 mL of hexane. Compound **4d** (21.0 mg, 32.7 µmol) was isolated as an orange solid in 54% yield.

^{1}H NMR (500 MHz, 298 K, C_6D_6): δ(ppm) = 7.89 (ddd, ^{3}J = 7.3 Hz, ^{4}J = 1.3 Hz, ^{5}J = 0.5 Hz, 1H, Ar-C*H*), 7.70 (ddd, ^{3}J = 7.8 Hz, ^{4}J = 2.4 Hz, ^{5}J = 1.2 Hz, 2H, Ar-C*H*), 7.65 (ddd, ^{3}J = 7.5 Hz, ^{4}J = 1.1 Hz, ^{5}J = 0.6 Hz, 1H, Ar-C*H*), 7.59–7.56 (m, 3H, Ar-C*H*), 7.53–7.49 (m, 2H, Ar-C*H*), 7.36–7.33 (m, 2H., Ar-C*H*), 7.32–7.18 (m, 7H, Ar-C*H*), 7.15–7.05 (m, 4H, Ar-C*H*), 6.51 (d, ^{3}J = 5.2 Hz, 1H, S-C*H*), 6.25–6.22 (m, 2H, Ar-C*H*), 6.22 (d, ^{3}J = 5.2 Hz, 1H, S-CH-C*H*), 5.39 (d, ^{3}J = 6.3 Hz, 1H, S-C*H*), 4.66 (d, ^{3}J = 6.3 Hz, 1H, S-CH-C*H*), 3.00 (s, 3H, OCH_3). **^{11}B NMR** (160 MHz, 298 K, C_6D_6): δ(ppm) = 53.2, 7.85. **^{13}C{^{1}H} NMR** (126 MHz, 298 K, C_6D_6): δ(ppm) = 161.3 (Ar-C_q), 158.6 (Ar-C_q), 147.2 (B-C_q), 146.5 (B-C_q), 143.3 (Ar-C_q), 140.6 (B-C_q), 140.4 (Ar-C_q), 139.6 (B-C_q), 139.1 (Ar-C_q), 138.7 (Ar-*C*H), 134.9 (Ar-*C*H), 134.2 (Ar-C_q), 133.3 (Ar-*C*H), 132.9 (Ar-*C*H), 132.2 (Ar-*C*H), 130.4 (Ar-*C*H), 129.5 (Ar-*C*H), 128.7 (Ar-*C*H), 128.4 (Ar-*C*H), 127.9 (Ar-*C*H), 127.7 (Ar-*C*H), 127.5 (Ar-*C*H), 127.3 (Ar-*C*H), 127.0 (Ar-*C*H), 126.8 (Ar-*C*H), 124.1 (Ar-*C*H), 123.6 (Ar-*C*H), 122.0 (Ar-*C*H), 114.7 (Ar-*C*H), 91.8(B-C_q), 78.2 (C_q), 54.9 (OCH_3),

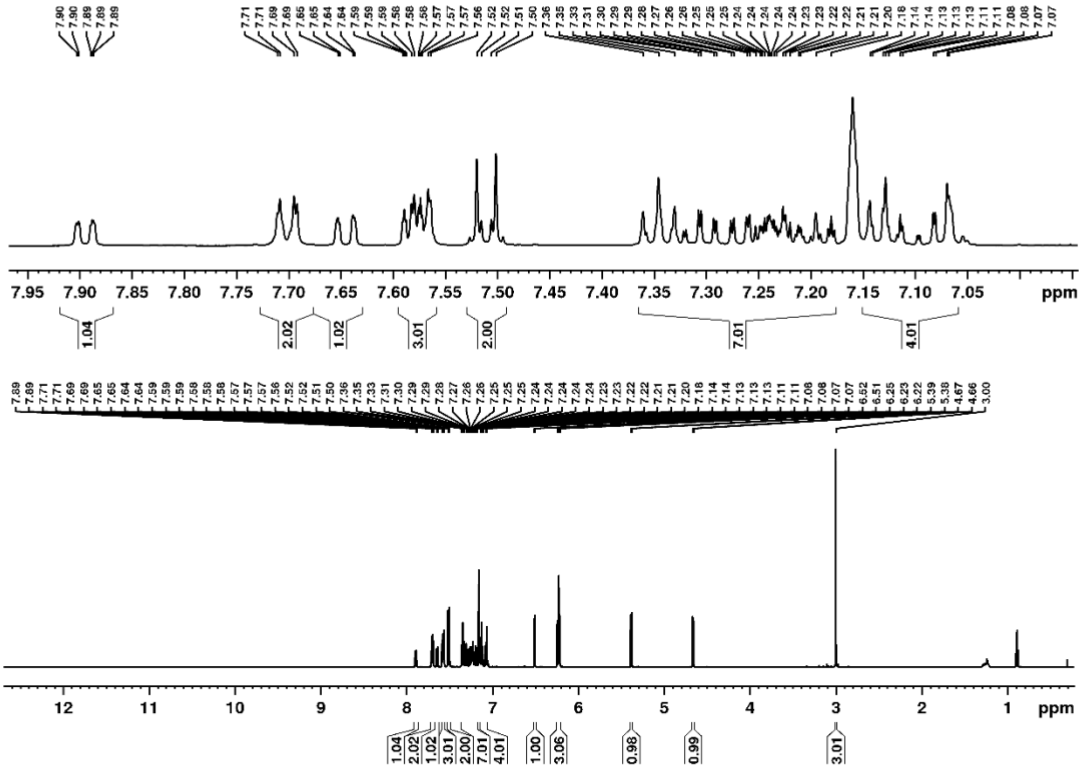

Figure S33. ^{1}H NMR spectrum (500.1 MHz) of **4d** in C_6D_6.

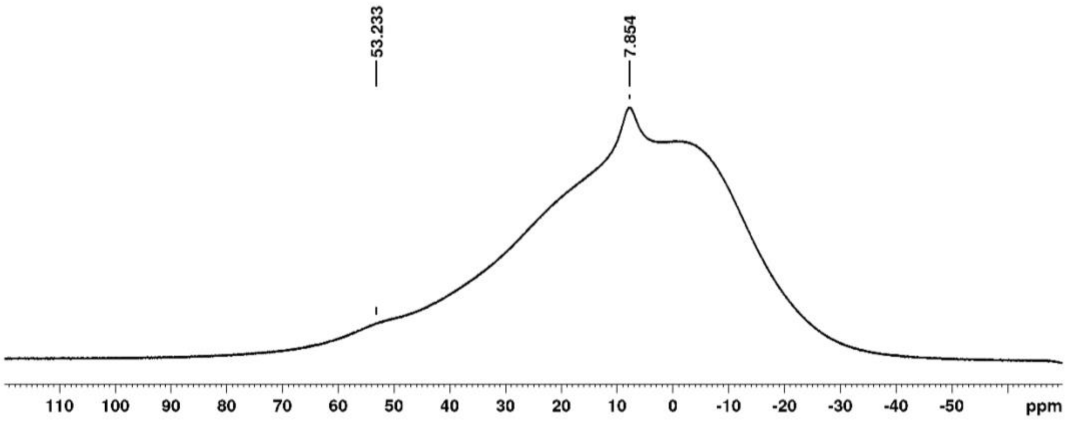

Figure S34. ^{11}B NMR spectrum (160.5 MHz) of **4d** in C_6D_6.

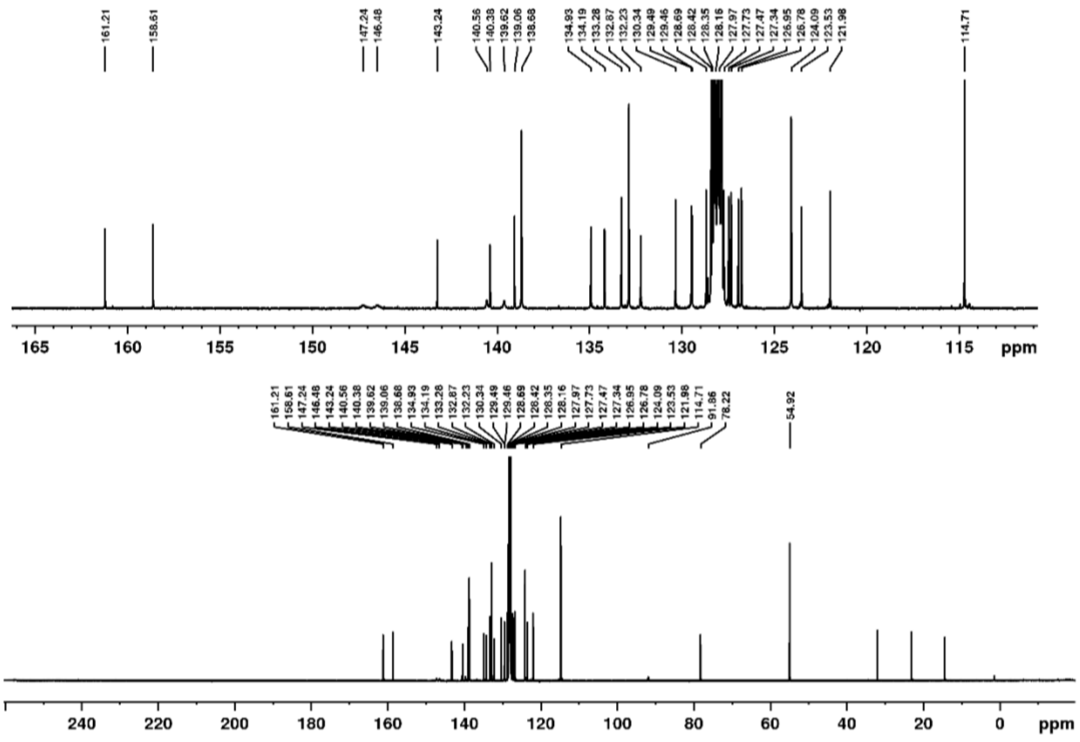

Figure S35. $^{13}C\{^1H\}$ NMR spectrum (125.8 MHz) of **4d** in C_6D_6.

2.7 Compound 4e

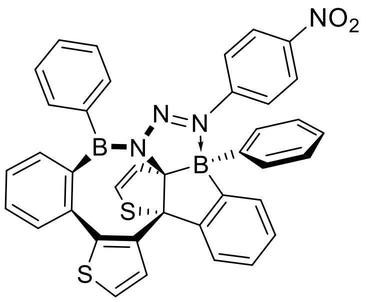

In a Young NMR tube, *p*-nitrophenyl azide (10.5 mg, 60.9 µmol, 1.0 eq.) was added to a toluene solution of **3a** (30 mg, 60.9 µmol, 1.0 eq.) and the solution shaken. All volatiles were removed under reduced pressure and the remaining solid was washed three times with 10 mL of hexane. Compound **4e** (28.4 mg, 43.3 µmol) was isolated as an orange solid in 71% yield.

^{1}H NMR (500 MHz, 298 K, C_6D_6): δ(ppm) = 7.71–7.69 (d, 1H, $^3J_{HH}$ = 6.7 Hz, Ar-*H*), 7.63–7.60 (m, 3H, Ar-*H*), 7.56–7.54 (m, 1H, Ar-*H*), 7.36–7.33 (m, 2H, Ar-*H*), 7.31–7.28 (m, 2H, Ar-*H*), 7.28–7.17 (m, 7H, Ar-*H*), 7.15–7.03 (m, 4H, Ar-*H*), 6.56 (d, 1H, $^3J_{HH}$ = 5.4 Hz, S-C*H*), 6.15 (d, 1H, $^3J_{HH}$ = 5.4 Hz, S-CH-C*H*), 5.38 (d, 1H, $^3J_{HH}$ = 6.1 Hz, S-C*H*), 4.58 (d, 1H, $^3J_{HH}$ = 6.1 Hz, S-C*H*). **^{11}B NMR** (160 MHz, 298 K, C_6D_6) δ(ppm) = 56.7, 7.95. **^{13}C{^{1}H} NMR**

(126 MHz, 298 K, C_6D_6): δ(ppm) = 158.2 (Ar-C_q), 147.7 (Ar-C_q), 146.1 (B-C_q), 145.5 (B-C_q), 144.7 (Ar-C_q), 143.2 (Ar-C_q), 139.8 (Ar-C_q), 139.4 (Ar-C_q), 139.3 (B-C_q), 139.2 (B-C_q), 138.9 (Ar-*C*H), 135.7 (Ar-*C*H), 133.2 (Ar-*C*H), 133.1 (Ar-*C*H), 132.7 (Ar-*C*H), 130.4 (Ar-*C*H), 130.3 (Ar-*C*H), 129.3 (Ar-*C*H), 129.2 (Ar-*C*H), 129.0 (Ar-*C*H), 128.6 (Ar-*C*H), 128.1 (Ar-*C*H), 128.0 (Ar-*C*H), 127.5 (Ar-*C*H), 127.2 (Ar-*C*H), 126.7 (Ar-*C*H), 124.8 (Ar-*C*H), 123.8 (Ar-*C*H), 122.3 (Ar-*C*H), 120.3 (Ar-*C*H), 119.1(Ar-*C*H), 93.5 (B-C_q) 78.3 (*C*q), 54.9 (O*C*H_3),

HRMS (LIFDI, m/z): $[C_{38}H_{26}B_2N_4S_2O_2]^+$ calculated: 656.1678; found: 656.1666.

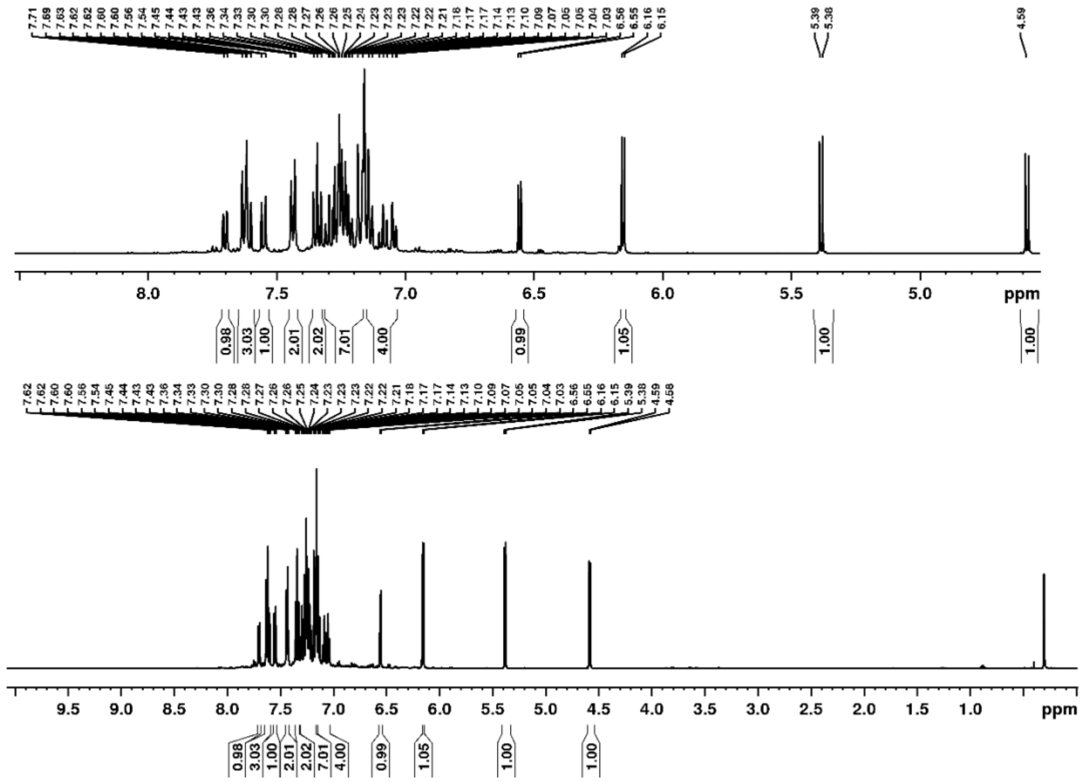

Figure S36. ^{1}H NMR spectrum (500.1 MHz) of **4e** in C_6D_6.

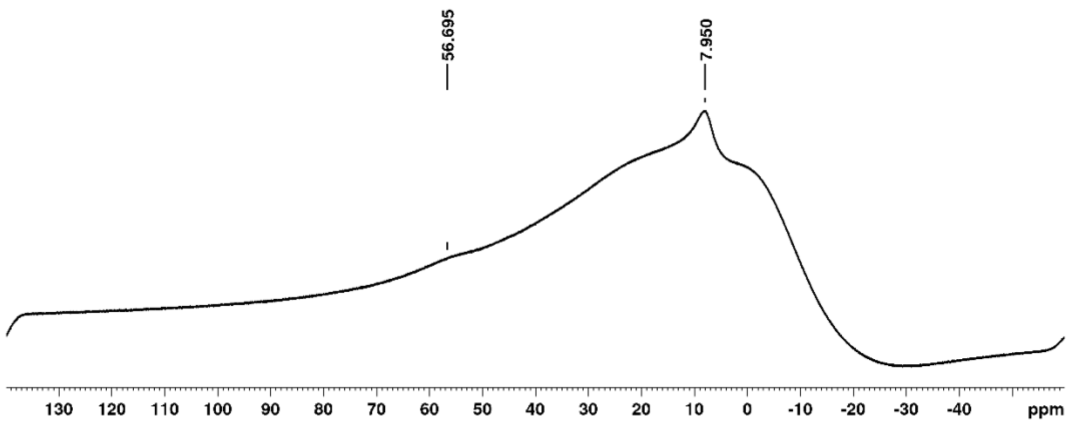

Figure S37. ^{11}B NMR spectrum (160.5 MHz) of **4e** in C_6D_6.

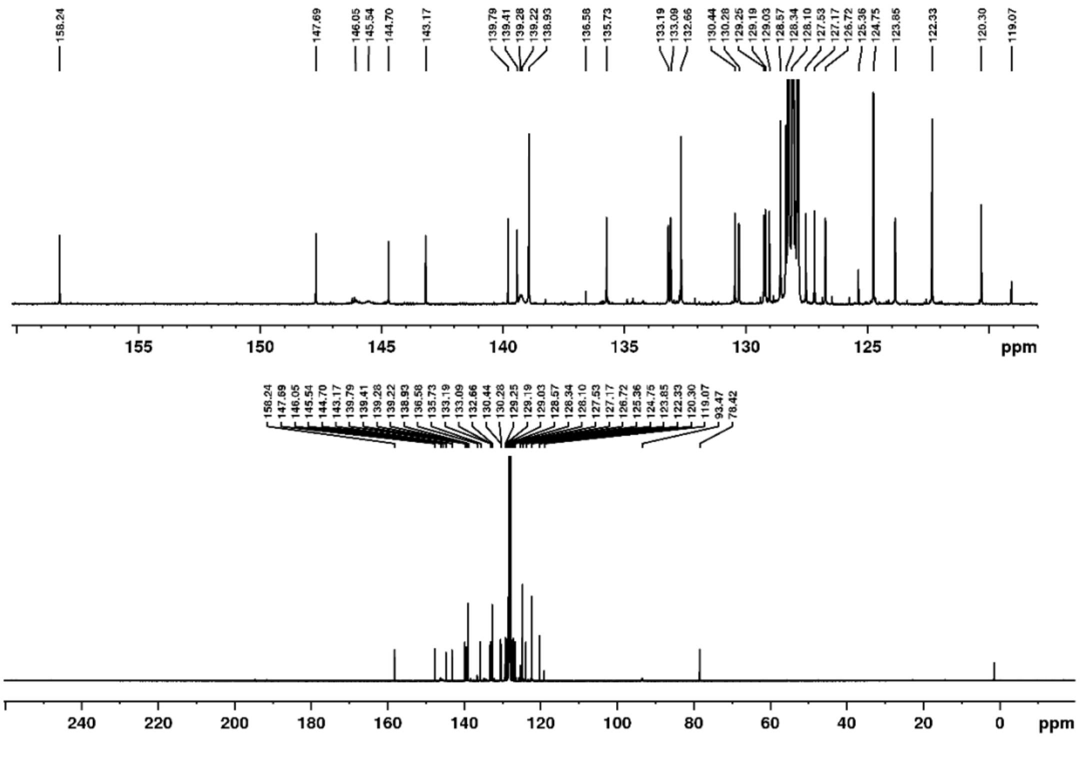

Figure S38. $^{13}C\{^1H\}$ NMR spectrum (125.8 MHz) of **4e** in C_6D_6.

3. CRYSTALLOGRAPHIC DETAILS

The crystal data of **2c** were collected on a BRUKER X8-APEX II diffractometer with a CCD area detector and multi-layer mirror monochromated $Mo_{K\alpha}$ radiation. The structure was solved using the intrinsic phasing method[4], refined with the SHELXL program[5] and expanded using Fourier techniques. All non-hydrogen atoms were refined anisotropically. Hydrogen atoms were included in structure factors calculations. All hydrogen atoms were assigned to idealised geometric positions.

In each molecule, the displacement parameters of the atoms of only one of the two thiophene-benzo-fused borole backbones were restrained to the same value with the similarity restraint SIMU. In each molecule, the 1-2 and 1-3 distances in one of the two thiophene-benzo-fused borole backbones were restrained to the same values with SAME.

Crystal data for **2c**: $C_{19}H_{17}BS$, M_r = 288.19, orange block, 0.385×0.286×0.253 mm^3, monoclinic space group $P2_1/c$, a = 13.289(6) Å, b = 30.280(16) Å, c = 7.763(5) Å, β = 93.30(2)°, V = 3119(3) Å^3, Z = 8, ρ_{calcd} = 1.228 g·cm^{-3}, μ = 0.197 mm^{-1}, F(000) = 1216, T = 99(2) K, R_1 = 0.0821, wR^2 = 0.1211, 6429 independent reflections [2θ≤52.924°] and 585 parameters. CCDC-2126040.

The crystal data of **3a** were collected on a RIGAKU OD XTALAB SYNERGY-S diffractometer with a HPAD area detector and multi-layer mirror monochromated $Cu_{K\alpha}$ radiation. The structure was solved using the intrinsic phasing method[4], refined with the SHELXL program[5] and expanded using Fourier techniques. All non-hydrogen atoms were refined anisotropically. Hydrogen atoms were included in structure factors calculations. All hydrogen atoms were assigned to idealised geometric positions. The structure was refined using the TWIN keyword (matrix: –1 0 0 0 –1 0 0.004 0 1). The BASF parameter was refined to 7.4%.

Crystal data for **3a**: $C_{32}H_{22}B_2S_2$, M_r = 492.23, clear yellow block, 0.240×0.189×0.136 mm^3, monoclinic space group $P2_1$, a = 11.42010(10) Å, b = 17.97960(10) Å, c = 12.12770(10) Å, α = 90°, β = 90.1200(10)°, γ = 90°, V = 2490.16(3) Å^3, Z = 4, ρ_{calcd} = 1.313 g·cm^{-3}, μ = 2.074 mm^{-1}, F(000) = 1024, T = 100.00(10) K, R_1 = 0.0305, wR^2 = 0.0819, Flack parameter = 0.001(8), 10291 independent reflections [2θ≤154.66°] and 650 parameters. CCDC-2126041.

The crystal data of **3b** were collected on a BRUKER X8-APEX II diffractometer with a CCD area detector and multi-layer mirror monochromated $Mo_{K\alpha}$ radiation. The structure was solved using intrinsic phasing method[4], refined with the SHELXL program[5] and expanded using Fourier techniques. All non-hydrogen atoms were refined anisotropically. Hydrogen atoms were included in structure factors calculations. All hydrogen atoms were assigned to idealised geometric positions.

Crystal data for **3b**: $C_{36}H_{30}B_2S_2$, M_r = 548.34, colourless block, 0.40×0.176×0.112 mm^3, monoclinic space group *C*2/*c*, *a* = 14.644(7) Å, *b* = 11.056(4) Å, *c* = 17.829(8) Å, β = 94.90(2)°, *V* = 2876(2) Å^3, *Z* = 4, ρ_{calcd} = 1.266 g·cm^{-3}, μ = 0.210 mm^{-1}, *F*(000) = 1152, *T* = 100(2) K, R_1 = 0.1042, wR^2 = 0.1490, 3064 independent reflections [2θ≤53.554°] and 183 parameters. CCDC-2126042.

The crystal data of **4a** were collected on a BRUKER D8-QUEST diffractometer with a CMOS area detector and multi-layer mirror monochromated $Mo_{K\alpha}$ radiation. The structure was solved using intrinsic phasing method[4], refined with the SHELXL program[5] and expanded using Fourier techniques. All non-hydrogen atoms were refined anisotropically. Hydrogen atoms were included in structure factors calculations. All hydrogen atoms were assigned to idealised geometric positions. Disorder of the solvent molecules was done with the Disordered Structure Refinement (DSR) Plugin, Version: 228.[6]

The displacement parameters of atoms of the solvent molecules were restrained to the same value with the similarity restraint SIMU. The distances between atoms of the solvent molecules were restrained during refinement to the same value with the SADI restraint. The Uii displacement parameters of atoms of the solvent molecule residues were restrained with the ISOR keyword to approximate isotropic behaviour. The atomic displacement parameters of atoms of the solvent molecules were restrained with the RIGU keyword in the ShelXL input ('enhanced rigid bond' restraint for all bonds in the connectivity list. Standard values of 0.004 for both parameters s1 and s2 were used). The 1-2 and 1-3 distances in the solvent molecules were restrained to the same values with SAME.
Crystal data for **4a** $C_{44.25}H_{33.51}B_2N_3S_2$, M_r = 693.04, orange block, 0.388×0.221×0.107 mm^3, triclinic space group $P\bar{1}$, *a* = 10.278(3) Å, *b* = 12.065(3) Å, *c* = 16.702(4) Å, α = 92.946(9)°, β = 107.906(8)°, γ = 114.158(16)°, *V* = 1760.3(8) Å^3, *Z* = 2, ρ_{calcd} = 1.308 g·cm^{-3}, μ = 0.189 mm^{-1}, *F*(000) = 724, *T* = 100(2) K, R_1 = 0.0504, wR^2 = 0.0965, 7225 independent reflections [2θ≤52.94°] and 460 parameters. CCDC-2126043.

The crystal data of **4b** were collected on a BRUKER D8-QUEST diffractometer with a CPA area detector and multi-layer mirror monochromated $Mo_{K\alpha}$ radiation. The structure was solved using intrinsic phasing method[4], refined with the SHELXL program[5] and expanded using Fourier techniques. All non-hydrogen atoms were refined anisotropically. Hydrogen atoms were included in structure factors calculations. All hydrogen atoms were assigned to idealised geometric positions.

Crystal data for **4b**: $C_{41}H_{37}B_2N_3S_2Si$, M_r = 685.56, orange block, 0.196×0.096×0.087 mm^3, triclinic space group $P\bar{1}$, *a* = 9.9577(13) Å, *b* = 12.2845(16) Å, *c* = 16.579(5) Å, α = 100.823(7)°, β = 90.407(8)°, γ = 112.635(10)°, *V* = 1831.5(7) Å^3, *Z* = 2, ρ_{calcd} = 1.243 g·cm^{-3}, μ = 0.212 mm^{-1},

$F(000) = 720$, $T = 100(2)$ K, $R_1 = 0.0386$, $wR_2 = 0.0779$, 6683 independent reflections [2θ≤50.698°] and 445 parameters. CCDC-2126044.

The crystal data of **4e** were collected on a BRUKER D8-QUEST diffractometer with a CMOS area detector and multi-layer mirror monochromated $Mo_{K\alpha}$ radiation. The structure was solved using intrinsic phasing method[4], refined with the SHELXL program[5] and expanded using Fourier techniques. All non-hydrogen atoms were refined anisotropically. Hydrogen atoms were included in structure factors calculations. All hydrogen atoms were assigned to idealised geometric positions.

Crystal data for **4e**: $C_{38}H_{26}B_2N_4O_2S_2$, $M_r = 656.37$, red block, 0.25×0.137×0.133 mm^3, Monoclinic space group $P2_1/n$, $a = 13.299(3)$ Å, $b = 18.859(4)$ Å, $c = 13.574(3)$ Å, $\beta = 112.808(10)°$, $V = 3138.2(12)$ $Å^3$, $Z = 4$, $\rho_{calcd} = 1.389$ $g{\cdot}cm^{-3}$, $\mu = 0.213$ mm^{-1}, $F(000) = 1360$, $T = 100(2)$ K, $R_1 = 0.0875$, $wR^2 = 0.1392$, 5731 independent reflections [2θ≤50.7°] and 433 parameters. CCDC-2126045.

The crystal data of **2-pyr** were collected on a BRUKER X8-APEX II diffractometer with a CCD area detector and multi-layer mirror monochromated $Mo_{K\alpha}$ radiation. The structure was solved using intrinsic phasing method[4], refined with the SHELXL program[5] and expanded using Fourier techniques. All non-hydrogen atoms were refined anisotropically. Hydrogen atoms were included in structure factors calculations. All hydrogen atoms were assigned to idealised geometric positions.

The displacement parameters of the atoms of the thiophene-benzo-fused borole backbone were restrained to the same value with the similarity restraint SIMU. The U_{ii} displacement parameters of the atoms of the thiophene-benzo-fused borole backbone were restrained with the ISOR keyword to approximate isotropic behaviour.

Crystal data for **2-pyr**: $C_{21}H_{16}BNS$, $M_r = 325.22$, colourless block, 0.60×0.202×0.182 mm^3, monoclinic space group $P2_1/c$, $a = 8.800(5)$ Å, $b = 10.843(6)$ Å, $c = 17.829(10)$ Å, $\beta = 99.74(2)°$, $V = 1676.7(15)$ $Å^3$, $Z = 4$, $\rho_{calcd} = 1.288$ $g{\cdot}cm^{-3}$, $\mu = 0.193$ mm^{-1}, $F(000) = 680$, $T = 100(2)$ K, $R_1 = 0.0763$, $wR_2 = 0.1551$, 3069 independent reflections [2θ≤50.684°] and 317 parameters. CCDC-2126039.

The crystal data of **2-P** were collected on a FOUR-CIRCLE DIFFRACTOMETER with a CPA area detector and multi-layer mirror monochromated $Mo_{K\alpha}$ radiation. The structure was solved using intrinsic phasing method[4], refined with the SHELXL program[5] and expanded using Fourier techniques. All non-hydrogen atoms were refined anisotropically. Hydrogen atoms were included in structure factors calculations. All hydrogen atoms were assigned to idealised geometric positions.

The displacement parameters of the atoms of the thiophene-benzo-fused borole backbone were restrained to the same value with the similarity restraint SIMU. The Uii displacement parameters of the atoms of the thiophene-benzo-fused borole backbone were restrained with the ISOR keyword to approximate isotropic behaviour.

Crystal data for **2-P**: $C_{37}H_{32}BPS$, M_r = 550.46, colourless block, 0.251×0.147×0.144 mm^3, monoclinic space group *Cc*, *a* = 20.883(11) Å, *b* = 8.704(4) Å, *c* = 19.093(7) Å, β = 123.59(3)°, *V* = 2891(2) Å^3, *Z* = 4, ρ_{calcd} = 1.265 g·cm^{-3}, μ = 0.193 mm^{-1}, *F*(000) = 1160, *T* = 100(2) K, R_1 = 0.0765, wR_2 = 0.1255, Flack parameter = -0.03(7), 6147 independent reflections [2θ≤53.856°] and 465 parameters. CCDC-2126038.

Data	**2c**	**3a**	**3b**	**4a**
Empirical formula	$C_{19}H_{17}BS$	$C_{32}H_{22}B_2S_2$	$C_{36}H_{30}B_2S_2$	$C_{44.25}H_{33.51}B_2N_3S_2$
Formula weight (g·mol^{-1})	288.19	492.23	548.34	693.04
Temperature (K)	99(2)	100.00(10)	100(2)	100(2)
Radiation, λ (Å)	$Mo_{K\alpha}$ 0.71073	$Cu_{K\alpha}$1.54184	$Mo_{K\alpha}$0.71073	$Mo_{K\alpha}$0.71073
Crystal system	Monoclinic		Monoclinic	Triclinic
Space group	$P2_1/c$	$P2_1$	*C*2/*c*	$P\bar{1}$
Unit cell dimensions				
a (Å)	13.289(6)	11.42010(10)	14.644(7)	10.278(3)
b (Å)	30.280(16)	17.97960(10)	11.056(4)	12.065(3)
c (Å)	7.763(5)	12.12770(10)	17.829(8)	16.702(4)
α (°)	90	90	90	92.946(9)
β (°)	93.30(2)	90.1200(10)	94.90(2)	107.906(8)
γ (°)	90	90	90	114.158(16)
Volume (Å^3)	3119(3)	2490.16(3)	2876(2)	1760.3(8)
Z	8	4	4	2
Calculated density (mg·m^{-3})	1.228	1.313	1.266	1.308
Absorption coefficient (mm^{-1})	0.197	2.074	0.210	0.189
F(000)	1216	1024	1152	724
Theta range for collection	1.345 to 26.462°	2.457 to 77.330°	2.293 to 26.777°	2.222 to 26.470°
Reflections collected	28969	57553	12973	26653
Independent reflections	6429	10291	3064	7225
Minimum/maximum transmission	0.6440/0.7454	0.77997/1.00000	0.5375/0.7454	0.6452/0.7454
Refinement method	Full-matrix least-squares on F^2	Full-matrix least-squares on F^2	Full-matrix least-squares on F^2	Full-matrix least-squares on F^2
Data / parameters / restraints	6429 / 585 / 200	10291 / 650 / 1	3064 / 183 / 0	7225 / 460 / 90
Goodness-of-fit on F^2	1.174	1.022	1.038	1.024
Final R indices [I>2σ(I)]	R_1 = 0.0602, wR^2 = 0.1139	R_1 = 0.0305, wR^2 = 0.0819	R_1 = 0.0585, wR^2 = 0.1294	R_1 = 0.0381, wR^2 = 0.0890

R indices (all data)	R_1 = 0.0821, wR^2 = 0.1211	R_1 = 0.0305, wR^2 = 0.0819	R_1 = 0.1042, wR^2 = 0.1490	R_1 = 0.0504, wR^2 = 0.0965
Maximum/minimum residual electron density (e·Å^{-3})	0.316 / –0.232	0.457 / –0.245	0.385 / –0.372	0.333 / –0.329
Flack parameter		0.001(8)		

Data	**4b**	**4e**
Empirical formula	$C_{41}H_{37}B_2N_3S_2Si$	$C_{38}H_{26}B_2N_4O_2S_2$
Formula weight (g·mol^{-1})	685.56	656.37
Temperature (K)	100(2)	100(2)
Radiation, λ (Å)	$Mo_{K\alpha}$, 0.71073	$Mo_{K\alpha}$0.71073
Crystal system	Triclinic	Monoclinic
Space group	$P\bar{1}$	*P*2$_1$/*n*
Unit cell dimensions		
a (Å)	9.9577(13)	13.299(3)
b (Å)	12.2845(16)	18.859(4)
c (Å)	16.579(5)	13.574(3)
α (°)	100.823(7)	90
β (°)	90.407(8)	112.808(10)
γ (°)	112.635(10)	90
Volume (Å^3)	1831.5(7)	3138.2(12)
Z	2	4
Calculated density (mg·m^{-3})	1.243	1.389
Absorption coefficient (mm^{-1})	0.212	0.213
F(000)	720	1360
Theta range for collection	1.999 to 25.349°	1.953 to 25.350°
Reflections collected	57499	55262
Independent reflections	6683	5731
Minimum/maximum transmission	0.6648/0.7456	0.6075/0.7452
Refinement method	Full-matrix least-squares on F^2	Full-matrix least-squares on F^2
Data / parameters / restraints	6683 / 445 / 0	5731 / 433 / 0
Goodness-of-fit on F^2	1.036	1.062
Final R indices [I>2σ(I)]	R_1 = 0.0312, wR_2 = 0.0738	R_1 = 0.0514, wR^2 = 0.1197
R indices (all data)	R_1 = 0.0386, wR_2 = 0.0779	R_1 = 0.0875, wR^2 = 0.1392
Maximum/minimum residual electron density (e·Å^{-3})	0.290 / –0.267	0.424 / –0.599

Data	**2-pyr**	**2-P**
Empirical formula	$C_{21}H_{16}BNS$	$C_{37}H_{32}BPS$
Formula weight (g·mol^{-1})	325.22	550.46
Temperature (K)	100(2)	100(2)
Radiation, λ (Å)	$Mo_{K\alpha}$, 0.71073	$Mo_{K\alpha}$0.71073
Crystal system	Monoclinic	Monoclinic
Space group	*P*2$_1$/*c*	*Cc*
Unit cell dimensions		
a (Å)	8.800(5)	20.883(11)
b (Å)	10.843(6)	8.704(4)
c (Å)	17.829(10)	19.093(7)
α (°)	90	90

β (°)	99.74(2)	123.59(3)
γ (°)	90	90
Volume ($Å^3$)	1676.7(15)	2891(2)
Z	4	4
Calculated density ($mg{\cdot}m^{-3}$)	1.288	1.265
Absorption coefficient (mm^{-1})	0.193	0.193
F(000)	680	1160
Theta range for collection	2.207 to 25.342°	2.325 to 26.928°
Reflections collected	15397	24459
Independent reflections	3069	6147
Minimum/maximum transmission	0.5792/0.7454	0.5184/0.7454
Refinement method	Full-matrix least-squares on F^2	Full-matrix least-squares on F^2
Data / parameters / restraints	3069 / 317 / 276	6147 / 465 / 278
Goodness-of-fit on F^2	1.271	1.048
Final R indices [I>2σ(I)]	R_1 = 0.0691, wR_2 = 0.1525	R_1 = 0.0526, wR^2 = 0.1124
R indices (all data)	R_1 = 0.0763, wR_2 = 0.1551	R_1 = 0.0765, wR^2 = 0.1255
Maximum/minimum residual electron density ($e{\cdot}Å^{-3}$)	0.267 / –0.257	0.368 / –0.531
Flack parameter		-0.03(7)

4. COMPUTATIONAL DETAILS

All computations were carried out using the Gaussian 09 (Revision E.01) software.[7] Geometry optimisations, with no symmetry constraints, and vibrational frequency calculations were performed using the hybrid DFT functional B3LYP[8] augumented with the Grimme's D3 correction (GD3) and Becke-Johnson damping (BJ).[9] Basis sets employed in optimisation and frequency analysis are LANL2DZ (for Sn and Br) and 6-31G(d,p) for other atoms. Transition state geometries were located using the algorithm opt = (ts, noeigentest, calcfc).[10] All optimised transition state structures were confirmed as a first-order saddle point with only one imaginary frequency, and the magnitudes of all frequencies were greater than the residual frequencies related to rotations and translations. Additionally, each transition state found was made sure to be on the desired reaction coordinate by performing the "plus-and-minus-displacement" minimisation computations, which involve in the displacement of the transition state structure by ~0.05 Å or 5° to 10° by the imaginary frequency normal mode in both directions,[11] and the displaced geometries were then optimised to the closest minimum structures. The zero-point vibrational energies (ZPVE) and thermal corrections were computed from frequency calculations with a standard state of 298 K and 1 atm. Single-point energies were computed on the optimised geometries using the B3LYP-D3/6-311G++** (def2TZVP for Sn and Br) level of theory augmented with the SMD solvation model (SCRF = SMD) to include the benzene solvent effect.[12] The energies (ΔG) reported are corrected for ZPVEs. Information on the antiaromatic character of boroles was obtained from the nucleus-independent chemical shift (NICS) calculations[13] using the GIAO method[14] with the model chemistry B3LYP-D3BJ/6-311++g(2d,2p)//B3LYP-D3BJ/6-31g(d,p). The NICS values were obtained by including the ghost atoms in the axis perpendicular to the ring plane at a distance of zero (NICS(0)) and one (NICS(1)) angstrom from the centroid of the borole ring.

4.1 Optimised structures, Cartesian coordinates, and energies

4.1.1 Thermodynamic stability of boroles and their diboracyclic dimers

Note: Computed details about compounds **2a** and **3a** are provided in Section 4.1.2.

1) Borole **2b**

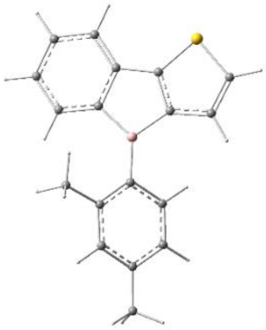

Number of imaginary frequencies = 0

E_{total} = -1118.117351 a.u

$G_{correction}$ = 0.231202 a.u

Cartesian coordinates:

C	0.84506600	2.53866500	-0.35476800
C	1.02990700	1.18079700	-0.13641800
C	2.36288100	0.67156600	-0.12554100
C	3.46094600	1.49806800	-0.28935300
C	3.24332000	2.87472200	-0.48372700
C	1.95361600	3.39038200	-0.52463200
H	-0.15764400	2.95285600	-0.39703800
H	4.47121800	1.09921100	-0.27837200
H	4.09475800	3.53632100	-0.61260300
H	1.80043700	4.45262500	-0.68825000
C	2.28965500	-0.78877900	0.03940000
C	0.99298300	-1.28221700	0.10103100
B	0.03680600	-0.04892300	0.02819400
S	3.48376400	-2.02423600	0.17641400
C	0.97896700	-2.70058100	0.27306700
H	0.07803300	-3.29750700	0.35840800

C	2.23453800	-3.24519300	0.32503000
H	2.51854700	-4.28127000	0.44430800
C	-1.50850800	-0.10100200	0.02866400
C	-2.37709200	0.80917500	0.67560700
C	-2.09631800	-1.14090200	-0.72699200
C	-3.76019500	0.66840100	0.51994900
C	-3.46988400	-1.25034600	-0.89203600
H	-1.44694800	-1.86436200	-1.21184100
C	-4.32826600	-0.33591200	-0.26738200
H	-4.41847900	1.36297600	1.03826500
H	-3.88465400	-2.05351400	-1.49550400
C	-1.87445000	1.89969500	1.59474400
H	-2.35167500	1.81970900	2.57757500
H	-0.79592100	1.85355000	1.74209400
H	-2.11959600	2.89416900	1.20382800
C	-5.82194300	-0.43212600	-0.44660900
H	-6.15823400	-1.47356200	-0.43819000
H	-6.35599400	0.10615800	0.34096900
H	-6.12944200	-0.00146200	-1.40744300

2) Diboracycle **3b**

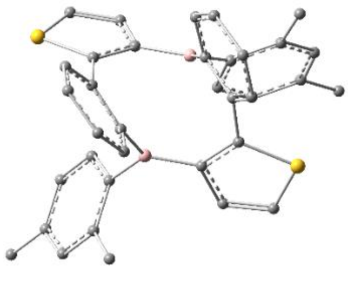

Number of imaginary frequencies = 0

E_{total} = -2236.289617 a.u

$G_{correction}$ = 0.489423 a.u

Cartesian coordinates:

C	-2.00909300	-0.92223400	1.13416600
B	-1.12477900	0.36901700	1.17042600

C	-2.02559000	-3.37341300	0.98834900
H	-1.47053600	-4.30741400	0.96044100
C	-1.34806500	-2.16724400	1.11917800
H	-0.26555600	-2.18715200	1.17784900
C	-3.41960400	-3.38779400	0.89631800
C	-4.09204600	-2.16236000	0.94062400
H	-5.17877000	-2.16113100	0.88968300
C	-3.42526200	-0.93815800	1.04032000
C	-4.26353100	0.32047600	1.05677400
H	-5.30870200	0.07966400	1.26958600
H	-4.22960200	0.83437100	0.09094700
H	-3.92063500	1.03835300	1.80476400
C	-4.17779000	-4.67974900	0.72833000
H	-4.32103500	-4.91409300	-0.33397600
H	-5.17047900	-4.62467400	1.18470700
H	-3.64022700	-5.52025600	1.17671700
C	0.15744400	0.39589600	2.04921400
C	0.31211100	-0.38829100	3.25090600
H	-0.43982500	-1.09136800	3.58742200
C	1.46497600	-0.14063600	3.93552500
H	1.80348300	-0.57775000	4.86451500
S	2.44219900	1.04500700	3.12320800
C	1.26169200	1.22868500	1.85575800
C	1.52209500	2.12908400	0.72733900
C	1.92156900	3.45370600	0.94911800
H	2.09327900	3.79366200	1.96611800
C	2.04641600	4.34074300	-0.11632800
H	2.34845100	5.36742600	0.06570100
C	1.74955300	3.91222200	-1.41055800
H	1.81970400	4.60506400	-2.24375800
C	1.35985500	2.59227400	-1.63406200
H	1.12643200	2.26918500	-2.64427800
C	1.27101600	1.66734300	-0.58341200
C	2.21623500	-0.86132900	-0.72817400
B	1.01928100	0.15000200	-0.91336600

C	3.49947200	-2.48900300	0.56570800
H	3.63634500	-3.03595700	1.49521900
C	2.44170000	-1.58888000	0.44957000
H	1.78460900	-1.45352300	1.29956400
C	4.38134900	-2.69579100	-0.49660000
C	4.16410100	-1.97136200	-1.67287700
H	4.83888300	-2.11630400	-2.51431200
C	3.11161300	-1.06211500	-1.80580000
C	2.95708300	-0.28628600	-3.09402900
H	3.65151900	-0.64462300	-3.85850600
H	3.15523500	0.77999000	-2.93627300
H	1.94202000	-0.36677600	-3.49838700
C	5.54896500	-3.64308200	-0.37413800
H	6.44402000	-3.12415900	-0.00895300
H	5.80509200	-4.08984300	-1.33959800
H	5.33429100	-4.45332800	0.32897300
C	-0.26638100	-0.33579200	-1.62242000
C	-0.35283900	-1.58763200	-2.33223900
H	0.47113500	-2.28995900	-2.37229800
C	-1.54470800	-1.79144100	-2.95818200
H	-1.85509700	-2.63257000	-3.56198800
S	-2.64381800	-0.47099000	-2.67511900
C	-1.46458000	0.37464800	-1.71810000
C	-1.83005600	1.60165600	-1.00198900
C	-2.37792300	2.70440500	-1.66754400
H	-2.56724500	2.64429300	-2.73526800
C	-2.63377700	3.88436400	-0.97250600
H	-3.05181200	4.73984900	-1.49401700
C	-2.32072700	3.97299100	0.38518800
H	-2.49626500	4.89935700	0.92383400
C	-1.78114000	2.87177700	1.04830600
H	-1.54054000	2.94630000	2.10514400
C	-1.56159600	1.65798900	0.38422000

3) Borole **2b**

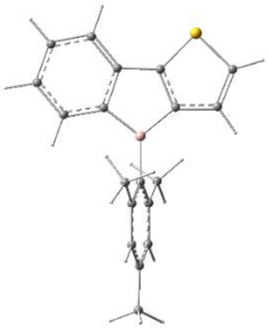

Number of imaginary frequencies = 0

E_{total} = -1157.441603 a.u

$G_{correction}$ = 0.255989 a.u

Cartesian coordinates:

C	0.79253900	2.55498500	-0.43828100
C	1.04593200	1.21661300	-0.18183400
C	2.39762800	0.76926900	-0.12540000
C	3.45691600	1.64094200	-0.30491600
C	3.17384600	2.99868000	-0.55067500
C	1.86236100	3.45356200	-0.61980700
H	-0.23288700	2.91123100	-0.49658900
H	4.48578600	1.29526800	-0.26269100
H	3.99462400	3.69547200	-0.69227300
H	1.66361200	4.50280700	-0.81494100
C	2.37813500	-0.68331300	0.12092100
C	1.09967200	-1.22250800	0.20549800
B	0.09933300	-0.03847300	0.02873600
S	3.61555700	-1.86296400	0.33409300
C	1.13736200	-2.62835900	0.45611700
H	0.25813800	-3.25313400	0.56663300
C	2.41198100	-3.12052600	0.54676900
H	2.73446500	-4.13641300	0.72647000
C	-1.45330400	-0.05826900	0.03068000
C	-2.19069500	0.60267900	1.03740400
C	-2.15518700	-0.73979900	-0.99212700
C	-3.58809800	0.57507200	1.00725900
C	-3.54973500	-0.73586200	-0.99889200

C	-4.28684100	-0.08257900	-0.00579900
H	-4.14354800	1.07756000	1.79595100
H	-4.07648000	-1.25284700	-1.79811700
C	-1.48867300	1.27646600	2.19441600
H	-1.12603600	0.53590600	2.91793300
H	-0.61854400	1.85011600	1.86450000
H	-2.16223600	1.95240400	2.72821400
C	-5.79453500	-0.07244800	-0.04674900
H	-6.19278800	-1.05037800	-0.33543500
H	-6.21889500	0.19421900	0.92505900
H	-6.16534300	0.65528100	-0.77901300
C	-1.40515700	-1.43437200	-2.10625200
H	-0.83804200	-0.71775700	-2.71225000
H	-0.68238500	-2.15839200	-1.71793900
H	-2.08988700	-1.96293800	-2.77468000

4) Diboracycle **3c**

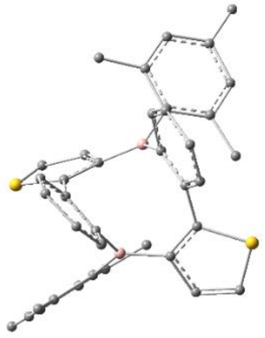

Number of imaginary frequencies = 0

E_{total} = -2314.927386 a.u

$G_{correction}$ = 0.542197 a.u

Cartesian coordinates:

C	2.75050600	-1.00593300	-0.17306900
B	1.42089300	-0.20117900	-0.46156000
C	4.54393600	-2.46977600	-0.93114100
H	5.00431900	-3.04376200	-1.73256800
C	3.35568300	-1.77608500	-1.19325700

C	5.14825500	-2.45151500	0.32350000
C	4.54472100	-1.68837500	1.32766200
H	4.99956000	-1.65274900	2.31539700
C	3.38097000	-0.95772000	1.09342900
C	2.82311200	-0.10471700	2.20622600
H	3.26942600	-0.36694200	3.16907100
H	1.73895500	-0.19759500	2.30832900
H	3.03209400	0.95345200	2.02208000
C	6.41932200	-3.21649600	0.59544700
H	6.28998600	-3.92053900	1.42514400
H	7.23743200	-2.54184200	0.87288400
H	6.73811500	-3.78584800	-0.28169900
C	0.20210200	-1.04148100	-0.92796200
C	0.17926300	-2.48308300	-0.82150400
H	0.91812000	-3.03265100	-0.25194800
C	-0.83652900	-3.07948600	-1.50105500
H	-1.07278600	-4.13174600	-1.57334100
S	-1.82078000	-1.90663300	-2.32901100
C	-0.86294500	-0.59562400	-1.71310200
C	-1.27452600	0.79464400	-1.94467000
C	-1.31010900	1.36875500	-3.21927400
H	-1.05741200	0.76626600	-4.08670600
C	-1.63055100	2.71741000	-3.36141300
H	-1.65164300	3.16806500	-4.34886400
C	-1.89404600	3.49281000	-2.22971900
H	-2.12047300	4.54935400	-2.33765700
C	-1.86207000	2.91293400	-0.96329000
H	-2.06581800	3.52257500	-0.08738700
C	-1.58757700	1.54880200	-0.79754600
C	-2.66530800	-0.36981500	0.82321700
B	-1.68023500	0.83362700	0.60237400
C	-3.11667000	-2.63523700	1.61981400
H	-2.77311700	-3.52391300	2.14429500
C	-2.26167500	-1.53020600	1.53568800
C	-4.39045900	-2.62975200	1.05940700

C	-4.80452800	-1.47156300	0.39669100
H	-5.80398600	-1.43571900	-0.03117600
C	-3.97093200	-0.36329500	0.25686100
C	-4.52610900	0.82494100	-0.49981700
H	-5.61059800	0.73275700	-0.60415300
H	-4.09939600	0.89810600	-1.50447800
H	-4.31754900	1.77338200	0.00059500
C	-5.29306800	-3.83394900	1.14758300
H	-5.37342600	-4.33821500	0.17688300
H	-6.30791300	-3.55085300	1.44592800
H	-4.91731200	-4.56357600	1.86995900
C	-0.84312500	1.42321600	1.75890700
C	-1.25996300	1.42315800	3.13864000
H	-2.16951900	0.93509800	3.46933100
C	-0.43639700	2.12135800	3.96928000
H	-0.53463500	2.28944500	5.03264700
S	0.91429500	2.78823600	3.10146300
C	0.33957900	2.12979500	1.58449000
C	1.12072000	2.39311900	0.35855300
C	1.44522400	3.73570800	0.09239700
H	1.10443200	4.49843700	0.78518500
C	2.14686000	4.10524100	-1.04811000
H	2.36464400	5.15171200	-1.23736600
C	2.56383500	3.11893000	-1.93892900
H	3.11928700	3.38200500	-2.83400800
C	2.28678800	1.78387400	-1.66054200
H	2.66516000	1.02686400	-2.34093900
C	1.55156500	1.37696200	-0.52999800
C	2.78179000	-1.89191500	-2.59207500
H	2.16316400	-1.03831700	-2.87425300
H	3.58671100	-1.98240700	-3.32760700
H	2.14589400	-2.77900000	-2.68659300
C	-0.92164900	-1.66256700	2.22555900
H	-0.63585900	-2.71512700	2.30029500
H	-0.12752800	-1.13951200	1.69736200

H	-0.95381800	-1.25351300	3.24077700

4.1.2 Mechanism for the tin-boron exchange reaction

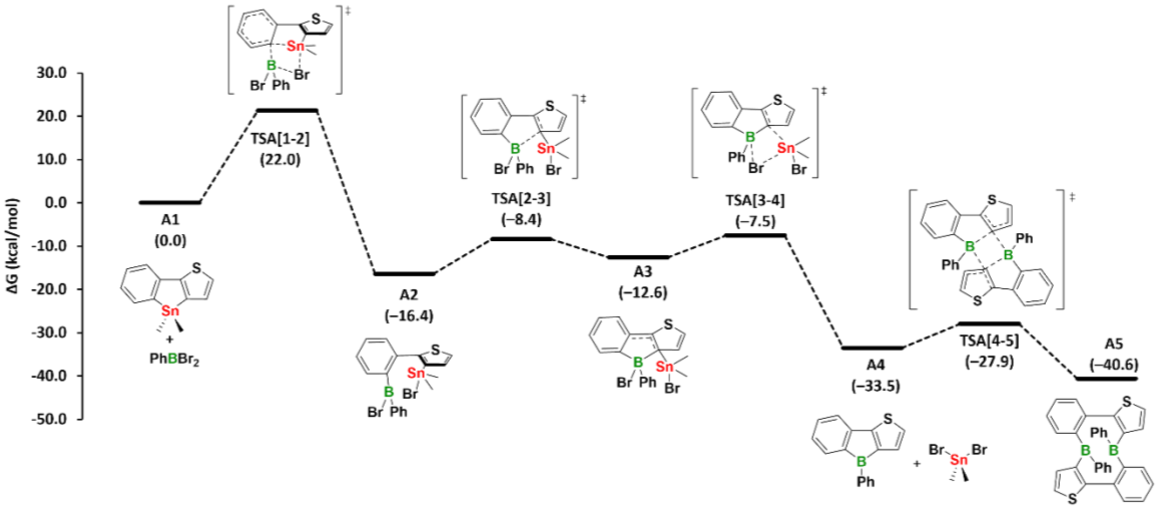

Figure S39. Computed mechanism for the formation of diboracycle **A5** from dibromo(phenyl)borane and 1,1-dimethyl-benzothienostannole (**A1**).

5) Dibromo(phenyl)borane

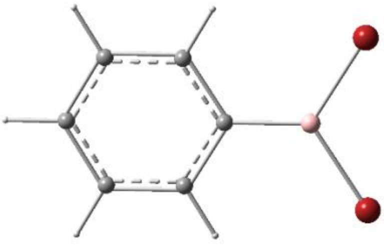

Number of imaginary frequencies = 0

E_{total} = -5400.046575 a.u

$G_{correction}$ = 0.060780 a.u

Cartesian coordinates:

Br	1.46287200	1.63785200	0.00009900
B	0.46383400	-0.00012700	-0.00017500
Br	1.46301000	-1.63778300	0.00001800
C	-1.07450800	0.00003500	-0.00031600

C	-1.80291800	-1.20859200	-0.00019700
C	-1.80301900	1.20858400	-0.00053400
C	-3.19348000	-1.21005800	0.00018600
H	-1.26290900	-2.14940900	-0.00017100
C	-3.19357900	1.20989600	-0.00002400
H	-1.26308000	2.14943500	-0.00115800
C	-3.89110400	-0.00010700	0.00036800
H	-3.73506600	-2.15076300	-0.00013000
H	-3.73525800	2.15054900	0.00056700
H	-4.97710800	-0.00013300	0.00078300

6) Benzothienostannole

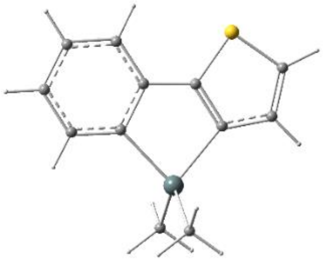

Number of imaginary frequencies = 0

E_{total} = -866.124800 a.u

$G_{correction}$ = 0.155642 a.u

Cartesian coordinates:

S	-2.84910700	-1.58032800	-0.00004800
C	-0.01685900	1.41315100	0.00000800
C	-0.25412400	-1.44151000	0.00004500
C	-1.31720300	0.83813800	0.00004600
C	0.10899500	2.80065700	-0.00000800
H	1.09528900	3.25884600	-0.00003300
C	-0.58803300	-2.82972500	-0.00001500
H	0.14139500	-3.63231900	0.00000900
C	-2.44524500	1.66724200	0.00007000
H	-3.44148600	1.23287800	0.00010500

C	-1.37971300	-0.63314200	0.00003400
C	-1.02268300	3.62588000	0.00000600
H	-0.91018500	4.70610800	-0.00001100
C	-2.29473600	3.05456700	0.00004700
H	-3.17591500	3.68944500	0.00006700
C	-1.93846500	-3.06159000	-0.00007700
H	-2.45849500	-4.00968600	-0.00011100
Sn	1.44434900	-0.15805800	0.00000000
C	2.65126800	-0.25337300	-1.76308300
H	3.34914400	0.58749400	-1.79719600
H	3.22966500	-1.18067900	-1.78751200
H	2.01656400	-0.21478700	-2.65126700
C	2.65128100	-0.25364500	1.76306000
H	3.22908400	-1.18132000	1.78764700
H	3.34969400	0.58677700	1.79703400
H	2.01660200	-0.21452300	2.65124000

7) **TSA[1-2]**

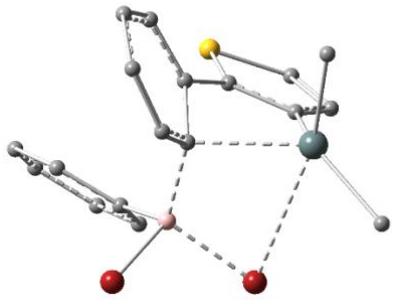

(Hydrogens are omitted for clarity)

Number of imaginary frequencies = 1 (28.0 cm^{-1})

E_{total} = -6265.550657 a.u

$G_{correction}$ = 0.242217 a.u

Cartesian coordinates:

S	-1.18699100	3.27666900	0.14999100
C	0.22694000	-0.42457700	1.04843300

C	0.90159500	1.91038300	-0.58160300
C	-0.13530700	0.93240800	1.43146100
C	0.58769600	-1.28896600	2.12651400
H	0.85808100	-2.31072000	1.88606200
C	0.77525200	2.97569700	-1.51968000
H	1.44609900	3.14349400	-2.35386400
C	-0.31247200	1.28067600	2.77241200
H	-0.62794800	2.28789000	3.02603100
C	-0.10691000	1.93187800	0.37239500
C	0.50980500	-0.89975900	3.45425100
H	0.78831100	-1.58985600	4.24381400
C	0.00332700	0.36740900	3.77489500
H	-0.10789000	0.65715300	4.81580900
C	-0.29519400	3.79001200	-1.24781700
H	-0.62330800	4.66170000	-1.79774900
Sn	2.19977300	0.29938700	-0.11333300
Br	0.25061100	-1.07784400	-2.09895000
B	-0.70255500	-1.11671900	-0.22515800
C	3.49454000	-0.82488600	-1.37477500
H	3.25721000	-1.88523100	-1.27250000
H	3.33152200	-0.54333000	-2.41585500
H	4.53318500	-0.64727500	-1.08343800
C	3.32853400	0.79719100	1.63991100
H	3.75437600	-0.10562500	2.08531200
H	4.14964000	1.47240500	1.37978000
H	2.69110500	1.28615800	2.37847000
Br	-0.97603600	-3.11804900	0.16522600
C	-2.10976300	-0.36221100	-0.29153600
C	-2.58123600	0.34356000	-1.40570100
C	-2.91445400	-0.35154500	0.86437000
C	-3.79373500	1.03813200	-1.37089500
H	-1.98480100	0.36396600	-2.31065900
C	-4.11865600	0.34433300	0.91200800

H	-2.58594600	-0.90304100	1.74163900
C	-4.56447000	1.04845100	-0.21121800
H	-4.12774700	1.57971100	-2.25180000
H	-4.71528800	0.33390600	1.82032200
H	-5.50372400	1.59337900	-0.17902600

8) Intermediate **A2**

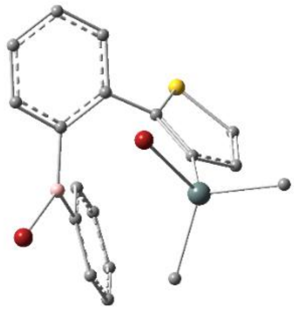

(Hydrogens are omitted for clarity)

Number of imaginary frequencies = 0

E_{total} = -6265.611446 a.u

$G_{correction}$ = 0.239707 a.u

Cartesian coordinates:

S	1.89799200	-1.66870400	2.56705200
C	0.31965400	1.61735900	1.13170600
C	0.29608400	-1.61693000	0.52706300
C	0.25984800	0.50424800	2.01420200
C	-0.19460800	2.84508900	1.59644500
H	-0.19089100	3.69656700	0.92491200
C	1.00757700	-2.86793500	0.48192900
H	0.84882000	-3.62638100	-0.27803800
C	-0.24912900	0.66775200	3.31111100
H	-0.30833300	-0.19784700	3.96317200
C	0.68557800	-0.84530900	1.60059700
C	-0.69075300	2.99873100	2.88591100

H	-1.06552200	3.96372700	3.21262700
C	-0.71334100	1.90369300	3.75014700
H	-1.11078900	2.00531600	4.75551000
C	1.90023100	-3.03290400	1.50334900
H	2.54972200	-3.87550600	1.69619000
Sn	-1.35837000	-1.36645200	-0.78491200
Br	-2.91508100	0.35455600	0.13712500
B	0.97020800	1.57164300	-0.27911700
Br	0.11340700	2.65201600	-1.66683600
C	2.26452200	0.82455200	-0.67137700
C	3.23012300	0.53871200	0.31394700
C	2.53679000	0.41617500	-1.99212200
C	4.40326300	-0.14255300	0.00174600
H	3.05669700	0.86034300	1.33477600
C	3.69638100	-0.28636900	-2.30601300
H	1.82247400	0.64923800	-2.77419000
C	4.63279600	-0.56781900	-1.30795900
H	5.13328400	-0.34835400	0.77859600
H	3.87725800	-0.60702400	-3.32774800
H	5.54161400	-1.11035200	-1.55217600
C	-0.84663600	-0.84106600	-2.78419900
H	-1.61525200	-1.21337100	-3.46542200
H	-0.76782400	0.24068800	-2.88709400
H	0.11429300	-1.29323200	-3.04286500
C	-2.42184800	-3.21299400	-0.73312300
H	-3.34368300	-3.13897700	-1.31441100
H	-1.80753400	-4.01516100	-1.15303800
H	-2.67531500	-3.47061200	0.29726800

9) **TSA[2-3]**

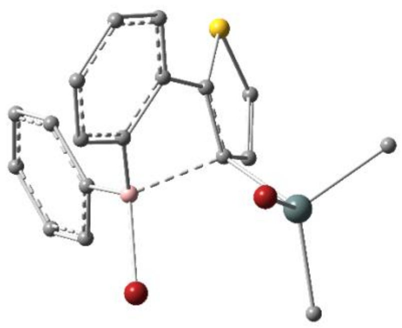

Number of imaginary frequencies = 1 (115.0 cm^{-1})

E_{total} = -6265.597940 a.u

$G_{correction}$ = 0.239991 a.u

Cartesian coordinates:

S	1.49354500	0.77949400	2.96348800
C	0.47123000	1.59013500	-0.70618800
C	0.37793700	-0.48400900	0.95415100
C	0.32095200	1.95305800	0.65551700
C	0.19790300	2.55482700	-1.68056000
H	0.27412100	2.29058300	-2.73068400
C	1.03315800	-1.48045000	1.78847100
H	1.03473600	-2.54133300	1.56252000
C	-0.02670400	3.25359600	1.04306100
H	-0.13902400	3.50738200	2.09327200
C	0.56825600	0.80416000	1.50328900
C	-0.16026500	3.84744600	-1.30283900
H	-0.35719900	4.59705200	-2.06362000
C	-0.26650600	4.19821000	0.05205800
H	-0.55332000	5.20862500	0.32643700
C	1.63821600	-0.96339900	2.88961400
H	2.17880700	-1.48387500	3.66874700
Sn	-1.68885800	-1.06250200	0.57557100
Br	-2.94554400	0.70496800	-0.63617600
B	1.03503700	0.12596900	-0.90132200

Br	0.16392100	-0.91550400	-2.39459500
C	2.57811500	-0.17544100	-0.71770500
C	3.44078900	0.80563200	-0.19632500
C	3.13388000	-1.43315500	-1.01007600
C	4.79223700	0.54210300	0.03004100
H	3.05137900	1.79423400	0.02641500
C	4.48143000	-1.70506500	-0.78497800
H	2.49492700	-2.20515500	-1.42803900
C	5.31762900	-0.71676200	-0.26090100
H	5.43537700	1.32239900	0.42754200
H	4.88223300	-2.68669600	-1.02268700
H	6.36979300	-0.92456300	-0.08814100
C	-1.79371500	-2.96470500	-0.36617600
H	-2.28637500	-3.67755400	0.29997800
H	-2.35826500	-2.88169700	-1.29559100
H	-0.78732400	-3.30909900	-0.60823700
C	-2.52445200	-1.04450900	2.53739500
H	-3.57712300	-1.33559600	2.52075400
H	-1.97085700	-1.73473900	3.17994500
H	-2.44459000	-0.03791200	2.95354000

10) Intermediate **A3**

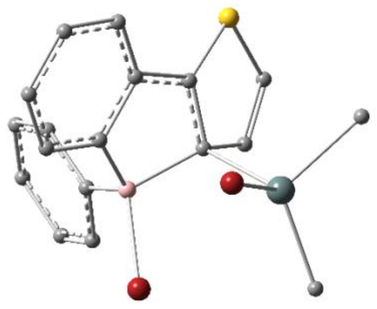

(Hydrogens are omitted for clarity)

Number of imaginary frequencies = 0

E_{total} = -6265.601470 a.u

$G_{correction}$ = 0.239522 a.u

Cartesian coordinates:

S	0.54292700	0.63965300	3.23939800
C	0.64334500	1.58522500	-0.61287400
C	0.43721100	-0.44978400	0.84181100
C	0.24348100	1.95848800	0.69984800
C	0.60801000	2.56291700	-1.60783300
H	0.88390300	2.30167200	-2.62480700
C	0.84170400	-1.49483300	1.78352100
H	1.05694200	-2.51299800	1.47933400
C	-0.14397200	3.26694600	1.02706300
H	-0.45093900	3.52375000	2.03708700
C	0.30430400	0.79239800	1.54232800
C	0.22047600	3.86486700	-1.29283500
H	0.20437400	4.62449700	-2.06922500
C	-0.15072600	4.21837000	0.01664900
H	-0.45420800	5.23768000	0.23342900
C	0.89072800	-1.07968100	3.07368900
H	1.11926300	-1.65503000	3.96089000
Sn	-1.69698000	-1.05370800	0.37643000
Br	-2.81353200	0.77139900	-0.87944600
B	1.13334200	0.06033700	-0.64364600
Br	0.38908800	-0.92923300	-2.30722600
C	2.70992300	-0.18900400	-0.46388300
C	3.51564000	0.77196400	0.17104300
C	3.32254300	-1.39866500	-0.82954400
C	4.86624100	0.53753300	0.43283300
H	3.08267200	1.72488900	0.46161200
C	4.67195300	-1.64171900	-0.57290400
H	2.73169200	-2.15482500	-1.33805700
C	5.45123700	-0.67375300	0.06263700
H	5.46340200	1.30467600	0.91880900
H	5.11704500	-2.58646200	-0.87414700

H	6.50328500	-0.85866800	0.26062100
C	-1.75443500	-2.96726900	-0.54340600
H	-2.33028400	-3.65282000	0.08369400
H	-2.21924100	-2.87703800	-1.52563700
H	-0.73737900	-3.33520800	-0.68077800
C	-2.68307200	-1.04350600	2.26886700
H	-3.71391800	-1.38302200	2.13970100
H	-2.17336600	-1.71337600	2.96515000
H	-2.69982500	-0.03721800	2.69016000

11) **TSA[3-4]**

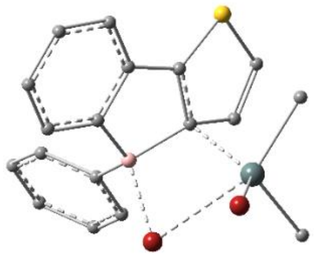

(Hydrogens are omitted for clarity)

Number of imaginary frequencies = 1 (47.3 cm^{-1})

E_{total} = -6265.594078 a.u

$G_{correction}$ = 0.241266 a.u

Cartesian coordinates:

S	0.38323200	2.01886200	2.94155600
C	1.11140500	1.43675700	-0.94562000
C	0.64498700	0.13790800	1.13397400
C	0.63114200	2.30362600	0.07195900
C	1.29133800	1.96244700	-2.22321600
H	1.63955200	1.32139500	-3.02803500
C	0.74375300	-0.50353100	2.42983000
H	0.91008400	-1.56558500	2.56485500
C	0.36388100	3.65632400	-0.16267200
H	-0.00269000	4.30097400	0.63140200

C	0.48154000	1.53461400	1.29341600
C	1.02642100	3.31356300	-2.46957200
H	1.17550300	3.71918400	-3.46598600
C	0.56920900	4.15564400	-1.44689300
H	0.36645500	5.20073500	-1.65903100
C	0.58051900	0.35622800	3.47421300
H	0.56541500	0.13636600	4.53313000
Sn	-1.67596700	-0.65571500	0.53657100
B	1.37323000	-0.01954500	-0.33325800
Br	0.11275400	-1.40396100	-1.49433500
C	2.83865600	-0.61868500	-0.28863100
C	3.94725100	0.15319300	-0.67076100
C	3.08908400	-1.91811300	0.18818800
C	5.24878200	-0.34605500	-0.58096400
H	3.78837600	1.16071500	-1.04299100
C	4.38198900	-2.42640800	0.27856800
H	2.25012500	-2.54914400	0.47185000
C	5.47026200	-1.63773600	-0.10598500
H	6.08803400	0.27304500	-0.88591700
H	4.54499600	-3.43685600	0.64346500
H	6.48057200	-2.03104700	-0.03848700
C	-1.69939600	-2.67740800	1.20223700
H	-2.29821200	-3.26754000	0.50575700
H	-0.68273700	-3.07173400	1.21008600
H	-2.13314700	-2.73711100	2.20337400
Br	-3.30664300	-0.22411400	-1.35124100
C	-2.65124400	0.68452600	1.88897700
H	-2.21321500	0.64304700	2.88582100
H	-2.61518100	1.70513400	1.50805100
H	-3.69477000	0.36132800	1.92837800

12) Intermediate **A4**

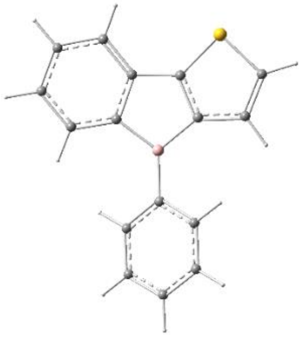

Number of imaginary frequencies = 0

E_{total} = -1039.468174 a.u

$G_{correction}$ = 0.181135 a.u

Cartesian coordinates:

S	-3.02311400	-1.93769400	0.12459000
B	0.45084300	-0.01866100	-0.02737600
C	-0.51975600	1.23662900	-0.05778400
C	-0.51919500	-1.24165800	0.00016400
C	-1.86013000	0.74978900	-0.01748000
C	-0.31836100	2.60615600	-0.15622200
H	0.68746100	3.00993900	-0.22588600
C	-0.53349400	-2.66936600	0.07185700
H	0.35249900	-3.29385200	0.08334000
C	-2.94831900	1.60437400	-0.04383600
H	-3.96456400	1.22215400	-0.01183700
C	-1.80729700	-0.72014000	0.02725500
C	-1.41722200	3.48715900	-0.18170700
H	-1.25131700	4.55764800	-0.25294700
C	-2.71304600	2.98985600	-0.12102500
H	-3.55633300	3.67371100	-0.14166700
C	-1.79803900	-3.19077000	0.13592100
H	-2.10010000	-4.22697900	0.19318800
C	1.99357300	-0.03695200	-0.00442900
C	2.70589600	-1.14905600	-0.49910300
C	2.74454400	1.03879100	0.51221100

C	4.09763400	-1.17965900	-0.49844600
H	2.15355800	-1.99004300	-0.90762800
C	4.13634400	1.00552800	0.53872700
C	4.81510600	-0.10191900	0.02573100
H	4.62508100	-2.04103500	-0.89728900
H	4.69384800	1.83992800	0.95370700
H	5.90087900	-0.12647100	0.03707000
H	2.22516200	1.89883300	0.92237900

13) Dimethyltin dibromide

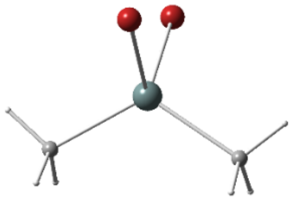

Number of imaginary frequencies = 0

E_{total} = -5226.151289 a.u

$G_{correction}$ = 0.035979 a.u

Cartesian coordinates:

Sn	-0.00007300	0.59184300	-0.00010300
Br	2.01314000	-0.87128400	0.00062100
C	0.00154800	1.67909400	-1.82072600
H	-0.88975100	2.30814400	-1.87641100
H	0.89445600	2.30592300	-1.87561900
H	0.00100600	0.98139100	-2.65969400
C	0.00010400	1.68316400	1.81803600
H	0.89183500	2.31188200	1.87095000
H	-0.89232000	2.31077400	1.87219800
H	0.00114500	0.98799600	2.65910200
Br	-2.01350100	-0.87076800	0.00025800

14) **TSA[4-5]**

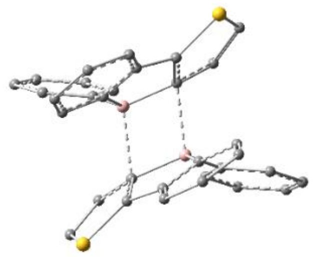

(Hydrogens are omitted for clarity)

Number of imaginary frequencies = 1 (297.6 cm^{-1})

E_{total} = -2078.952578 a.u

$G_{correction}$ = 0.386610 a.u

Cartesian coordinates:

S	1.02567400	1.55208000	3.39132600
S	1.02554500	-1.55184200	-3.39123400
B	-0.31619000	0.94005700	-0.58308200
C	1.19930700	1.43540200	-0.75570800
C	-0.29832700	-0.48586100	-1.39564700
C	1.87726800	0.55296600	-1.62960100
C	1.92562800	2.48080900	-0.18571800
H	1.45164500	3.15373900	0.52252100
C	-0.29809500	0.48582100	1.39584900
C	-1.20134700	-1.39175000	-2.06974300
H	-2.20764900	-1.60252500	-1.73602200
C	-1.20130500	1.39139400	2.07004600
H	-2.20759900	1.60217100	1.73619100
C	1.20027600	-1.43471100	0.75532000
C	3.22993500	0.70618000	-1.94438200
H	3.73104900	0.00603100	-2.60635900
C	-0.63676700	2.05012700	3.11625400
H	-1.07638400	2.80858300	3.74829300
C	0.94580900	0.47017900	2.05692200
C	1.92694100	-2.47975600	0.18510800
H	1.45310700	-3.15282200	-0.52310000

C	3.27845900	-2.64842100	0.49820000
H	3.83545600	-3.46949600	0.05653900
C	0.94546700	-0.46981800	-2.05695600
C	3.27701900	2.65001500	-0.49906300
H	3.83373900	3.47137900	-0.05759400
C	1.87798600	-0.55213000	1.62925700
C	3.92587900	1.76780400	-1.37196200
H	4.97854400	1.90829600	-1.59703900
C	3.23076900	-0.70481000	1.94379800
H	3.73168500	-0.00455700	2.60581700
C	3.92708500	-1.76604200	1.37110100
H	4.97984900	-1.90610700	1.59598300
C	-0.63671300	-2.05040900	-3.11591900
H	-1.07618300	-2.80899100	-3.74790800
B	-0.31547800	-0.93999800	0.58301700
C	-1.55717900	1.89244500	-0.80321800
C	-2.85373200	1.40882600	-1.05216400
C	-1.39491300	3.28865600	-0.75722400
C	-3.93877000	2.26862000	-1.21606800
H	-3.02398400	0.34103400	-1.11492500
C	-2.47129800	4.15758700	-0.92800100
H	-0.40668900	3.70220700	-0.59150300
C	-3.75166500	3.64998000	-1.15099200
H	-4.92829900	1.86087100	-1.40225600
H	-2.31085700	5.23127100	-0.88842800
H	-4.59341000	4.32364700	-1.28186300
C	-1.55588100	-1.89304500	0.80333900
C	-1.39276000	-3.28914200	0.75773700
C	-2.85270800	-1.41017000	1.05233600
C	-2.46859800	-4.15871400	0.92873200
H	-0.40428300	-3.70208200	0.59220500
C	-3.93720900	-2.27059500	1.21647100
H	-3.02358500	-0.34246600	1.11509900

C	-3.74927300	-3.65186000	1.15163000
H	-2.30747600	-5.23230700	0.88943100
H	-4.92696400	-1.86341400	1.40270400
H	-4.59060000	-4.32601400	1.28267600

15) Diboracycle **A5**

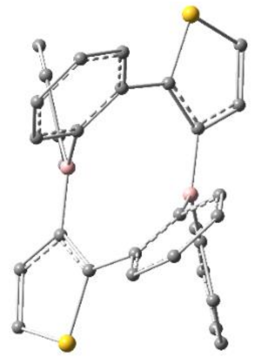

(Hydrogens are omitted for clarity)

Number of imaginary frequencies = 0

E_{total} = -2078.995618 a.u

$G_{correction}$ = 0.389402 a.u

Cartesian coordinates:

S	3.71431600	0.31409300	1.59118500
S	-3.71440000	0.31135400	-1.59131300
B	-0.38128400	-0.05230700	1.38305500
C	-1.02177200	1.35807900	1.12385100
C	-1.15039600	-0.15628300	-1.62085000
C	-1.91445200	1.63756900	0.06082100
C	-0.76659700	2.37938600	2.05313300
H	-0.05535000	2.19268900	2.85249800
C	1.15056600	-0.15505700	1.62107500
C	-1.76670100	-1.04436300	-2.57550600
H	-1.19755900	-1.73657100	-3.18356200
C	1.76759600	-1.04287700	2.57553500
H	1.19897500	-1.73546600	3.18364500

C	1.02094000	1.35826800	-1.12379300
C	-2.56211800	2.88080800	-0.00577100
H	-3.22496600	3.08957000	-0.84005800
C	3.11863300	-0.89809200	2.68391600
H	3.80150000	-1.42323100	3.33687300
C	2.10608800	0.65426800	1.00771300
C	0.76503800	2.37895400	-2.05357000
H	0.05395700	2.19135200	-2.85286900
C	1.41250600	3.61054000	-1.98667200
H	1.20726200	4.37475200	-2.73023300
C	-2.10643400	0.65245800	-1.00754300
C	-1.41498600	3.61046400	1.98572400
H	-1.21024600	4.37516400	2.72892100
C	1.91337500	1.63893300	-0.06089700
C	-2.32413700	3.85775600	0.95575700
H	-2.82722300	4.81743400	0.88729500
C	2.56011100	2.88267700	0.00520800
H	3.22278000	3.09227100	0.83943000
C	2.32140600	3.85901100	-0.95675500
H	2.82370600	4.81913000	-0.88870000
C	-3.11781000	-0.90031500	-2.68411200
H	-3.80024900	-1.42578600	-3.33725000
B	0.38137300	-0.05269400	-1.38233300
C	-1.30311700	-1.30084800	1.54923800
C	-0.80117700	-2.61484100	1.45490400
C	-2.68615500	-1.15416600	1.77734400
C	-1.63634200	-3.72407400	1.56708800
H	0.25440600	-2.76593400	1.25972200
C	-3.52571100	-2.25704700	1.90507100
H	-3.10369600	-0.15555300	1.85528400
C	-3.00200800	-3.54700900	1.79550000
H	-1.22462000	-4.72505100	1.47523200
H	-4.58731200	-2.11417200	2.08358700

H	-3.65575400	-4.40970300	1.88678400
C	1.30395200	-1.30067400	-1.54857200
C	2.68690900	-1.15312500	-1.77662200
C	0.80278000	-2.61498300	-1.45458500
C	3.52713800	-2.25546900	-1.90446300
H	3.10378600	-0.15422300	-1.85443600
C	1.63861800	-3.72369900	-1.56692100
H	-0.25273000	-2.76674500	-1.25957300
C	3.00419300	-3.54577100	-1.79514900
H	4.58866800	-2.11194900	-2.08287200
H	1.22748000	-4.72494400	-1.47536000
H	3.65848300	-4.40804500	-1.88651300

4.1.3 Mechanism for the azide addition

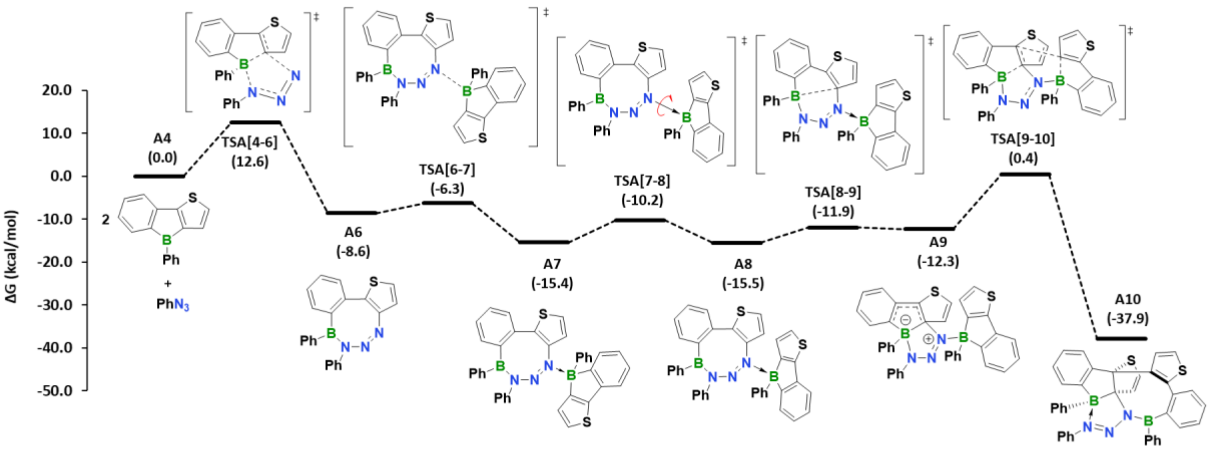

Figure S40. Computed mechanism for the formation of **A10** from the reaction of benzothienoborole with phenyl azide.

Note: Details for the benzothienoborole are provided above in the mechanism computed for the boron-tin exchange reaction.

16) Phenyl azide

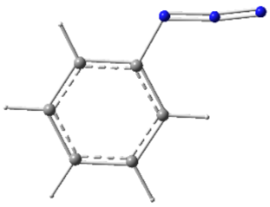

Number of imaginary frequencies = 0

E_{total} = -395.871892 a.u

$G_{correction}$ = 0.071897 a.u

Cartesian coordinates:

N	1.46877800	0.89171500	-0.00013900
N	2.41035600	0.09012100	0.00015600
N	3.36303600	-0.53837100	0.00008800
C	0.14812300	0.36792400	-0.00018900
C	-0.88549400	1.31014800	-0.00003200
C	-0.14542100	-1.00142700	-0.00012000

C	-2.20932400	0.88058200	0.00012200
H	-0.63311800	2.36465400	0.00005600
C	-1.47470400	-1.41746200	0.00001300
H	0.65705100	-1.73273100	-0.00011000
C	-2.51108400	-0.48271400	0.00002800
H	-3.00843400	1.61518200	0.00027800
H	-1.69902500	-2.47961100	0.00005700
H	-3.54423800	-0.81405900	0.00005000

17) **TSA[4-6]**

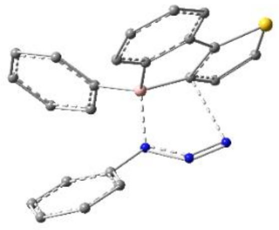

(Hydrogens are omitted for clarity)

Number of imaginary frequencies = 1 (124.0 cm^{-1})

E_{total} = -1435.348203 a.u

$G_{correction}$ = 0.275979 a.u

Cartesian coordinates:

S	4.23929800	0.33182500	-0.45024400
N	1.33635500	-0.82456500	-2.34794000
N	-0.62405600	-0.25420100	-1.29053000
N	0.20208700	-0.84652900	-2.10425000
C	1.67213000	0.81759200	-0.62093100
C	2.33370900	1.83986100	-1.38195600
H	1.81853900	2.63685100	-1.90508700
C	0.62896200	-0.80706500	0.94706500
C	3.69172000	1.70939200	-1.39622400
H	4.42223700	2.31951500	-1.90922700
C	2.60718700	-0.06139000	-0.04726300

C	-0.13066900	-1.67250600	1.72629500
H	-1.20519900	-1.53338500	1.80696800
C	0.48269600	-2.72985900	2.41839400
H	-0.11922100	-3.39285600	3.03332200
C	2.02711000	-1.04864400	0.85381700
C	2.64712200	-2.09465800	1.53562100
H	3.71834200	-2.25705100	1.45413500
C	1.85971600	-2.93627900	2.32689200
H	2.32229100	-3.75544100	2.86884800
B	0.22367000	0.44860700	-0.01180100
C	-0.66536600	1.64723400	0.54793100
C	-0.98603700	2.73987400	-0.27843300
C	-1.15745700	1.66744500	1.86040600
C	-1.77174900	3.79670600	0.17567300
H	-0.62738600	2.74941000	-1.30521000
C	-1.94075700	2.72467400	2.32947900
H	-0.92058600	0.84379300	2.52643800
C	-2.25481900	3.79029000	1.48731500
H	-2.00754800	4.62506400	-0.48682300
H	-2.30892500	2.71361200	3.35164700
H	-2.86804900	4.61121600	1.84748000
C	-1.91098200	-0.86013900	-1.14651100
C	-3.01090700	-0.04498400	-0.86930300
C	-2.05698100	-2.24891300	-1.22284600
C	-4.25984600	-0.63179300	-0.67289800
H	-2.88300700	1.02727500	-0.80631400
C	-3.31424200	-2.82075000	-1.04591800
H	-1.18562900	-2.86946700	-1.40371300
C	-4.41858300	-2.01501100	-0.76461600
H	-5.11411200	0.00137500	-0.45570300
H	-3.42554500	-3.89845700	-1.11090900
H	-5.39575900	-2.46309000	-0.61583500

18) **A6**

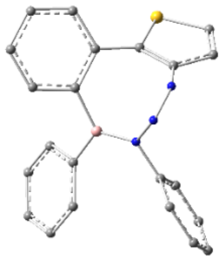

(Hydrogens are omitted for clarity)

Number of imaginary frequencies = 0

E_{total} = -1435.381992 a.u

$G_{correction}$ = 0.277712 a.u

Cartesian coordinates:

S	-3.00999400	-1.34187100	1.83834100
N	-1.00349000	-2.14014500	-1.46554800
N	0.62926600	-0.60514500	-0.86334600
N	-0.03895500	-1.47997700	-1.85503800
C	-1.55391100	-2.04273000	-0.15823300
C	-1.65975600	-3.20227700	0.67266800
H	-1.19688800	-4.15000000	0.42864800
C	-1.33826600	1.08954400	-0.81941200
C	-2.38954700	-2.96328600	1.80019100
H	-2.60024600	-3.64326800	2.61304900
C	-2.24166700	-0.94731300	0.31682100
C	-1.59644100	2.32107800	-1.44379600
H	-0.75684400	2.91943900	-1.78488000
C	-2.89168600	2.80365900	-1.61223900
H	-3.05840300	3.75470300	-2.10867900
C	-2.44484500	0.34858500	-0.34209200
C	-3.74775700	0.85411000	-0.48161500
H	-4.58426900	0.27311800	-0.10611600
C	-3.97170900	2.07058900	-1.11756000
H	-4.98518300	2.44348600	-1.22915100

B	0.14896300	0.67660000	-0.52034500
C	1.91907600	-1.14407900	-0.58172900
C	2.78577100	-1.51914200	-1.61448000
C	2.30010000	-1.31331000	0.75211600
C	4.04029300	-2.04070500	-1.30336500
H	2.47204100	-1.39469200	-2.64408200
C	3.56377900	-1.81623900	1.05278300
H	1.60615700	-1.03219100	1.53594800
C	4.43674600	-2.18333500	0.02751600
H	4.71382700	-2.32693600	-2.10528300
H	3.86223700	-1.92969900	2.09029600
H	5.41835600	-2.58173700	0.26373500
C	1.07940700	1.73017500	0.17810700
C	2.44149600	1.89240500	-0.13824000
C	0.53060900	2.59516400	1.14308000
C	3.21777700	2.86836500	0.48195400
H	2.90036500	1.24953100	-0.88159600
C	1.30750200	3.55732900	1.78649800
H	-0.52316700	2.50907500	1.39131500
C	2.65499200	3.69787200	1.45403900
H	4.26362000	2.98002900	0.21110200
H	0.86127700	4.20180500	2.53828200
H	3.26270700	4.45237400	1.94512600

19) **TSA[6-7]**

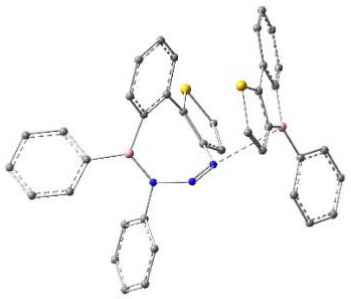

(Hydrogens are omitted for clarity)

Number of imaginary frequencies = 1 (23.7 cm^{-1})

E_{total} = -2474.875442 a.u

$G_{correction}$ = 0.481456 a.u

Cartesian coordinates:

S	-0.53585500	-0.74496300	-3.22004600
S	-2.17620700	-2.33086600	3.06627400
N	-0.23669500	1.07797200	0.25462400
B	-3.06444200	0.67599200	0.62392800
N	2.04771400	0.72676200	0.37061200
N	0.75314500	1.08178700	0.98594800
C	-3.47084100	-0.53955400	-0.31832200
C	-2.55876100	-0.02268100	1.92641000
C	-3.32291700	-1.74019100	0.43717300
C	-3.90658700	-0.63766400	-1.63207400
H	-3.97458000	0.24947000	-2.25367200
C	-0.26005700	0.67936700	-1.10239100
C	-1.95816700	0.25351600	3.19208000
H	-1.71876600	1.24578400	3.55557700
C	-0.82248000	1.55331100	-2.08744700
H	-1.07846300	2.58473900	-1.88349900
C	1.38884900	-1.77362200	0.14312100
C	-3.67656500	-2.97505800	-0.07929000
H	-3.57422700	-3.87946300	0.51308300
C	-0.99050000	0.92944100	-3.28840300
H	-1.35290500	1.35481100	-4.21319400
C	-0.06201000	-0.61391200	-1.54288500
C	1.63073200	-2.92452600	0.91093500
H	2.49076500	-2.93840400	1.57376800
C	0.81506100	-4.05008900	0.82729300
H	1.01757100	-4.91718000	1.44840000
C	-2.74335100	-1.38657800	1.74040300
C	-4.24069100	-1.89204200	-2.17982800

H	-4.57841600	-1.96020700	-3.20932600
C	0.28712000	-1.79292100	-0.74433100
C	-4.14521400	-3.04161800	-1.40413300
H	-4.41936000	-4.00388900	-1.82661400
C	-0.50074000	-2.94867600	-0.86612300
H	-1.34103100	-2.94637800	-1.54968500
C	-0.24551800	-4.06590100	-0.07935900
H	-0.88038200	-4.94207100	-0.16838400
C	-1.68988500	-0.87801100	3.91549800
H	-1.23496600	-0.96235300	4.89236400
B	2.44510300	-0.61151300	0.16393800
C	2.87880900	1.88511900	0.35777500
C	3.00979000	2.69113800	1.49445900
C	3.55030400	2.21960300	-0.82124100
C	3.83097300	3.81633900	1.44876800
H	2.47409500	2.42703700	2.39837400
C	4.38445000	3.33473600	-0.85067200
H	3.42242500	1.59119300	-1.69510900
C	4.52627300	4.13797800	0.28182200
H	3.93527600	4.43785500	2.33287900
H	4.91644800	3.58107100	-1.76430100
H	5.17087500	5.01079300	0.25428100
C	3.95460100	-0.98295000	-0.05115100
C	5.00992600	-0.36833500	0.64873100
C	4.28602200	-2.01173800	-0.95234000
C	6.33253700	-0.75916200	0.45443800
H	4.79443600	0.42435700	1.35709200
C	5.60966200	-2.39025100	-1.17145700
H	3.49056000	-2.52102400	-1.48852900
C	6.63725900	-1.76517500	-0.46462600
H	7.12719000	-0.27635100	1.01575200
H	5.83871600	-3.17723000	-1.88411300
H	7.66945100	-2.06333000	-0.62427900

C	-3.26416200	2.18307800	0.33541300
C	-4.22260900	2.63038000	-0.59473900
C	-2.48228400	3.16136600	0.98290000
C	-4.39004900	3.98576100	-0.87424500
H	-4.85768600	1.90424600	-1.09126800
C	-2.62985600	4.51513900	0.69595900
H	-1.72375000	2.84324600	1.68936500
C	-3.58611000	4.93054900	-0.23532900
H	-5.14085600	4.30552500	-1.59095800
H	-2.00418400	5.24891500	1.19568200
H	-3.70579200	5.98703600	-0.45766900

20) **A7**

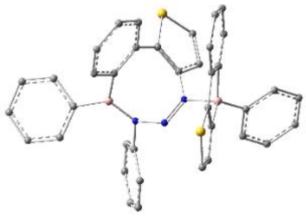

(Hydrogens are omitted for clarity)

Number of imaginary frequencies = 0

E_{total} = -2474.891428 a.u

$G_{correction}$ = 0.484055 a.u

Cartesian coordinates:

S	-0.07896700	-1.42080700	3.55506200
S	2.83007300	-0.88217200	-3.89820200
N	0.57169100	0.54026700	0.21022300
B	2.14420100	0.68472500	-0.23693200
N	-1.64464300	0.62574500	-0.26264300
N	-0.29510500	0.78151900	-0.65514900
C	2.82178100	-0.74434700	0.15907400
C	2.19959200	0.59838600	-1.84523200
C	3.09854800	-1.46358300	-1.03616900

C	3.13754300	-1.33710500	1.37664000
H	2.94122100	-0.81022300	2.30703000
C	0.27605900	0.12767600	1.55491000
C	1.88049500	1.36359800	-3.00482900
H	1.46591000	2.36569900	-2.98012200
C	0.61633500	0.91678600	2.69559600
H	0.96131800	1.93918300	2.63632900
C	-1.36867800	-1.95784300	-0.14277200
C	3.65254700	-2.74233500	-1.00288100
H	3.85084500	-3.28536600	-1.92288000
C	0.45036800	0.20869700	3.85213300
H	0.60062000	0.55208700	4.86535300
C	-0.10701700	-1.16059400	1.83502700
C	-1.60482100	-2.96292200	-1.09397400
H	-2.37310000	-2.80762600	-1.84549500
C	-0.87897700	-4.15213400	-1.09030400
H	-1.06478000	-4.90296300	-1.85195900
C	2.72361800	-0.63533900	-2.17924900
C	3.71168100	-2.61767400	1.41941200
H	3.96697300	-3.06614600	2.37554600
C	-0.39526000	-2.20652500	0.84961600
C	3.95747100	-3.31543500	0.23632600
H	4.39630700	-4.30853700	0.27513100
C	0.31039300	-3.41617300	0.87565500
H	1.07631600	-3.56901800	1.62660500
C	0.07885500	-4.37919100	-0.10097900
H	0.65190300	-5.30069000	-0.09356700
C	2.15687700	0.70787700	-4.17765400
H	2.01792300	1.05505600	-5.19223900
B	-2.25618000	-0.66286200	-0.17177900
C	-2.31757900	1.88407400	-0.44194100
C	-2.17790200	2.60501300	-1.63155100
C	-3.09241700	2.37763700	0.60680800

C	-2.84534200	3.82046900	-1.77187900
H	-1.55356900	2.21206800	-2.42572600
C	-3.77071900	3.58488400	0.44738700
H	-3.16933900	1.80515600	1.52385500
C	-3.64820000	4.30887000	-0.73902300
H	-2.74279100	4.38269600	-2.69456800
H	-4.38762500	3.96289000	1.25630100
H	-4.17108300	5.25247100	-0.85687800
C	-3.81177100	-0.82130900	-0.15821100
C	-4.70161100	-0.04057500	-0.92075200
C	-4.36185000	-1.84939500	0.63167900
C	-6.07458100	-0.27178600	-0.88857700
H	-4.31932200	0.75078100	-1.55518400
C	-5.73698400	-2.06676400	0.69009000
H	-3.69732200	-2.48599300	1.20819000
C	-6.59728000	-1.27797400	-0.07392100
H	-6.73842500	0.33549400	-1.49659100
H	-6.13558700	-2.85629000	1.32007600
H	-7.66903100	-1.45052000	-0.04119000
C	2.64602400	2.03098100	0.48262500
C	3.80475700	2.06949900	1.27187700
C	1.92508400	3.22910200	0.33123000
C	4.23021800	3.25090000	1.88194100
H	4.37987000	1.15889500	1.40685700
C	2.33621700	4.41240900	0.94420400
H	1.01851600	3.23590600	-0.27085400
C	3.49372900	4.42560100	1.72571500
H	5.13427000	3.25335000	2.48486100
H	1.75702400	5.32252200	0.81329300
H	3.81835500	5.34399800	2.20633400

21) **TSA[7-8]**

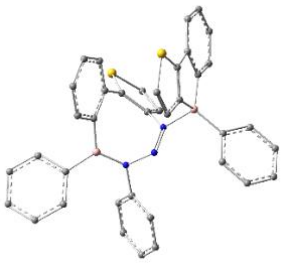

(Hydrogens are omitted for clarity)

Number of imaginary frequencies = 1 (18.5 cm^{-1})

E_{total} = -2474.882960 a.u

$G_{correction}$ = 0.484731 a.u

Cartesian coordinates:

S	-0.15844300	-1.95153300	-3.15965000
S	-3.16196700	-2.27223700	2.53919800
N	-0.49747800	0.67894700	-0.25388200
B	-2.06380500	0.92709900	0.31176900
N	1.73869900	0.64642800	0.08649000
N	0.43899200	1.03032400	0.48865200
C	-3.09515000	0.17242800	-0.70180600
C	-2.11690100	-0.08826200	1.57276600
C	-3.59849600	-0.99655800	-0.06207000
C	-3.54640000	0.44375000	-1.98933700
H	-3.19216300	1.32353500	-2.51892300
C	-0.29190000	-0.03048600	-1.48621600
C	-1.51613200	-0.28746300	2.85071300
H	-0.77545700	0.36991400	3.29273100
C	-0.48820300	0.57907700	-2.75960100
H	-0.66878000	1.63651300	-2.89937700
C	1.19987400	-1.87935200	0.53114100
C	-4.49398800	-1.85679400	-0.69388700
H	-4.86353400	-2.74295800	-0.18467300
C	-0.42251100	-0.34349900	-3.76367900

H	-0.52760200	-0.17581600	-4.82566600
C	-0.09636400	-1.38490300	-1.51314200
C	1.41869800	-2.70115900	1.64818000
H	2.24009100	-2.46955300	2.31953900
C	0.61397600	-3.80805900	1.90733200
H	0.79229300	-4.41348200	2.79042200
C	-3.00761900	-1.09675500	1.26738400
C	-4.44830200	-0.41475700	-2.63811900
H	-4.78287800	-0.18619000	-3.64596100
C	0.15083000	-2.23567400	-0.34712300
C	-4.91499500	-1.55895700	-1.99447900
H	-5.61240800	-2.22036900	-2.50053400
C	-0.63992700	-3.36431600	-0.10471200
H	-1.45544400	-3.59558900	-0.78206600
C	-0.41436200	-4.14286300	1.02611200
H	-1.04854500	-5.00020200	1.22556500
C	-1.97211400	-1.41256300	3.48963000
H	-1.70127000	-1.78578000	4.46753600
B	2.20060600	-0.69718300	0.26151500
C	2.54617700	1.81945400	-0.10730000
C	2.51066000	2.87829500	0.80417900
C	3.34513200	1.88805100	-1.24912600
C	3.30769500	3.99873700	0.57669300
H	1.86054000	2.82393600	1.66876600
C	4.15201800	3.00496400	-1.45561100
H	3.33898700	1.06271900	-1.95145400
C	4.13586700	4.06221000	-0.54513300
H	3.28214300	4.82264600	1.28261100
H	4.78566600	3.05139000	-2.33547100
H	4.75830200	4.93514800	-0.71301000
C	3.73219600	-1.01407500	0.25452700
C	4.70927800	-0.19242700	0.84803700
C	4.16175800	-2.22178300	-0.32746900

C	6.05298700	-0.55787900	0.85346400
H	4.41702900	0.74066700	1.31705400
C	5.50879000	-2.57818200	-0.35218500
H	3.42507400	-2.88841500	-0.76589400
C	6.45786400	-1.74629400	0.24228200
H	6.78617400	0.08607800	1.32979400
H	5.81622000	-3.50738800	-0.82248400
H	7.50743800	-2.02528400	0.23639100
C	-2.22939500	2.50201200	0.57926500
C	-3.11268500	3.29292400	-0.17199400
C	-1.49805800	3.15201100	1.59101400
C	-3.24688000	4.66475600	0.05470900
H	-3.72239400	2.82540500	-0.93724900
C	-1.62050600	4.52042200	1.82388200
H	-0.82342800	2.57296300	2.21274900
C	-2.49534300	5.28604600	1.05023700
H	-3.94181200	5.24604900	-0.54545500
H	-1.04222400	4.98983600	2.61553700
H	-2.59636200	6.35262500	1.23017400

22) **A8**

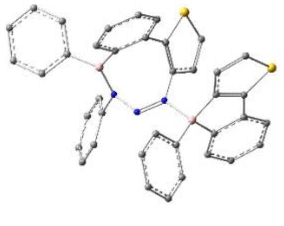

(Hydrogens are omitted for clarity)

Number of imaginary frequencies = 0

E_{total} = -2474.889768 a.u

$G_{correction}$ = 0.483249 a.u

Cartesian coordinates:

S	0.14063200	-2.42927800	2.72495300

S	4.72795200	-1.98816600	1.03039400
N	0.54401000	0.34045300	-0.04961600
B	2.04688000	0.51078600	-0.69164800
N	-1.68900700	0.71346000	-0.05703900
N	-0.41062600	0.88565900	-0.64157700
C	2.76719900	1.50237800	0.38082900
C	2.85059600	-0.85662800	-0.38573500
C	3.76698500	0.78044000	1.09141700
C	2.51279800	2.82258200	0.73308700
H	1.73338500	3.38160600	0.21970100
C	0.39405800	-0.41836900	1.16930300
C	2.90947000	-2.21587200	-0.81233000
H	2.28349900	-2.64117800	-1.58788600
C	0.92269500	0.01584800	2.42215900
H	1.34249000	0.99668000	2.59114600
C	-1.70024400	-1.81065700	-0.71227900
C	4.50854300	1.38516500	2.10496800
H	5.26988700	0.82910200	2.64505400
C	0.83795800	-0.97110200	3.36183400
H	1.14841400	-0.92741600	4.39553300
C	-0.07333500	-1.70829900	1.15483600
C	-2.21176700	-2.49768700	-1.82572600
H	-3.04682600	-2.06787900	-2.37067000
C	-1.68636800	-3.72210200	-2.23224100
H	-2.09136200	-4.22366100	-3.10558700
C	3.78471200	-0.59241500	0.59972400
C	3.25560000	3.43813500	1.75300600
H	3.06200300	4.47321900	2.01897900
C	-0.64049200	-2.41374600	0.00295300
C	4.24487500	2.72098200	2.42889900
H	4.81417800	3.20256100	3.21881300
C	-0.13823100	-3.66503400	-0.38194600
H	0.68221500	-4.09958800	0.17883400

C	-0.65435900	-4.31285100	-1.50016200
H	-0.24943400	-5.27351900	-1.80260100
C	3.85586600	-2.94996800	-0.14225200
H	4.12011400	-3.98981600	-0.27749000
B	-2.42111800	-0.49760300	-0.23199300
C	-2.21415900	1.99776900	0.31530400
C	-2.14588300	3.08275500	-0.56327900
C	-2.77119700	2.14022900	1.58601500
C	-2.66802600	4.31228300	-0.16530200
H	-1.68783500	2.95708500	-1.53740300
C	-3.30744700	3.36935000	1.96488700
H	-2.79160500	1.28707500	2.25407100
C	-3.25680700	4.45710600	1.09241200
H	-2.61988600	5.15715300	-0.84487800
H	-3.75510000	3.47770100	2.94758400
H	-3.66739300	5.41554900	1.39328000
C	-3.96574500	-0.54411000	0.00533500
C	-4.84760800	0.49153600	-0.35833800
C	-4.52408600	-1.70929200	0.56454500
C	-6.22149000	0.37009200	-0.16604300
H	-4.45802800	1.40016900	-0.80378500
C	-5.89510700	-1.82498500	0.78573300
H	-3.87016900	-2.53463600	0.83011400
C	-6.74786700	-0.78420600	0.41716600
H	-6.88246500	1.17731300	-0.46710700
H	-6.29861900	-2.72812900	1.23365900
H	-7.81834100	-0.87396900	0.57684100
C	1.90928100	0.94582700	-2.22143900
C	2.49242600	2.11545800	-2.72609300
C	1.22607000	0.12081300	-3.13413500
C	2.39818700	2.45469400	-4.07849700
H	3.03502100	2.76889700	-2.04948800
C	1.11983200	0.45164400	-4.48245900

H	0.75869500	-0.79622400	-2.78020600
C	1.70845200	1.62534700	-4.96102800
H	2.86314200	3.36753300	-4.44137000
H	0.58346200	-0.20458300	-5.16287400
H	1.63145500	1.88647000	-6.01276200

23) **TSA[8-9]**

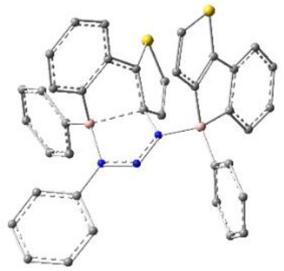

(Hydrogens are omitted for clarity)

Number of imaginary frequencies = 1 (76.2 cm^{-1})

E_{total} = -2474.890381 a.u

$G_{correction}$ = 0.486553 a.u

Cartesian coordinates:

S	-0.53722200	-3.20371400	-0.83345800
S	-3.79217300	-1.88750900	1.91644700
N	-0.31057700	0.66296400	-0.28859300
B	-1.88824900	1.03000100	-0.16868500
N	1.80580500	1.03194900	0.01602200
N	0.59293400	1.52290800	-0.01160700
C	-2.64362300	0.20452000	-1.36243900
C	-2.42449000	0.18720400	1.10963800
C	-3.31742700	-0.92115300	-0.81325200
C	-2.71059400	0.40472000	-2.73557800
H	-2.24091400	1.28004900	-3.17891100
C	0.12080200	-0.68436600	-0.68375300
C	-2.34859500	0.18405100	2.53355000
H	-1.80446800	0.91823700	3.11750500

C	-0.00607600	-1.00930700	-2.10248800
H	0.12383700	-0.27136700	-2.88130400
C	1.39259400	-1.00392300	1.59713300
C	-3.97401500	-1.84643300	-1.62130400
H	-4.47555200	-2.70831400	-1.18910900
C	-0.32232000	-2.29881900	-2.32735900
H	-0.49324800	-2.79552700	-3.27089300
C	-0.20225200	-1.82709500	0.12573600
C	1.97647300	-0.82425000	2.85107800
H	2.84516500	-0.18183700	2.96436300
C	1.45969600	-1.50150900	3.95883600
H	1.92415900	-1.37446500	4.93217700
C	-3.16655000	-0.88272200	0.63919900
C	-3.37537400	-0.51572600	-3.56052500
H	-3.41779900	-0.35295400	-4.63392400
C	0.25017600	-1.83074300	1.50003000
C	-3.98667600	-1.64074400	-3.00692300
H	-4.49721200	-2.35156200	-3.65050400
C	-0.25752300	-2.53697400	2.59435700
H	-1.13309200	-3.16684700	2.48931700
C	0.36040500	-2.36063600	3.82847700
H	-0.02264700	-2.88051200	4.70072600
C	-3.03310300	-0.85519800	3.11051800
H	-3.13789600	-1.09145300	4.16038600
B	1.85510500	-0.50533000	0.14777000
C	-2.06857400	2.62046600	-0.18375500
C	-1.49132400	3.41653300	-1.18990500
C	-2.86230000	3.27658900	0.76782300
C	-1.68726000	4.79512800	-1.24244000
H	-0.86401100	2.94829500	-1.94550900
C	-3.07040000	4.65735200	0.72489000
H	-3.32991800	2.69285800	1.55549700
C	-2.48111700	5.42326000	-0.27947300

H	-1.22595300	5.38079200	-2.03353500
H	-3.69150700	5.13470900	1.47824600
H	-2.63786600	6.49764800	-0.31490200
C	2.86193900	1.95612100	0.21205600
C	4.11134700	1.48387800	0.63032000
C	2.66145500	3.32549600	-0.01177400
C	5.15606200	2.38490900	0.82685400
H	4.26723000	0.42377800	0.78745300
C	3.71249400	4.21162500	0.20012000
H	1.68886500	3.67781100	-0.33423400
C	4.96357200	3.74969500	0.61637600
H	6.12347400	2.01271700	1.14874000
H	3.55265700	5.27189000	0.03114600
H	5.77987400	4.44750100	0.77173200
C	2.93069600	-1.30682400	-0.66673900
C	3.36347000	-2.57503700	-0.24616200
C	3.44956900	-0.80688600	-1.87493900
C	4.27374100	-3.31711800	-0.99945500
H	2.98424200	-2.98148100	0.68692100
C	4.36320300	-1.53715400	-2.62901100
H	3.13365400	0.17294800	-2.22341800
C	4.77698200	-2.79846900	-2.19207700
H	4.59322200	-4.29591200	-0.65383900
H	4.75442900	-1.12733100	-3.55560600
H	5.48966700	-3.37096100	-2.77836500

24) **A9**

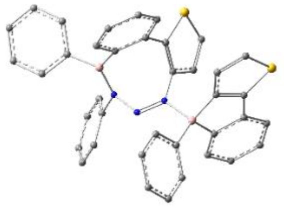

(Hydrogens are omitted for clarity)

Number of imaginary frequencies = 0

E_{total} = -2474.890425 a.u

$G_{correction}$ = 0.485693 a.u

Cartesian coordinates:

S	-0.65049800	-3.17647700	-0.78373700
S	-3.76921700	-1.88144400	1.93683000
N	-0.30621000	0.67168600	-0.29855600
B	-1.88028800	1.03276800	-0.16861000
N	1.80753700	1.03592900	-0.00488900
N	0.59941500	1.53319800	-0.02998300
C	-2.64665200	0.20510000	-1.35420200
C	-2.40687600	0.19217000	1.11532400
C	-3.32100700	-0.91644400	-0.79752700
C	-2.71897000	0.39934000	-2.72752400
H	-2.24766200	1.27053900	-3.17705600
C	0.14935800	-0.68343200	-0.66909600
C	-2.31803700	0.19054100	2.53826400
H	-1.76768700	0.92475800	3.11620000
C	-0.01131100	-1.02772200	-2.08737700
H	0.15254200	-0.30973200	-2.87870300
C	1.41167200	-1.00295100	1.57518100
C	-3.98415500	-1.84352700	-1.59798900
H	-4.48648100	-2.70178700	-1.15956600
C	-0.39755900	-2.29832000	-2.29310400
H	-0.60485200	-2.79813500	-3.22767800
C	-0.23100300	-1.81373300	0.15595400
C	2.03631500	-0.83411000	2.81043900
H	2.91667400	-0.20373500	2.89741500
C	1.54569000	-1.50586800	3.93348600
H	2.04272500	-1.38613100	4.89161500
C	-3.15617600	-0.87727000	0.65296600
C	-3.38998400	-0.52332800	-3.54553900

H	-3.43576700	-0.36556400	-4.61954000
C	0.25601700	-1.81816000	1.51475500
C	-4.00221800	-1.64405100	-2.98489100
H	-4.51778300	-2.35602600	-3.62309300
C	-0.22596500	-2.52030000	2.62506100
H	-1.10931800	-3.14302700	2.54628000
C	0.43194600	-2.35189700	3.83867200
H	0.07180700	-2.86844900	4.72255300
C	-2.99686100	-0.84830100	3.12249900
H	-3.09118400	-1.08391700	4.17353300
B	1.82500000	-0.51160500	0.10235400
C	-2.07001200	2.62249000	-0.18322100
C	-1.50044900	3.42157400	-1.19122500
C	-2.86516300	3.27417400	0.77034400
C	-1.70507900	4.79892200	-1.24362200
H	-0.87187500	2.95692400	-1.94787500
C	-3.08173400	4.65357900	0.72764300
H	-3.32719700	2.68822800	1.55971200
C	-2.49999300	5.42264200	-0.27872300
H	-1.24917800	5.38708600	-2.03596600
H	-3.70348300	5.12740200	1.48270500
H	-2.66324200	6.49606200	-0.31400900
C	2.87147700	1.94659500	0.20265100
C	4.12137700	1.45345400	0.59491100
C	2.68112300	3.32316200	0.01557000
C	5.17575900	2.34047500	0.80362500
H	4.26935500	0.38831600	0.72125700
C	3.74215800	4.19453200	0.23775800
H	1.70850700	3.69173000	-0.28797900
C	4.99340500	3.71168700	0.62945400
H	6.14327800	1.95188000	1.10526100
H	3.59004300	5.26006400	0.09690700
H	5.81737700	4.39841100	0.79358200

C	2.91227200	-1.30996400	-0.71144400
C	3.36074900	-2.57107700	-0.28799600
C	3.42059200	-0.81107800	-1.92415800
C	4.27443400	-3.30798600	-1.04294500
H	2.99154900	-2.97759500	0.64921300
C	4.33779400	-1.53541300	-2.68020200
H	3.09474300	0.16489700	-2.27505200
C	4.76644300	-2.79071800	-2.24068900
H	4.60541900	-4.28188900	-0.69402600
H	4.72044400	-1.12500000	-3.61021800
H	5.48167000	-3.35880200	-2.82818100

25) **TSA[9-10]**

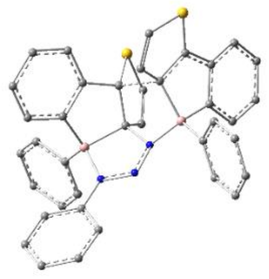

(Hydrogens are omitted for clarity)

Number of imaginary frequencies = 1 (308.2 cm^{-1})

E_{total} = -2474.871926 a.u

$G_{correction}$ = 0.485809 a.u

Cartesian coordinates:

S	-0.91556400	-3.09287600	0.09235700
S	-4.05427300	-1.21010800	2.12326900
N	-0.18879600	0.58547500	-0.74138700
B	-1.71196500	0.73639900	-0.45670400
N	1.85830300	1.09315200	-0.30283800
N	0.66378600	1.53279200	-0.54566100
C	-2.62765900	-0.17704200	-1.44508600

C	-1.94569300	-0.14775900	0.95552300
C	-3.45760600	-1.04696600	-0.68568400
C	-2.76460200	-0.19340000	-2.83043700
H	-2.15707600	0.46516300	-3.44503100
C	0.34424200	-0.78379100	-0.59385500
C	-1.87961700	0.20572800	2.36403600
H	-1.06893900	0.78116300	2.79300400
C	0.13580600	-1.62340900	-1.81008800
H	0.48413800	-1.29119700	-2.77991900
C	1.76234500	-0.81808500	1.51509500
C	-4.39425900	-1.90386800	-1.28245900
H	-5.01341300	-2.56018100	-0.67774100
C	-0.45569700	-2.79893900	-1.60081900
H	-0.67000400	-3.57270900	-2.32604500
C	-0.34934700	-1.47429700	0.59091500
C	2.68344100	-0.70580100	2.56256100
H	3.65520000	-0.25706200	2.38091200
C	2.37137200	-1.17933300	3.83728400
H	3.09516400	-1.08721300	4.64194600
C	-3.14802500	-0.86696800	0.70594500
C	-3.68839800	-1.04944800	-3.43474600
H	-3.79211500	-1.05321200	-4.51625800
C	0.51066500	-1.40462900	1.79663200
C	-4.49661800	-1.90431700	-2.66800800
H	-5.20556100	-2.56195400	-3.16060700
C	0.20096700	-1.91535400	3.06279500
H	-0.75826800	-2.38781100	3.24719800
C	1.13717300	-1.79496800	4.08402100
H	0.91158300	-2.18027100	5.07363700
C	-2.89052600	-0.30179000	3.10740400
H	-3.05892300	-0.22071900	4.17255000
B	1.90852500	-0.48110100	-0.05818500
C	-2.22327800	2.25429800	-0.39434500

C	-1.80881100	3.17977900	-1.36902700
C	-3.15444400	2.70132600	0.55380800
C	-2.28488000	4.48963500	-1.38545600
H	-1.09157500	2.87255500	-2.12488100
C	-3.64133900	4.00978800	0.54503600
H	-3.50795900	2.01713900	1.31954400
C	-3.20514700	4.91181400	-0.42420000
H	-1.93957400	5.18163200	-2.14896800
H	-4.35999900	4.32419000	1.29708300
H	-3.57841300	5.93182500	-0.43302400
C	3.04196600	-1.23997900	-0.88427100
C	3.44020600	-0.78782600	-2.15489500
C	3.62427900	-2.42665600	-0.41349400
C	4.37479600	-1.48239600	-2.92012600
H	3.01542800	0.13406500	-2.54667400
C	4.55820800	-3.13351000	-1.17335300
H	3.34082500	-2.80249500	0.56557700
C	4.93778100	-2.66291100	-2.43000200
H	4.66755800	-1.10414700	-3.89583500
H	4.99184700	-4.05006400	-0.78265800
H	5.66750800	-3.20773600	-3.02199900
C	2.85908600	2.06037100	-0.04356900
C	2.56918500	3.43329100	-0.05098800
C	4.15855400	1.62031400	0.23512100
C	3.57940200	4.34809500	0.22443600
H	1.56053900	3.76455200	-0.26403800
C	5.15842300	2.55086100	0.51236400
H	4.38474400	0.56262200	0.21283100
C	4.87761800	3.91596100	0.50926300
H	3.34849900	5.40895800	0.21961200
H	6.16331700	2.20008800	0.72567400
H	5.65960800	4.63723400	0.72374800

26) **A10**

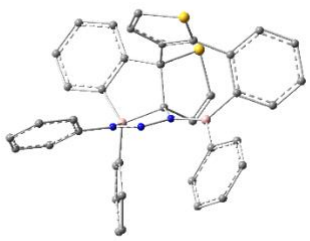

(Hydrogens are omitted for clarity)

Number of imaginary frequencies = 0

E_{total} = -2474.935108 a.u

$G_{correction}$ = 0.488512 a.u

Cartesian coordinates:

S	1.03574900	-3.03266800	1.43170200
S	2.74158700	-1.38617000	-2.84364500
N	0.55289200	0.57109900	0.20316900
B	1.94648900	0.99063100	0.32893800
N	-1.43019900	0.79276800	-0.60325600
N	-0.22287500	1.20664500	-0.66444900
C	3.03658300	-0.06389000	0.68223900
C	0.87755400	-1.83798700	-1.10640400
C	3.17792200	-1.23998600	-0.09100200
C	3.99850300	0.19946700	1.67319000
H	3.91351300	1.11004400	2.25889700
C	-0.13535600	-0.54919900	0.90340800
C	0.27037000	-1.93986500	-2.40235300
H	-0.77661600	-2.17098000	-2.54545900
C	0.29520100	-0.66319900	2.32670200
H	0.10868100	0.15205300	3.01632000
C	-2.32021700	-1.59636300	0.17186500
C	4.27261600	-2.08909900	0.11848300
H	4.37430700	-2.97695800	-0.49704500
C	0.83551000	-1.82914900	2.69479600

H	1.16116700	-2.08405400	3.69647600
C	0.10388400	-1.95865300	0.19029900
C	-3.64198700	-2.01237600	-0.02679000
H	-4.45907500	-1.31608900	0.14109200
C	-3.92234900	-3.31959500	-0.42378500
H	-4.95090600	-3.63486100	-0.57450700
C	2.21932100	-1.52744000	-1.18138900
C	5.05349200	-0.67508600	1.91259700
H	5.77382100	-0.45885300	2.69562400
C	-1.28984700	-2.52615200	-0.02379800
C	5.19669900	-1.81838100	1.12215200
H	6.02892500	-2.49577200	1.28755500
C	-1.55791000	-3.83945600	-0.41578000
H	-0.74720100	-4.54891500	-0.55348000
C	-2.87967700	-4.23177900	-0.61976700
H	-3.10026400	-5.25127500	-0.92216000
C	1.14508200	-1.72485300	-3.42921700
H	0.95012900	-1.76341400	-4.49145500
B	-1.72928200	-0.18977200	0.63187900
C	-2.47343600	0.62964000	1.79159100
C	-2.40764200	2.02873600	1.90422500
C	-3.15314800	-0.06225500	2.80846900
C	-2.98895300	2.70887200	2.97469400
H	-1.90237700	2.60557900	1.13257000
C	-3.73503900	0.60649500	3.88556700
H	-3.22060300	-1.14485000	2.75245200
C	-3.65602600	1.99722500	3.97229600
H	-2.92284400	3.79211100	3.02977200
H	-4.25356300	0.04283600	4.65638700
H	-4.11107900	2.52131300	4.80773200
C	-2.32808600	1.26986400	-1.58812600
C	-1.85895300	1.67134600	-2.84489300
C	-3.69496100	1.29050100	-1.29366500

C	-2.76785900	2.11597400	-3.79945700
H	-0.79874200	1.61317100	-3.05932200
C	-4.59222900	1.73501800	-2.26084700
H	-4.03531000	0.98896200	-0.31196300
C	-4.13468100	2.14929700	-3.51264100
H	-2.40857500	2.42467300	-4.77597800
H	-5.65279200	1.75880700	-2.03248900
H	-4.83966600	2.48947900	-4.26437200
C	2.31174800	2.47822900	0.09291600
C	1.39152000	3.52247700	0.31824100
C	3.60930600	2.82896600	-0.33104000
C	1.74751200	4.85346500	0.12284500
H	0.39335900	3.28750800	0.67203800
C	3.96453900	4.15750300	-0.54915600
H	4.33859700	2.04329500	-0.50037800
C	3.03319500	5.17213800	-0.32005900
H	1.02679500	5.64263100	0.31434500
H	4.96479500	4.40432100	-0.89197000
H	3.31018200	6.21005900	-0.48035000

27) **1,2-Azaborinine**

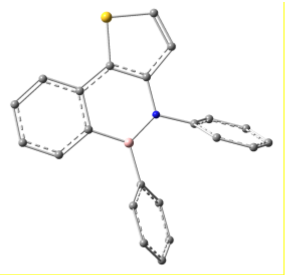

(Hydrogens are omitted for clarity)

Number of imaginary frequencies = 0

E_{total} = -1325.977127 a.u

$G_{correction}$ = 0.272855 a.u

Cartesian coordinates:

S	3.75837400	-1.79050100	0.14760700
N	-0.02084200	-0.69807000	0.01104900
C	1.20066600	-1.37629600	0.06518000
C	1.37148400	-2.79448000	0.18607700
H	0.55273100	-3.49883300	0.23389500
C	1.27828700	1.48135200	-0.09296300
C	2.68594100	-3.15462300	0.23669100
H	3.09368500	-4.15140300	0.32476400
C	2.40420500	-0.68267800	0.03813700
C	1.35805300	2.88514700	-0.21248900
H	0.43631900	3.45456300	-0.26993600
C	2.57595100	3.54512600	-0.26455400
H	2.61066700	4.62628400	-0.35603500
C	2.49988700	0.74749000	-0.05132000
C	3.73472700	1.42701600	-0.10347600
H	4.66291900	0.86385200	-0.06770000
C	3.76892600	2.80697700	-0.20449200
H	4.72572600	3.31940200	-0.24475400
B	-0.07258600	0.73674700	-0.04526000
C	-1.21400900	-1.50048800	0.03155100
C	-2.04987300	-1.47597900	1.14745700
C	-1.54423000	-2.28465400	-1.07608000
C	-3.22433600	-2.22682700	1.14959200
H	-1.78187000	-0.85435200	1.99419200
C	-2.71524500	-3.04106200	-1.06628000
H	-0.88446500	-2.28831400	-1.93771600
C	-3.55919600	-3.01091800	0.04513800
H	-3.87797100	-2.19777000	2.01560400
H	-2.97200500	-3.64601700	-1.93029800
H	-4.47425000	-3.59465300	0.04930000
C	-1.45746100	1.47628600	-0.04957300
C	-2.45648400	1.20594500	-1.00122800

C	-1.73887400	2.45113200	0.92446300
C	-3.67768200	1.87637200	-0.98405600
H	-2.27883900	0.45328000	-1.76330600
C	-2.96477300	3.11651700	0.95780700
H	-0.98738700	2.68635800	1.67345700
C	-3.93767800	2.83197300	-0.00005700
H	-4.42954500	1.65048200	-1.73472700
H	-3.15889800	3.85722100	1.72830100
H	-4.89168200	3.35070300	0.01906200

28) **Dinitrogen**

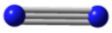

Number of imaginary frequencies = 0

E_{total} = -109.524881 a.u

$G_{correction}$ = -0.012851 a.u

Cartesian coordinates:

N 0.00000000 0.00000000 0.55274900

N 0.00000000 0.00000000 -0.55274900

4.1.4 Different boroles probed for antiaromaticity

Table S1. NICS values for selected borole derivatives

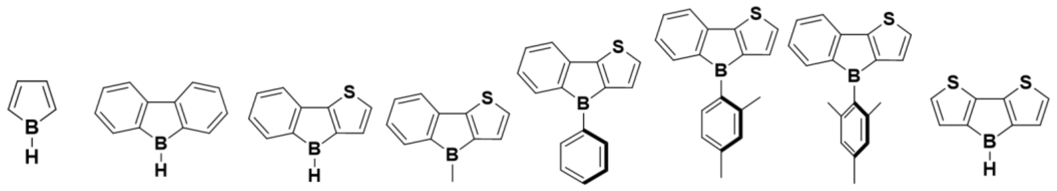

	A	B	C	D	E	F	G	H
NICS(0)	20.71	16.21	18.46	16.51	16.19	16.31	16.81	23.11
NICS(1)	11.83	7.10	9.61	8.03	8.24	8.15	7.81	14.44
NICSzz(1)	**38.06**	**27.19**	**34.78**	**30.57**	**30.35**	**30.70**	**29.92**	**48.78**

29) Compound **A**

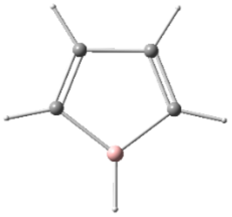

B	0.00019500	1.31782900	0.00008800
C	-1.25525300	0.35161200	-0.00027100
C	1.25539600	0.35136900	-0.00017300
C	-0.75916500	-0.90121400	0.00021300
C	0.75888100	-0.90131600	-0.00003400
H	0.00005700	2.50976000	0.00112300
H	-2.31590100	0.57623300	-0.00043000
H	-1.33090200	-1.82659500	0.00039800
H	1.33048600	-1.82680000	0.00023100
H	2.31613000	0.57554700	-0.00016700

30) Compound **B**

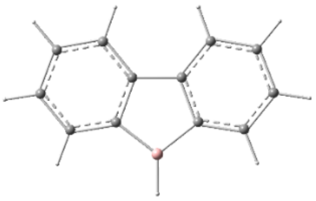

B	0.00000000	1.93035600	0.00000000
C	1.23294300	0.97309600	0.00000000
C	0.74422700	-0.36398800	0.00000000
C	2.60976300	1.19559000	0.00000000
H	2.99766200	2.21099900	0.00000000
C	1.61613200	-1.44406500	0.00000000
H	1.24839300	-2.46643800	0.00000100
C	3.49740900	0.10734400	0.00000000
H	4.57010400	0.27571100	0.00000000
C	3.00029800	-1.19675200	0.00000000
H	3.69197800	-2.03456800	0.00000100
H	0.00000000	3.12213100	0.00000000
C	-1.23294300	0.97309600	0.00000000
C	-2.60976300	1.19559000	0.00000100
C	-0.74422700	-0.36398800	0.00000000
C	-3.49740900	0.10734400	0.00000000
H	-2.99766200	2.21099900	0.00000100
C	-1.61613200	-1.44406500	0.00000000
C	-3.00029800	-1.19675200	0.00000000
H	-4.57010400	0.27571100	0.00000000
H	-1.24839300	-2.46643800	-0.00000100
H	-3.69197800	-2.03456800	0.00000000

31) Compound **C**

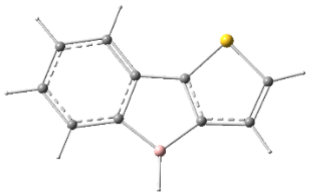

S	2.05070100	-1.32129200	0.00016400
B	-0.05156300	2.03645800	0.00008900
C	-1.25174600	1.01252500	0.00002200
C	1.21651300	1.14138000	0.00006000

C	-0.70311900	-0.30611000	-0.00008600
C	-2.63139500	1.16247600	-0.00000800
H	-3.07341800	2.15543600	-0.00007700
C	2.64475300	1.20779900	-0.00002300
H	3.21668800	2.12885800	-0.00005500
C	-1.51115400	-1.42909400	-0.00011900
H	-1.08915500	-2.43005300	-0.00017300
C	0.76694000	-0.17872000	-0.00005500
C	-3.46737500	0.02472300	0.00001600
H	-4.54646100	0.14387100	0.00007500
C	-2.91119900	-1.24960000	-0.00002100
H	-3.55896500	-2.12126000	0.00005100
C	3.23647000	-0.02981000	-0.00022600
H	4.28906700	-0.27713400	-0.00044700
H	-0.12329200	3.22524300	0.00020000

32) Compound **D**

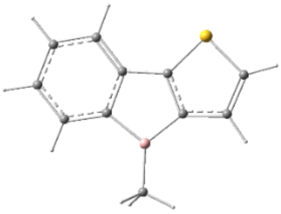

S	2.10467700	-1.58897800	0.00021100
B	-0.07634200	1.73303700	0.00048600
C	-1.25321800	0.66551800	0.00009600
C	1.21241000	0.85391000	0.00064600
C	-0.67157100	-0.63662400	-0.00041800
C	-2.63636400	0.77772700	0.00035400
H	-3.10691200	1.75805400	0.00046900
C	2.63824100	0.95338200	0.00029200
H	3.19010100	1.88688300	0.00041900
C	-1.45040100	-1.78211800	-0.00038600
H	-1.00063100	-2.77097700	-0.00055500

C	0.79385100	-0.47381400	-0.00007300
C	-3.44232300	-0.38035700	0.00017800
H	-4.52427400	-0.28936300	0.00037700
C	-2.85292900	-1.64083000	-0.00014100
H	-3.47786200	-2.52903600	-0.00013700
C	3.25927100	-0.27042200	-0.00049700
H	4.31762900	-0.49217000	-0.00097900
C	-0.22814400	3.28897400	-0.00028100
H	-0.80423800	3.61246600	-0.87903900
H	-0.81850700	3.61374300	0.86835300
H	0.71861400	3.83678000	0.00666900

33) Compound **E**

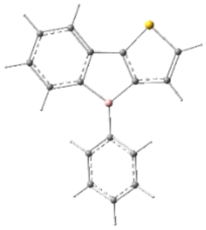

S	-3.02038900	-1.94315800	0.12315300
B	0.45178500	-0.01577700	-0.02477300
C	-0.52422100	1.23784800	-0.05417600
C	-0.51648700	-1.24199600	-0.00045100
C	-1.86382300	0.74628600	-0.01770900
C	-0.32787800	2.61015400	-0.14956000
H	0.67562400	3.02027900	-0.21424900
C	-0.52749800	-2.67067100	0.07160700
H	0.35807300	-3.29573900	0.08292400
C	-2.95651200	1.59781500	-0.04635500
H	-3.97161000	1.21192400	-0.01785300
C	-1.80784300	-0.72423100	0.02616900
C	-1.43100000	3.48814600	-0.17835200
H	-1.26832400	4.55936500	-0.24797100
C	-2.72710600	2.98561800	-0.12211300

H	-3.57284700	3.66648100	-0.14478500
C	-1.79423500	-3.19413100	0.13553000
H	-2.09408800	-4.23143400	0.19302300
C	1.99667200	-0.03229500	-0.00305000
C	2.70974700	-1.14911300	-0.49028300
C	2.75029100	1.04817700	0.50368700
C	4.10346000	-1.17995700	-0.49229300
H	2.15870300	-1.99517500	-0.88975600
C	4.14408800	1.01486200	0.52846200
H	2.23461700	1.91394400	0.90625500
C	4.82348800	-0.09754100	0.02272500
H	4.62928200	-2.04491300	-0.88587500
H	4.70165600	1.85289200	0.93636100
H	5.90936200	-0.12204800	0.03232000

34) Compound **F**

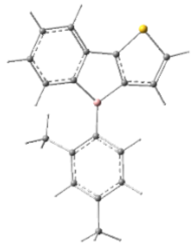

S	-3.49677000	-2.01219200	0.17721600
B	-0.03727100	-0.05635700	0.02476000
C	-1.02515100	1.17870700	-0.13947900
C	-1.00051300	-1.28531800	0.09641500
C	-2.36131100	0.67676900	-0.12956800
C	-0.83101900	2.53687900	-0.35593600
H	0.17425000	2.94534700	-0.39611000
C	-0.99403600	-2.70399500	0.27292400
H	-0.09786700	-3.30802800	0.35982100
C	-3.45574400	1.51103000	-0.29327700
H	-4.46874500	1.11874200	-0.28394900
C	-2.29659800	-0.78510400	0.03565500

C	-1.93509600	3.39647000	-0.52696800
H	-1.77525200	4.45798000	-0.68988200
C	-3.22996600	2.88783800	-0.48736200
H	-4.07721100	3.55488000	-0.61650600
C	-2.25597600	-3.24062100	0.32783400
H	-2.54601800	-4.27510500	0.45042300
C	1.50944100	-0.10994900	0.02750200
C	2.10089900	-1.13849800	-0.74352700
C	2.37637500	0.79238000	0.68924900
C	3.47670000	-1.24259500	-0.90963500
H	1.45511000	-1.85767000	-1.23986600
C	3.76191700	0.65659300	0.53209300
C	4.33406900	-0.33501800	-0.27048400
H	3.89225100	-2.03631400	-1.52538400
H	4.41792400	1.34477800	1.06220300
C	5.82909500	-0.42838200	-0.44805400
H	6.13082500	-0.03515900	-1.42667500
H	6.35981100	0.14447700	0.31754300
H	6.17148400	-1.46718600	-0.39671800
C	1.87091600	1.87002600	1.62357000
H	2.08673900	2.87050800	1.23006000
H	0.79668900	1.80125200	1.79434600
H	2.37066400	1.79508000	2.59571500

35) Compound **G**

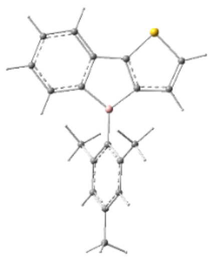

S	-3.60134300	-1.88680500	0.33035300
B	-0.09902200	-0.03575000	0.01529100

C	-1.05555200	1.21496500	-0.17845800
C	-1.09044900	-1.22839900	0.18640500
C	-2.40468500	0.75838500	-0.11577900
C	-0.81139900	2.55837300	-0.42363900
H	0.21134500	2.92229500	-0.48630900
C	-1.11497200	-2.63688300	0.42587400
H	-0.23085500	-3.25724100	0.52304900
C	-3.47180200	1.62638100	-0.27832500
H	-4.49854400	1.27457900	-0.23174300
C	-2.37509900	-0.69757500	0.11713800
C	-1.88885700	3.45330600	-0.58981600
H	-1.69751400	4.50549200	-0.77711400
C	-3.19899400	2.98949000	-0.51465600
H	-4.02485400	3.68286300	-0.64358500
C	-2.38849100	-3.13825600	0.52335500
H	-2.70273100	-4.15826400	0.69692800
C	1.45511800	-0.04747600	0.01789900
C	2.16398800	-0.67457400	-1.03404500
C	2.18440600	0.56985400	1.05924600
C	3.56122800	-0.66387900	-1.03418700
C	3.58317600	0.54899700	1.03561900
C	4.29132200	-0.06131400	-0.00351800
H	4.09351300	-1.13473900	-1.85817400
H	4.13235600	1.02047300	1.84785800
C	1.41942500	-1.31407900	-2.18537300
H	0.69279800	-2.05413300	-1.83471300
H	0.85924500	-0.56747800	-2.76143800
H	2.10787400	-1.81403300	-2.87231800
C	1.46718200	1.20303400	2.23112900
H	0.69361800	1.90419000	1.90377300
H	0.96890500	0.44303600	2.84561400
H	2.16488700	1.74387600	2.87657200
C	5.80038000	-0.09560300	-0.00311800

H	6.21681900	0.69320300	0.63031000
H	6.17157500	-1.05518500	0.37817200
H	6.20261500	0.03021600	-1.01348700

36) Compound **H**

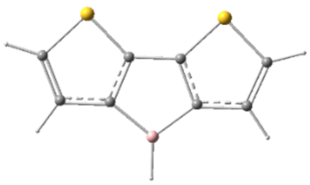

S	-1.95840500	-1.33106500	0.00000000
B	0.00000000	2.13410800	-0.00000200
C	-1.24072000	1.16943300	0.00000000
C	-2.66555300	1.16704800	0.00000200
H	-3.28164500	2.05925300	0.00000200
C	-0.73387300	-0.12993500	0.00000000
C	-3.20187100	-0.10036200	0.00000000
H	-4.24135300	-0.39680400	0.00000000
H	0.00000000	3.32443200	0.00000000
C	1.24072000	1.16943300	-0.00000100
C	0.73387300	-0.12993500	-0.00000100
C	2.66555300	1.16704800	0.00000100
S	1.95840500	-1.33106500	0.00000000
C	3.20187100	-0.10036200	0.00000100
H	4.24135300	-0.39680400	0.00000100
H	3.28164500	2.05925300	0.00000100

5. References

1 N. N. Pham, S. Parpart, S. Grigoryan, T. N. Ngo, T. T. Dang, T. V. Ghochikyan, A. S. Saghyan, P. Ehlers, P. Langer, *Eur. J. Inorg. Chem.*, 2017, 538-550.

2 W. Zhang, D. Yu, Z. Wang, B. Zhang, L. Xu, G. Li, N. Yan, E. Rivard, G. He, *Org. Lett.*, 2019, **21**, 109-113.

3 W. Haubold, J. Herdtle, W. Gollinger, W. Einholz, *J. Organomet. Chem.*, 1986, **315**, 1-8.

4 G. Sheldrick, *Acta Cryst.*, 2015, **A71**, 3–8.

5 G. Sheldrick, *Acta Cryst.*, 2008, **A64**, 112–122.

6 D. Kratzert, J. J. Holstein, I. Krossing, *J. Appl. Cryst.*, 2015, **48**, 933-938.

7 M. J. Frisch, G. W. Trucks, H. B. Schlegel, G. E. Scuseria, M. A. Robb, J. R. Cheeseman, G. Scalmani, V. Barone, G. A. Petersson, H. Nakatsuji, X. Li, M. Caricato, A. V. Marenich, J. Bloino, B. G. Janesko, R. Gomperts, B. Mennucci, H. P. Hratchian, J. V. Ortiz, A. F. Izmaylov, J. L. Sonnenberg, D. Williams-Young, F. Ding, F. Lipparini, F. Egidi, J. Goings, B. Peng, A. Petrone, T. Henderson, D. Ranasinghe, V. G. Zakrzewski, J. Gao, N. Rega, G. Zheng, W. Liang, M. Hada, M. Ehara, K. Toyota, R. Fukuda, J. Hasegawa, M. Ishida, T. Nakajima, Y. Honda, O. Kitao, N. Nakai, T. Vreven, K. Throssell, J. A. Montgomery, Jr., J. E. Peralta, F. Ogliaro, M. J. Bearpark, J. J. Heyd, E. N. Brothers, K. N. Kudin, V. N. Staroverov, T. A. Keith, R. Kobayashi, J. Normand, K. Raghavachari, A. P. Rendell, J. C. Burant, S. S. Iyengar, J. Tomasi, M. Cossi, J. M. Millam, M. Klene, M.; C. Adamo, R. Cammi, J. W. Ochterski, R. L. Martin, K. Morokuma, O. Farkas, J. B. Foresman, D. J. Fox, Gaussian 16, Revision E.01; Gaussian, Inc., Wallingford CT, 2016.

8 (a) A. D. Becke, *Phys. Rev. A*, 1988, **38**, 3098-3100; (b) A. D. Becke, *J. Chem. Phys.*, 1993, **98**, 5648-5652; (c) C. Lee, W. Yang, R. G. Parr, *Phys. Rev. B*, 1988, **37**, 785-789.

9 (a) S. Grimme, J. Antony, S. Ehrlich, H. J. Krieg, *Chem. Phys.*, 2010, **132**, 154104; (b) S. Grimme, S. Ehrlich, L. Goerigk, *J. Comput. Chem.*, 2011, **32**, 1456–1465.

10 J. Baker, *J. Comput. Chem.*, 1986, **7**, 385-395.

11 (a) A. L. L. East, G. M. Berner, A. D. Morcom, L. Mihichuk, *J. Chem. Theory Comput.*, 2008, **4**, 1274-1282; (b) A. Jayaraman, G. M. Berner, L. M. Mihichuk, A. L. L. East, *J. Mol. Catal. A: Chem.*, 2011, **351**, 143-153; (c) A. Jayaraman, A. L. L. East, *J. Org. Chem.*, 2012, **77**, 351-356; (d) Y. P. Budiman, A. Jayaraman, A. Friedrich, F. Kerner, U. Radius, T. B. Marder, *J. Am. Chem. Soc.*, 2020, **142**, 6036-6050; (e) T. E. Stennett, A. Jayaraman, T. Brückner, L. Schneider, H. Braunschweig, *Chem. Sci.*, 2020, **11**, 1335-1341.

12 A. V. Marenich, C. J. Cramer, D. G. Truhlar, *J. Phys. Chem. B*, 2009, **113**, 6378-6396.

13 (a) P. v. R. Schleyer, C. Maerker, A. Dransfeld, H. Jiao, N. J. R. van Eikema Hommes, *J. Am. Chem. Soc.*, 1996, **118**, 6317-6318; (b) Z. Chen, C. S. Wannere, C. Corminboeuf, R. Puchta, P. v. R. Schleyer, *Chem. Rev.*, 2005, **105**, 3842-3888; (c) A. Stanger, *J. Org. Chem.*, 2006, **71**, 883-893; (d) A. C. Tsipis, *Phys. Chem. Chem. Phys.*, 2009, **11**, 8244-8261.

14 (a) R. Ditchfield, *Mol. Phys.*, 1974, **27**, 789-807; (b) K. Wolinski, J. F. Hinton, P. Pulay, *J. Am. Chem. Soc.*, 1990, **112**, 8251-8260; (c) J. R. Cheeseman, G. W. Trucks, T. A. Keith, M. J. Frisch, *J. Chem. Phys.*, 1996, **104**, 5497-5509.
